# FRESCo: finding regions of excess synonymous constraint in diverse viruses

**DOI:** 10.1186/s13059-015-0603-7

**Published:** 2015-02-17

**Authors:** Rachel S Sealfon, Michael F Lin, Irwin Jungreis, Maxim Y Wolf, Manolis Kellis, Pardis C Sabeti

**Affiliations:** MIT, Computer Science and Artificial Intelligence Laboratory, Cambridge, MA 02139 USA; Broad Institute, Cambridge, MA 02142 USA; DNANexus, Mountain View, CA 94040 USA; Department of Organismic and Evolutionary Biology, Harvard University, Cambridge, MA 02138 USA

## Abstract

**Background:**

The increasing availability of sequence data for many viruses provides power to detect regions under unusual evolutionary constraint at a high resolution. One approach leverages the synonymous substitution rate as a signature to pinpoint genic regions encoding overlapping or embedded functional elements. Protein-coding regions in viral genomes often contain overlapping RNA structural elements, reading frames, regulatory elements, microRNAs, and packaging signals. Synonymous substitutions in these regions would be selectively disfavored and thus these regions are characterized by excess synonymous constraint. Codon choice can also modulate transcriptional efficiency, translational accuracy, and protein folding.

**Results:**

We developed a phylogenetic codon model-based framework, FRESCo, designed to find regions of excess synonymous constraint in short, deep alignments, such as individual viral genes across many sequenced isolates. We demonstrated the high specificity of our approach on simulated data and applied our framework to the protein-coding regions of approximately 30 distinct species of viruses with diverse genome architectures.

**Conclusions:**

FRESCo recovers known multifunctional regions in well-characterized viruses such as hepatitis B virus, poliovirus, and West Nile virus, often at a single-codon resolution, and predicts many novel functional elements overlapping viral genes, including in Lassa and Ebola viruses. In a number of viruses, the synonymously constrained regions that we identified also display conserved, stable predicted RNA structures, including putative novel elements in multiple viral species.

**Electronic supplementary material:**

The online version of this article (doi:10.1186/s13059-015-0603-7) contains supplementary material, which is available to authorized users.

## Background

The growing availability of sequence data for many viral species creates an opportunity for sensitive and powerful approaches to identify and annotate functional elements in viral genomes. With improving sequencing technologies, the number of isolates sequenced has increased to thousands for some virus species. This in turn provides an opportunity to identify genomic elements under unusual evolutionary constraint.

Synonymous mutations in protein-coding genes have traditionally been regarded as neutral; however, there is mounting evidence that synonymous changes often have significant functional implications. Regions of additional function overlapping protein-coding genes have been described in many different classes of organisms, including bacteria, insects, and mammals [1-6]. Overlapping elements within genic regions are particularly common in viral genomes, which must encode all information necessary to direct entry, replication, packaging, and shedding within strict length constraints. Diverse types of overlapping elements have been identified within viral genes, including microRNAs, overlapping reading frames, transcription factor binding sites, packaging signals, and RNA editing sites [7-11]. Moreover, codon choice can alter mRNA secondary structure and affect transcriptional efficiency [12], translational efficiency [13], translational accuracy, and protein folding dynamics [14].

**Figure 1:**
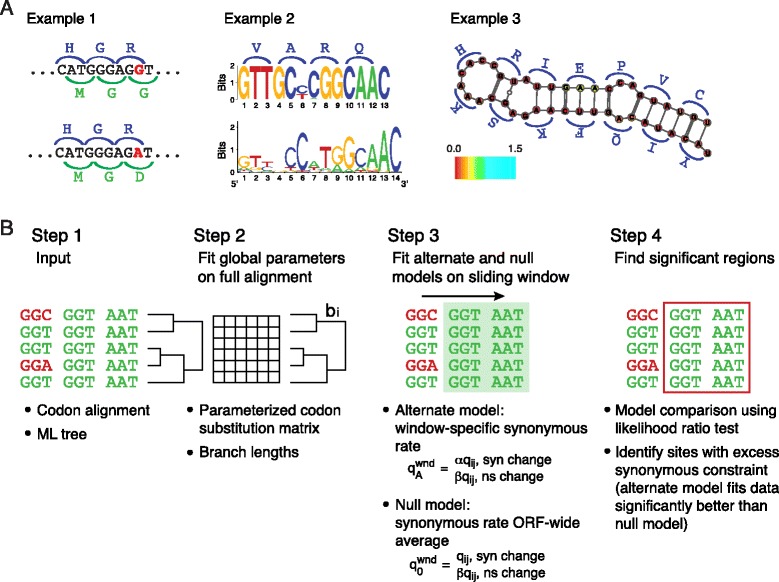
**FRESCo is a codon-model based approach to identify synonymous constraint elements in coding regions. (A)** In a gene also encoding an additional, overlapping function, we expect to observe reduced synonymous variability. Example 1: this sequence fragment from two hepatitis B virus (HBV) isolates overlaps with both the HBV polymerase and the HbsAg genes. The G to A mutation between the two isolates (shown in red) is synonymous with respect to the polymerase gene but nonsynonymous with respect to the overlapping HbsAg gene. Example 2: this region encodes a portion of the HBV polymerase protein and also contains a binding site for the transcription factor RFX1 [8]. Top: sequence motif based on an alignment of 2,000 HBV sequences. Bottom: RFX1 binding motif for *Mus musculus* from the Jaspar database [23]. Example 3: the CRE element in the poliovirus genome is contained within the ORF and has strong, highly conserved secondary structure. Base pairs are colored according to their synonymous substitution rate at a single codon resolution. At a single-codon resolution, each codon in the CRE except the one encoding glutamic acid has a significant signal of excess synonymous constraint. (Glutamic acid is encoded by two codons, GAA and GAG, and both are apparently well-tolerated in the RNA secondary structure, probably due to U-G pairing.) **(B)** Starting with (1) a codon alignment and a phylogenetic tree, we first (2) fit maximum-likelihood global parameters on the full alignment. These parameters include branch lengths and a parameterized codon substitution matrix. We then (3) fit maximum-likelihood local parameters (local synonymous and nonsynonymous substitution rates) across a sliding window. In the null model, the synonymous rate is constrained to 1, while the alternative model allows a window-specific synonymous substitution rate. In each window, we (4) perform model comparison using the likelihood ratio test to identify positions with significantly reduced synonymous variability. ML, maximum likelihood.

In a genic region encoding an overlapping functional element, synonymous substitutions are likely to disrupt the additional element and to be selectively disfavored. Thus, it is possible to scan for overlapping functional elements in genomes by systematically identifying regions of excess synonymous constraint (Figure 1A). Several previous studies have identified this signature in viruses [15-19]. While these methods are valuable, most of these approaches identify regions of excess constraint only at low resolution, and also lack an available implementation. The method of Mayrose and colleagues [18] used a model-comparison framework; however, the models applied differ from those used here, the method is applied only to the HIV genome, and there is no available implementation to our knowledge. There has also been previous work on codon models for other applications that incorporate synonymous rate variation [20-22]. For example, the fixed effect likelihood method of Kosakovsky-Pond and Frost [20], designed to identify amino acid sites under selection, estimates a sitewise synonymous rate. However, this method is not designed to find regions of excess synonymous constraint, and does not include a model comparison step to identify such regions.

In this study, we adapted a phylogenetic, codon-model approach, originally developed for mammalian genomes [3], to create a sensitive method designed to detect regions of overlapping function in short, deeply sequenced alignments, such as viral genes. Our framework is able to efficiently make use of the information present in deep sequence alignments, testing for regions under unusual constraint within a principled statistical model-comparison framework that allows us to identify constrained regions at high resolution (in some cases even a single-codon resolution).

We first demonstrated the specificity of our method on simulated sequence data. We then applied our model to the genomes of diverse viral species, recovering known multifunctional regions and predicting novel overlapping elements. We have made our code for identifying regions of excess constraint available as a HYPHY [24] batch script (Additional file 1), permitting the method to be applied to any alignment of open reading frames (ORFs).

## Results and discussion

### Finding Regions of Excess Synonymous Constraint (FRESCo): a phylogenetic codon-model based approach for detecting regions with reduced synonymous variability

We developed a phylogenetic codon-model based approach for detecting synonymous constraint elements (SCEs) in viruses (Figure 1B). The tiny size of typical viral genomes presents a challenge in designing a framework suitable for this task. If the genic region of a virus is only a few thousand codons long, there may be insufficient information to characterize even individual codon frequencies, let alone to empirically approximate the 61 × 61 matrix of transition probabilities between amino acid encoding codons with sufficient accuracy. Therefore, we used a parameterized model capable of identifying regions of excess constraint on alignments only a few hundred codons long.

Our framework requires only a phylogeny and a sequence alignment as input. We compute the maximum likelihood branch lengths and global model parameters from the full dataset. We then run a sliding window across the ORF, testing for each window whether a model that permits a locally altered synonymous rate provides a better fit for the data than a model that requires a constant synonymous rate across the alignment. Since the models are nested and the more complex model contains one extra parameter (a local synonymous rate), the log likelihood ratio test of the null and alternative models can be approximated by the chi-squared distribution with one degree of freedom. This property provides us with a rigorous statistical test whether each window in a genome has a significantly reduced level of synonymous variability.

### FRESCo displays high specificity in recovering regions of excess synonymous constraint in simulated sequences

We first examined the ability of our approach to recover SCEs in simulated sequences with known evolutionary parameters. To illustrate the output of our method, we simulated an alignment of 1,000 sequences given an input phylogenetic tree and parameterized codon substitution model. This simulated alignment contains a short region of strong synonymous constraint as well as a longer region of weaker synonymous constraint. In real sequence data, a strong, short signal of excess synonymous constraint in the alignment might correspond to an overlapping functional element that is disrupted by most substitutions, such as a short RNA structural element. A long region of weaker excess synonymous constraint might correspond to an extended region in which each synonymous substitution slightly decreases the fitness of the virus (for example, because codons in a particular region are optimized for translational efficiency).

**Figure 2:**
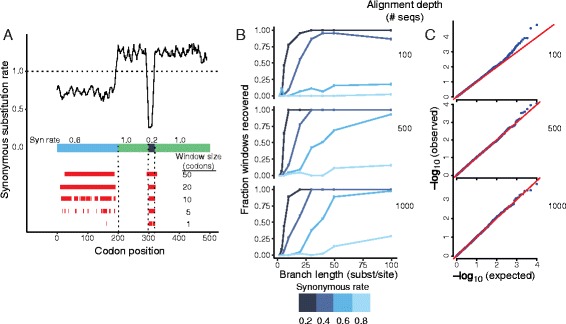
**FRESCo demonstrates high specificity in tests on simulated regions of excess synonymous constraint. (A)** On a simulated dataset of 1,000 sequences with regions of varying strength of synonymous constraint, FRESCo recovers SCEs with high accuracy. We plot the synonymous substitution rate at a 10-codon resolution, displaying below the plot the relative synonymous substitution rate in each portion of the sequence. The red tracks at the bottom show recovered regions of significant excess synonymous constraint at window sizes of 1, 5, 10, 20, and 50 codons. **(B)** Recovery of simulated regions of excess synonymous constraint improves with increasing branch length (in substitutions/site), strength of synonymous constraint, and number of aligned sequences (5-codon sliding windows). **(C)** Distribution of *P*-values in simulated sequence where there is no synonymous constraint. Q-Q plots of the distribution of *P*-values for 5-codon sliding windows in simulations based on alignments of 100 (top), 500 (middle), and 1,000 (bottom) random sequences. Each plot is based on 20 independent, 500-codon simulated alignments (total of 10,000 codons).

In this simulated alignment, FRESCo accurately recovers both the long, weak SCE and the short, strong SCE (Figure 2A). As expected, the short SCE is well captured by smaller sliding windows (and in fact is recovered quite accurately at a single-codon resolution), while the long region of weaker constraint is best recovered at larger window sizes. Outside the regions of synonymous constraint, the estimated synonymous substitution rate is >1, giving an overall genome-wide average synonymous substitution rate normalized to 1.

To systematically probe our method’s ability to recover SCEs with varying alignment depth, strength of constraint, and branch length (Figure 2B), we next simulated alignments of 100, 500, and 1,000 sequences with total branch length ranging from 2 to 100 substitutions per site and with synonymous rate in the constrained region ranging from 0.2 to 0.8 of the rate in the unconstrained region. As expected, FRESCo recovered a higher proportion of the simulated constrained regions for deeper alignments, stronger constraint, and increased branch length. Recovery of constrained regions improves especially dramatically with increasing branch length (more divergent sequences). For example, at a total branch length of 20 substitutions per site and at a synonymous substitution rate of 60% the gene-wide average, we recovered less than 10% of the constrained regions using the 500-sequence alignment. However, when branch length increases to 40 substitutions per site, recovery improves to over 50%. Across all simulations, we recovered no false positives at Bonferroni-corrected significant *P*-values, indicating that our approach is conservative and specific on these simulated datasets. The ability of the method to identify regions of excess synonymous constraint without false positives across a wide range of branch lengths suggests that the method can be applied to alignments spanning a broad range of evolutionary timescales.

In order to test the accuracy of the *P*-values outputted by FRESCo, we also examined the performance of our approach on 30,000 codons of data simulated without any excess synonymous constraint across three separate phylogenies (Figure 2C). We found that FRESCo is highly specific on this dataset, with no windows detected as having excess synonymous constraint at an uncorrected significance cutoff of less than 1e-5 (or at a Bonferroni-corrected significance cutoff of <0.05). Furthermore, the probabilities that each window has excess constraint follow the uniform distribution (with deeper alignments giving *P*-values distributed in a closer approximation to uniformity). Thus, in simulated data without excess synonymous constraint the *P*-values given by the method closely approximate the true null distribution.

### FRESCo recovers regions of known excess synonymous constraint in well-characterized viral genomes: hepatitis B virus, West Nile virus, and poliovirus

**Figure 3:**
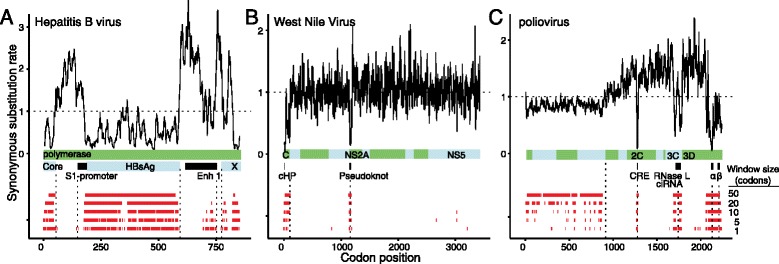
**FRESCo recovers known overlapping functional elements in viral genomes.** For each virus, a plot of the synonymous substitution rate at 10-codon resolution is shown above; the red tracks below each plot display recovered regions of excess synonymous constraint at window sizes of 1, 5, 10, 20, and 50 codons. We plot regions of excess synonymous constraint in **(A)** the HBV P gene, which contains overlapping reading frames and regulatory elements, **(B)** the WNV ORF, which contains overlapping conserved capsid-coding region hairpin and pseudoknot elements, and **(C)** the poliovirus ORF, which contains multiple experimentally characterized regions of overlapping function. cHP, capsid-coding region hairpin element.

We next demonstrated FRESCo’s ability to identify known functional elements in three well-characterized viruses, hepatitis B virus (HBV), West Nile virus (WNV), and poliovirus (Figure 3). These viruses represent excellent test cases for FRESCo both because all three have been extensively sequenced and studied and because they contain genes with many well-annotated overlapping elements. In all three of these viruses, we are able to recover most known overlapping elements at a single-codon resolution (window size of 1; Figure 3).

HBV is a partly double-stranded DNA virus with known overlapping ORFs and regulatory elements, and is responsible for over half a million deaths annually. We obtained over 2,000 whole-genome sequences of the virus from the NCBI database. Applying FRESCo to the HBV polymerase gene, we find that nearly all regions detected at Bonferroni-corrected *P*-values as having excess synonymous constraint lie within previously annotated regions of overlapping function (Figure 3A). We identify strong SCEs corresponding to the overlapping core, HbsAg, and X ORFs. We additionally recover SCEs overlapping the enhancer 1 and pre-S1 promoter elements.

WNV is an RNA virus with a single-stranded positive sense RNA genome with known RNA structural elements. It is an emerging pathogen whose recent spread across North America has been associated with increasing frequency of a neuroinvasive disease in humans. We obtained over 600 whole-genome WNV sequences from NCBI. Applying FRESCo to WNV, we successfully recover both the capsid-coding region hairpin (cHP) element [25] and the pseudoknot element within the NS2A gene [26] (Figure 3B). In the capsid gene, although the strongest signal of excess constraint lies in the known cHP element, the detected region of excess constraint spans the entire length of the capsid, suggesting that synonymous mutations within the capsid but outside of the cHP element may also reduce the fitness of the virus. We additionally detect a weaker signal of excess synonymous constraint within the NS5 gene.

Poliovirus is a single-stranded, positive sense RNA virus with known overlapping elements and experimentally characterized synonymous constraint. Poliovirus was responsible for worldwide epidemics of paralytic poliomyelitis in the first half of the 20th century [27]. We obtained over 300 poliovirus sequences from NCBI. We successfully recover all three of the previously annotated overlapping elements in the poliovirus nonstructural region (the *cis*-acting replication element (CRE) in the 2C gene [28], the RNAse L ciRNA in the 3C gene [29], and the recently discovered α and β elements in the 3D gene [30,31]; Figure 3C). The synonymous substitution rate dips to less than 35% of the genome-wide average in the constrained region in 3C and to less than 10% of the genome-wide average in the constrained region in 2C and 3D. Additionally, although the strongest signal of excess synonymous constraint in 3D corresponds cleanly with the boundary of one of the recently described elements, the SCE in 3D also extends beyond the boundaries of the characterized elements, suggesting that additional functionally important but uncharacterized constraint may be present in this region.

Beyond identifying overlapping elements, we found that the entire structural region of poliovirus is synonymously constrained relative to the non-structural region, consistent with previous functional characterization of the effect of introducing synonymous changes in this region [32,33]. The synonymous substitution rate in the nonstructural region is a mean of 84% the genome-wide rate based on local synonymous rate estimates over 10-codon sliding windows. We note, however, alternatively, that the apparent systematic difference in synonymous substitution rate observed between the structural and nonstructural regions could be due to recombination within the poliovirus genome, since enteroviruses often have distinct phylogenetic trees for their structural and nonstructural regions [34]).

### FRESCo identifies known and novel regions of excess synonymous constraint in 30 virus genomes

We next applied FRESCo to the genomes of a diverse set of viruses with many sequences available in GenBank, including viruses with double- and single-stranded DNA and RNA genomes, plus and minus sense RNA genomes, segmented and unsegmented genomes, and plant, insect, and animal hosts (Additional files 2, 3, 4 and 5).

FRESCo recovered known overlapping functional elements in viral genes with high accuracy (Additional files 3 and 5). These elements include splicing sites in bocavirus; known overlapping genes in bluetongue virus, cucumber mosaic virus, hepatitis E virus, infectious bursal disease virus, maize streak virus, potato virus Y, rotavirus and turnip mosaic virus; RNA structural elements in dengue virus, enterovirus a71, hepatitis A virus, hepatitis C virus, hepatitis E virus, Japanese encephalitis virus, and tick-borne encephalitis virus; likely packaging signals in rotavirus and Venezuelan equine encephalitis virus; and an RNA editing site in Newcastle virus (Additional files 3, 5 and 6).

FRESCo also identified intriguing novel candidates for overlapping functional elements within viral genes. In a number of cases, the SCEs have conserved, stable predicted RNA structures, providing additional support for the presence of overlapping functional elements in these regions (Additional file 7). We describe a set of examples below, and provide information on all identified SCEs in Additional file 3. We further provide plots of the synonymous substitution rate for each gene in Additional file 5, and a table listing known and putative novel constrained elements in Additional file 6.

### Pinpointing regions of excess synonymous constraint near the 5’ and 3’ terminal regions of rotavirus segments

Although rotavirus A is a clinically important virus that contains multiple previously identified SCEs, the exact locations and biological significance of these elements remain incompletely characterized. Rotavirus A is a multi-segmented, double-stranded RNA virus that causes extensive child mortality in the developing world. More than 500 sequences of most rotavirus segments are publicly available in NCBI. The rotavirus NSP5 gene in segment 11 contains the overlapping NSP6 gene in the +1 reading frame [35]. Moreover, previously identified SCEs at the ends of rotavirus segments may function as packaging or translation initiation signals [36].

**Figure 4:**
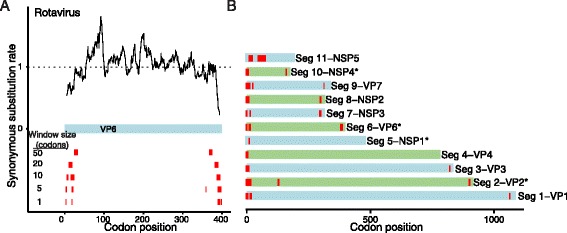
**Regions of excess synonymous constraint in rotavirus genomes. (A)** SCEs in VP6. **(B)** For each segment of the rotavirus genome, we show with red bars positions with SCEs at a 10-codon resolution. Segments for which regions of excess synonymous constraint were not previously reported by Li and colleagues [36] are indicated with asterisks.

Consistent with previous work by Li and colleagues [36], we identify significant regions of excess synonymous constraint in all rotavirus segments (Figure 4). In all segments except for segment 11, the detected regions of excess constraint lie at the beginning or end of the gene. (We recover the overlapping NSP6 gene within the NSP5 ORF in segment 11 as a strong signal of excess synonymous constraint in the interior of the gene).

For three genome segments (NSP4, VP2, and VP6) in which Li and colleagues identify possible RNA structural elements but no signal of excess synonymous constraint [36], we identify strong SCEs across multiple sliding window sizes. Like previously described sites of excess synonymous constraint in rotavirus, the SCEs in NSP4, VP2, and VP6 are concentrated near the beginnings and ends of the respective ORFs, further supporting the biological significance of these additional constrained elements.

### Identifying novel candidate overlapping elements in bluetongue virus

We identify several intriguing signals of excess synonymous constraint in bluetongue virus. Bluetongue virus is a double-stranded RNA virus with 10 genomic segments. It infects ruminants and is a major cause of disease in domestic livestock. We obtained 58 to 248 complete sequences for each bluetongue virus segment from NCBI. The bluetongue virus genome contains a region within the VP6 gene that has been identified as an overlapping gene in the +1 reading frame [37,38].

**Figure 5:**
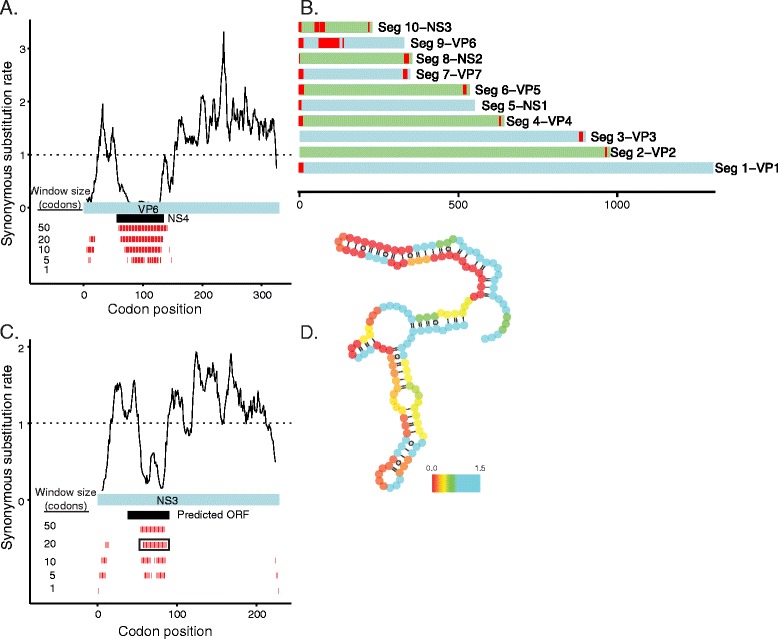
**Identifying putative novel overlapping elements in bluetongue virus. (A)** FRESCo recovers a previously identified overlapping ORF in the VP6 gene as a pronounced region of excess synonymous constraint. **(B)** For each segment of the bluetongue virus genome, we show with red bars positions with SCEs at a 10-codon resolution. As in rotavirus, SCEs are concentrated near the 5’ and 3’ ends of genome segments. **(C)** A conserved ORF in NS3 corresponds to a strong signal of excess synonymous constraint. **(D)** The region also has a weak signal for a conserved RNA structure, suggesting an alternative possible function for the SCE.

We recover several expected signals of synonymous constraint in the bluetongue virus genome. Firstly, we recover the known overlapping gene as a strong region of internal synonymous constraint in VP6 (Figure 5A). In all bluetongue virus segments, we also identify signals of excess synonymous constraint near the 5’ or 3’ termini of the segment (Figure 5B). This is a similar pattern to that observed in rotavirus and may influence packaging, genome replication, or translation as has been hypothesized in rotavirus, also a member of the reovirus family [36].

Additionally, we identify a strong signal of internal synonymous constraint in the NS3 gene on segment 10 (Figure 5C). The internal SCE in NS3 corresponds to a 50- to 59-codon ORF in the +1 reading frame that is conserved across all aligned isolates. Interestingly, for both segment 9, which contains the known overlapping gene, and segment 10, an alternative initiation site is present due to leaky scanning through the initial start codon [39,40]. However, we also note that there are many nonsynonymous substitutions and few synonymous substitutions with respect to the overlapping reading frame, an uncharacteristic signature for a protein-coding gene. An alternative possibility is that this SCE may encode an RNA structural element, since the region also shows a weak signal for the presence of a conserved RNA structure (Figure 5D; Additional file 7).

### Identifying novel regions of excess synonymous constraint with conserved, stable predicted RNA structure

In order to identify possible candidates for RNA structural elements among the SCEs, we scanned all regions of excess synonymous constraint for evidence of conserved, stable RNA structure using RNAz. Below, we highlight a few of the SCEs that also have conserved, stable predicted RNA structures in potato virus Y (PVY), turnip mosaic virus (TuMV), cucumber mosaic virus (CMV), foot-and-mouth disease virus (FMDV), and infectious bursal disease virus (IBDV). While we note that these are only computational predictions of RNA structural elements within SCEs, and would require biological validation, we provide a full list in Additional file 7 as a guide for future work.

PVY and TuMV are positive-sense RNA viruses that each encode a single ORF. Both are members of the potyvirus genus, which includes many plant pathogens affecting economically important crops, such as potatoes, tomatoes, and peppers. We obtained about 150 complete sequences of PVY and over 200 TuMV sequences from the NCBI database. An overlapping gene that is conserved across potyviruses [41] lies within the P3 gene of both PVY and TuMV (Figures 5A,B).

**Figure 6:**
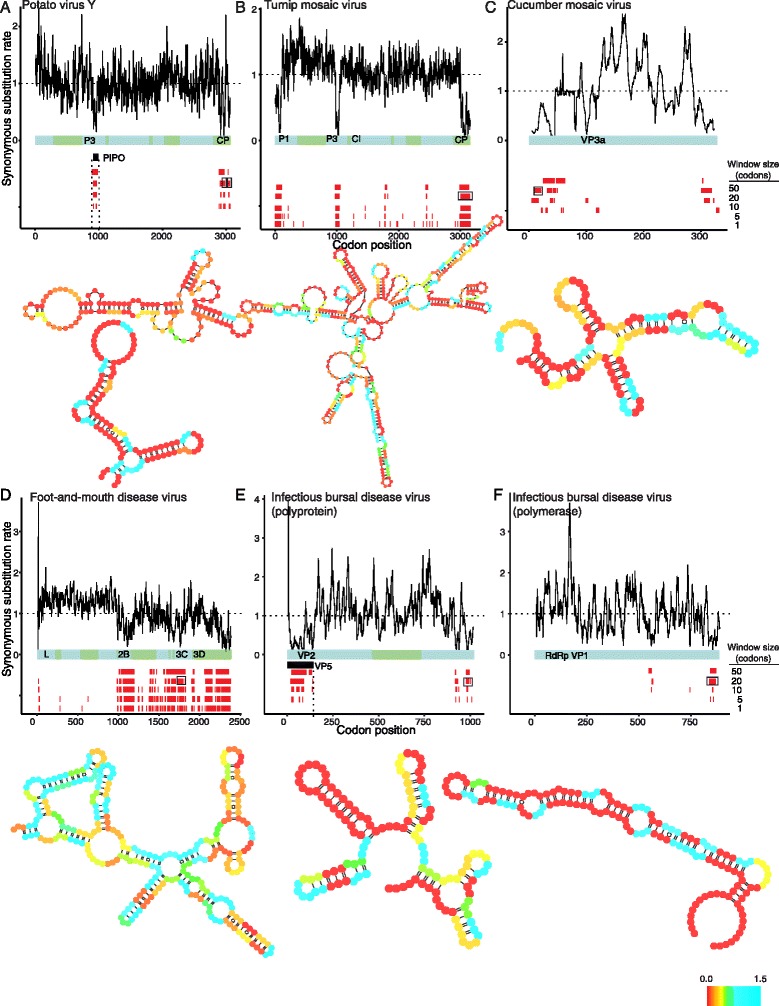
**FRESCo identifies putative novel RNA structural elements in diverse viral genomes.** For each virus, we show a plot of excess synonymous constraint (top) and the putative RNA structure of an SCE (bottom). For each RNA structure, we color base pairs according to the synonymous substitution rate at a single-codon resolution. We highlight with black rectangles the SCEs for which the structure is displayed in **(A)** the potato virus Y polyprotein ORF, **(B)** the turnip mosaic virus polyprotein ORF, **(C)** the cucumber mosaic virus gene VP3a, **(D)** the foot-and-mouth disease virus polyprotein ORF, **(E)** the infectious bursal disease virus polyprotein, **(F)** the infectious bursal disease virus polymerase.

We recover known SCEs as well as predicting novel overlapping elements in PVY and TuMV. In both PVY and TuMV, we identify a signal of excess synonymous constraint that corresponds cleanly to the overlapping reading frame in P3 (Figure 6A,B). In both viruses, we also identify a strong signal of excess synonymous constraint in the capsid gene that does not appear to correspond to a known functional element in either virus. However, an element with RNA secondary structure has been reported in another potyvirus (tobacco etch virus), and mutagenesis studies suggest that this region functions in viral replication [42]. Additionally, a previous computational scan for viral regions with conserved RNA secondary structure [43] also identified an RNA structural element overlapping the potyvirus capsid gene and continuing into the 3’ UTR, further supporting the validity of this putative constrained element. In TuMV, we detect an additional region of strong excess synonymous constraint at the beginning of the P1 gene. This region also has stable, conserved secondary structure detected by RNAz, suggesting that an additional RNA structural element may be present within TuMV P1.

CMV is a positive-sense RNA virus with three genomic segments. It infects an unusually diverse set of hosts, including many crop plants [44]. We obtain over 50 CMV sequences from NCBI for each genomic segment. CMV contains a known overlapping gene in segment 2, which we detect as a pronounced region of excess synonymous constraint. We detect several additional SCEs in CMV, which may correspond to novel functional elements. Several of the SCEs in CMV appear to have stable predicted RNA secondary structures, in particular regions at the beginnings of genes VP2a and VP3a (Figure 6C). These regions represent potential novel functional elements in this important plant pathogen.

FMDV is a member of the picornavirus family and has a single-stranded, positive sense RNA genome with a single ORF. Pathogenic to most cloven-hoofed animals, it is one of the most economically damaging viruses affecting domestic livestock [45]. We compile nearly 400 genomic FMDV sequences from NCBI. Although regions of RNA secondary structure have been identified in the 3’ and 5’ UTRs, there appears to be little previous work studying overlapping functional regions within the FMDV polyprotein ORF. (While many picornaviruses contain a *cis*-regulatory element within their ORF, the FMDV CRE is thought to lie in the 5’ UTR [46]).

Applying FRESCo, we detect multiple regions of excess synonymous constraint in the second half of the FMDV genome (Figure 6D). While a general reduction in synonymous rate observed in the nonstructural relative to the structural genes may be due to a recombination hotspot in FMDV between structural and nonstructural regions [47], a number of sites contain especially strong regions of excess synonymous constraint and are compelling candidates for novel functional elements. (We also recover many of these regions when running our method on the nonstructural genes only, with a phylogeny constructed based on only the nonstructural regions). For example, strong signals of excess synonymous constraint within the 2B, 3C, and 3D genes display stable and conserved RNA secondary structure. The constrained elements with predicted RNA structural elements that we observe in FMDV do not appear to have been previously reported, and our results suggest that overlapping functional elements important for understanding the biology and pathogenesis of FMDV may lie within its nonstructural genes.

IBDV is a double-stranded, bisegmented RNA virus. An important animal agricultural pathogen, it causes disease in young chickens. We compiled over 40 sequences for each IBDV genomic segment from NCBI. The beginning of segment A, which contains the polyprotein and is post-translationally cleaved into multiple mature proteins, overlaps with an additional gene, which we detect as a pronounced region of excess synonymous constraint. The 3’ ends of both the polymerase and the polyprotein ORFs of IBDV form stable, highly conserved predicted secondary structures, and represent candidate novel functional elements (Figures 6E,F). (A region of excess synonymous constraint at the beginning of the polyprotein ORF, where the polyprotein overlaps with the VP5 gene, also corresponds to a stable, conserved RNA structure with multiple stem-loops, suggesting that the RNA structure of the overlapping reading frame in IBDV may be functionally important as well.)

### Identifying novel regions of excess synonymous constraint in Ebola virus and Lassa virus

Ebola virus and Lassa virus are both RNA viruses that cause deadly hemorrhagic disease in humans. Ebola virus is a negative-sense RNA virus with seven genes, while Lassa virus is an ambisense RNA virus with four genes. An outbreak of Ebola virus emerged in Guinea in March 2014, and has since spread through Liberia and Sierra Leone, creating a global threat. Lassa virus is endemic to this region, and is of increasing concern as the high season of Lassa fever approaches amidst the continued Ebola outbreak. We examine data for 124 sequences of viruses in the Ebola genus (including sequences of Bundibugyo ebolavirus, Tai Forest ebolavirus, Ebola virus, Sudan ebolavirus, and Reston virus) and for 95 Lassa virus sequences.

**Figure 7:**
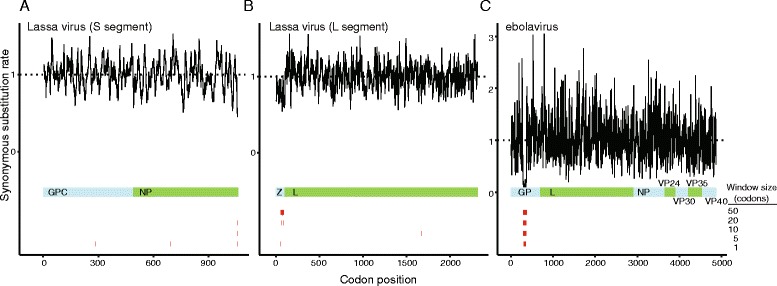
**Regions of excess synonymous constraint in the Lassa virus and Ebola virus genomes. (A)** Lassa virus (S segment). **(B)** Lassa virus (L segment). **(C)** Ebola virus (genes concatenated in alphabetic order).

We applied FRESCo to detect regions of excess synonymous constraint in Lassa and Ebola viruses. In Ebola virus, we identify a single region of excess synonymous constraint corresponding to a known RNA editing site in the GP gene and subsequent overlapping reading frames (Figure 7C) [48]. The significant synonymous constraint following this known editing site suggests that the alternative reading frames in GP are under selective pressure, and that their amino acid sequences are functionally significant. In Lassa virus, we identify two regions of significant excess synonymous constraint, one at the end of the Z gene and one at the end of NP (Figure 7A,B). The functional significance of these regions of excess constraint is unknown. They may correspond to additional RNA secondary structure or interaction sites for RNA-binding proteins. The region of excess synonymous constraint at the end of the NP gene is palindromic, further supporting the idea that this may correspond to a protein-binding site.

## Conclusions

We present a framework, FRESCo, for detecting regions of excess synonymous constraint, and demonstrate its utility both on simulated data and on a diverse set of viral genomes. FRESCo displays high specificity in tests on simulated data. Our approach also recovers known regions of overlapping function in virus genomes at a high - often single-codon - resolution and identifies candidate novel multifunctional regions within the genomes of multiple viruses with diverse genome architectures. Notably, we detect SCEs in bluetongue virus, potato virus Y, turnip mosaic virus, cucumber mosaic virus, infectious bursal disease virus, and foot-and-mouth disease virus that may represent novel overlapping functional elements in these important human, animal, and plant pathogens.

FRESCo represents a powerful and broadly applicable tool for locating overlapping functional regions hidden within protein-coding regions and for developing testable hypotheses about their function. Our approach uses a model-comparison framework to identify regions of excess synonymous constraint, providing a statistically principled test for regions with reduced synonymous variability. We note that its use is not restricted to viral genes and the method can easily be applied to any alignment of protein-coding regions.

The identification of regions of overlapping function in viral genomes is of particular interest for a number of reasons, however. Since viral genomes are highly compact, and tend to have little space outside ORFs, overlapping elements are often found within viral genes. Since many viruses have a high mutation rate, sequenced isolates of the same virus are often substantially different at the nucleotide level, allowing us to identify regions with unusual evolutionary constraint at a high resolution. Methods such as FRESCo, which allow the systematic investigation of the mutational landscape explored by many related viral isolates, are likely to lead to a better understanding of the complex constraints guiding viral evolution.

Furthermore, finding SCEs in viruses has significant implications for drug and vaccine design. Identifying the functional elements in virus genomes is important for identifying potential drug targets. Moreover, attenuating viruses by introducing large numbers of deleterious synonymous mutations represents an intriguing avenue for vaccine development [49]. The method presented in this paper can pinpoint synonymous changes that are evolutionarily avoided and likely to reduce the fitness of the virus. Thus, our framework can help guide targeted synonymous mutation of viral sequences for developing attenuated vaccines as well as facilitate the mapping of novel functional elements overlapping viral genes.

## Materials and methods

We implement FRESCo in the HYPHY batch language [24]. (See Additional file 8 for an expanded description of the codon model utilized.) Briefly, we first fit a maximum-likelihood HKY model of nucleotide evolution to the sequence alignment. Using the parameters from the nucleotide model, we then estimate branch lengths and codon model parameters using a Muse-Gaut 94 type model with an F3x4 estimator of equilibrium codon frequencies. Finally, we run a scanning window across the alignment. For each window, we estimate position-specific synonymous and nonsynonymous substitution rates (alternative model) and nonsynonymous substitution rate only (null model), and perform a likelihood ratio test to compare the two models. Since these models are nested and the alternative model has one additional parameter, the probability that a window is under excess synonymous constraint is approximated by the chi-squared distribution with one degree of freedom. Since each window represents a separate hypothesis, we report windows falling below a conservative *P*-value threshold of 1e-5 as significant (corresponding to a conservative Bonferroni correction for testing windows over the length of a typical viral genome).

We also implemented our simulation framework in the HYPHY batch language. We simulated sequences at varying branch lengths and levels of synonymous constraint using an HKY model of nucleotide evolution and a Muse-Gaut-type codon model with an F3x4 estimator of equilibrium codon frequencies. As an initial illustration of the method output, we generated a single simulated 500-codon long alignment of 1,000 sequences, with the initial 200 codons having synonymous rate s = 0.6, the next 100 codons having s = 1, the next 20 codons having s = 0.2, and the final 180 codons having s = 1. To systematically test the ability of the method to recover SCEs at varying alignment depths, branch lengths, and strength of constraint, we set codon-specific nucleotide frequencies, codon substitution model parameters, and tree topologies for the simulated sequences based on maximum-likelihood estimates from randomly selected sets of 100, 500, and 1,000 HBV sequences. We scaled the branch lengths in the input phylogenetic tree to give total branch lengths of 2, 4, 6, 10, 20, 30, 40, 50, and 100. For each branch length, alignment depth, and synonymous rate, we simulated 250 codons with synonymous rate set to 1 and 50 codons with synonymous rate set to 0.2, 0.4, 0.6, or 0.8 (for a total of 108 300-codon-long simulated alignments). To examine the distribution of *P*-values when there is no signal of excess synonymous constraint, we also generated 20 500-codon-long simulated alignments at each of the three alignment depths (for a total of 30,000 codons) with the synonymous substitution rate set to 1 throughout. After generating simulated sequence data with the given model parameters, we applied FRESCo to the simulated sequences to test its ability to recover the known regions of excess synonymous constraint in the simulated data.

To apply our framework to virus sequence data, we downloaded sets of virus genes from NCBI; our alignments are available in Additional file 4. We use NCBI queries of the form 'virusname[Organism] NOT srcdb_refseq[PROP] NOT cellular organisms[ORGN] AND nuccore genome samespecies[Filter] NOT nuccore genome[filter] NOT gbdiv syn[prop]' to identify publicly available sequences for each virus species. For each species, we downloaded the coding sequences, separated by gene, translated, and aligned the amino acid sequences using the Muscle alignment tool [50]. We then removed any excessively divergent, long, or short genes, used the amino acid alignment as a guide to construct a codon alignment, and built phylogenetic trees using RAxML v.7.2.8 using the GTRGAMMA model of nucleotide evolution [51]. Branch lengths reported in the paper are equal to the sum of the branch distances in the phylogenetic trees, measured in substitutions per site. For each viral gene, we examined the regions of excess synonymous constraint identified by FRESCo at 1, 5, 10, 20, and 50-codon resolution. For each gene, we also extracted the regions of excess synonymous constraint at a 20-codon resolution, merged overlapping windows, and scanned for regions with conserved secondary structure using RNAz v.2.1 [52]. To scan for regions of conserved secondary structure, we first filtered each alignment to six sequences optimized for a mean pairwise identity of approximately 80% and partitioned each region into 120-nucleotide windows using the rnazWindow.pl script. We scanned for secondary structure on both strands, with an SVN RNA-class probability of 0.1 and a dinucleotide background model. We visualized RNA structures using the VARNA tool [53].

## Additional files

Additional file 1:
**FRESCo source code, instructions for running the method, and sample input files.**
Additional file 2:
**Table of virus genes analyzed.**
Additional file 3:
**Table of regions of excess synonymous constraint in viruses (Excel spreadsheet).** Sequence coordinates are specified relative to the corresponding alignment. Additional file 4:
**Sequence alignments (zip folder of FASTA alignments).**
Additional file 5:
**Plots of regions of excess synonymous constraint across viruses examined.**
Additional file 6:
**Table of known elements recovered by FRESCo and putative novel elements identified.**
Additional file 7:
**Table of conserved RNA secondary structure predictions in regions of excess synonymous constraint.**
Additional file 8:
**Expanded description of FRESCo codon model.**

## Abbreviations

CMV: cucumber mosaic virus
cHP: capsid-coding region hairpin element
FMDV: foot-and-mouth disease virus
HBV: hepatitis B virus
IBDV: infectious bursal disease virus
ORF: open reading frame
PVY: potato virus Y
SCE: synonymous constraint element
TuMV: turnip mosaic virus
UTR: untranslated region
WNV: West Nile virus
