# Supplementary material for: FRESCo: finding regions of excess synonymous constraint in diverse viruses
Source: Genome Biol. 2015 Feb 17;16(1):38. doi: 10.1186/s13059-015-0603-7 (PMC4376164; doi:10.1186/s13059-015-0603-7)
Supplement: Additional file 5: — Plots of regions of excess synonymous constraint across viruses examined. [file 13059_2015_603_MOESM5_ESM.pdf]

Bluetongue virus VP1

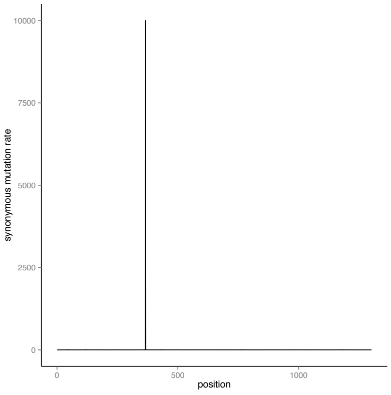

w=1

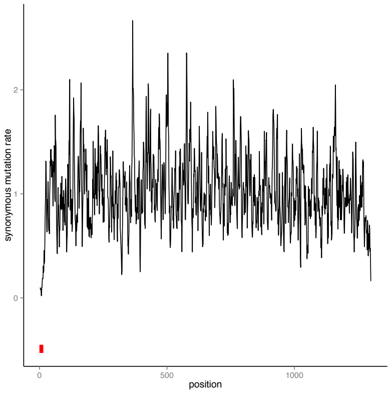

w=5

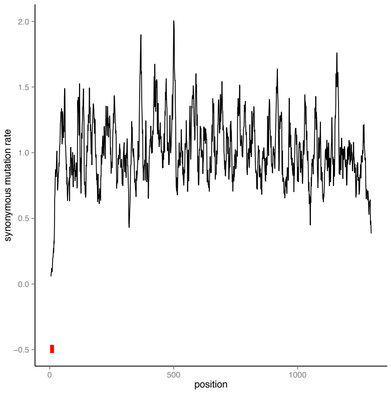

w=10

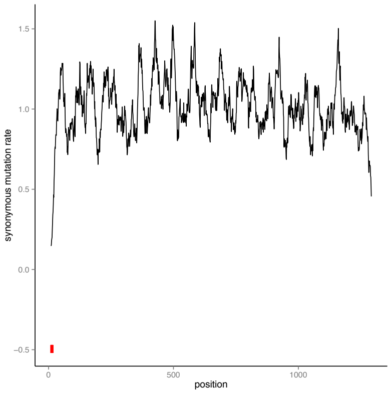

w=20

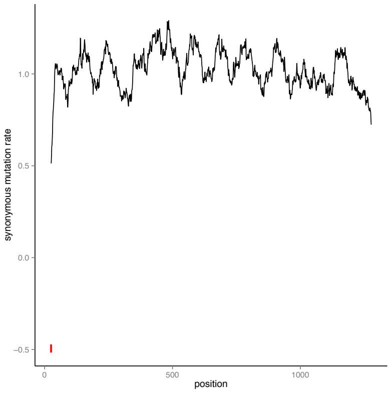

w=50

Bluetongue virus VP2

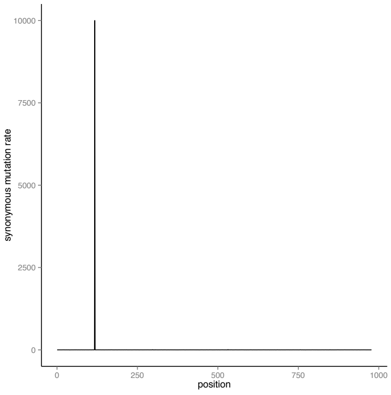

w=1

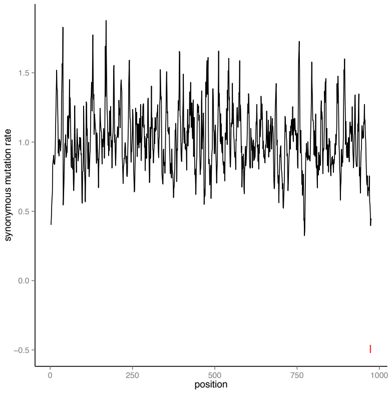

w=5

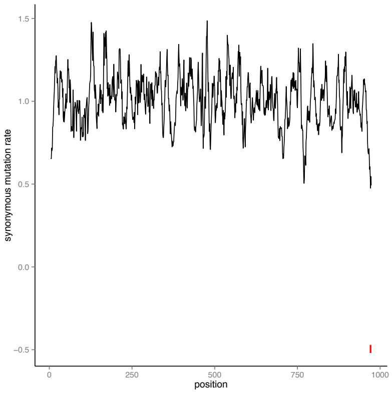

w=10

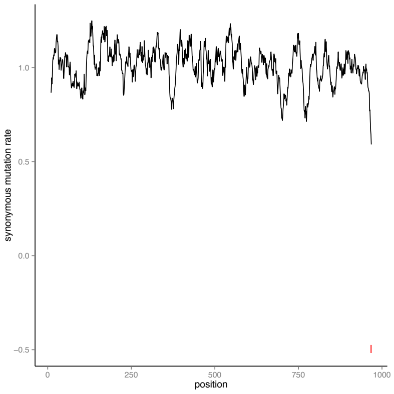

w=20

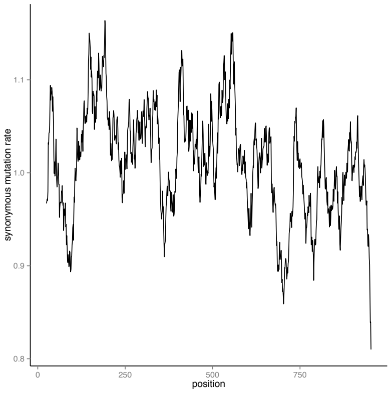

w=50

Bluetongue virus VP3

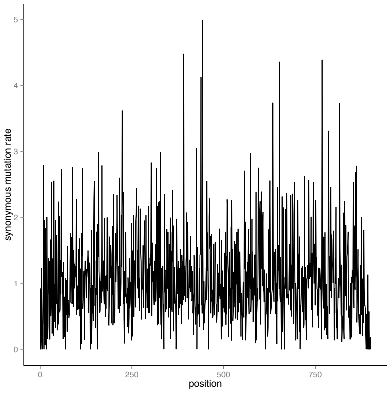

w=1

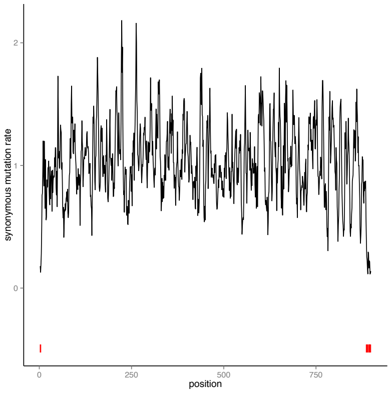

w=5

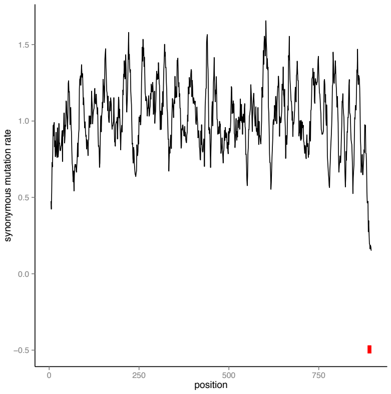

w=10

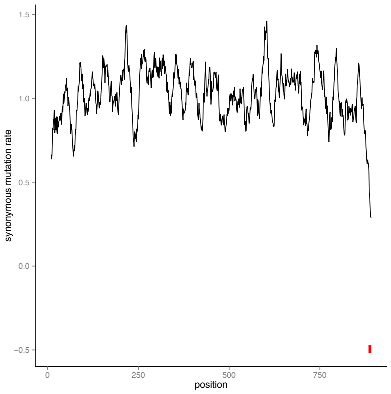

w=20

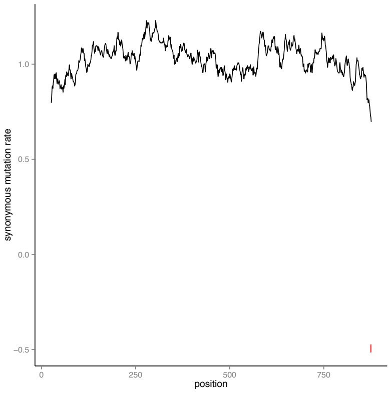

w=50

Bluetongue virus VP4

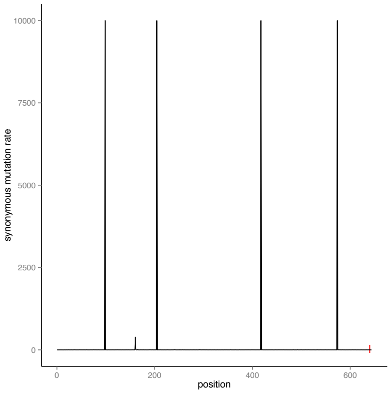

w=1

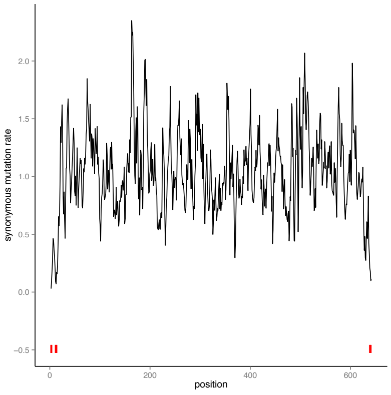

w=5

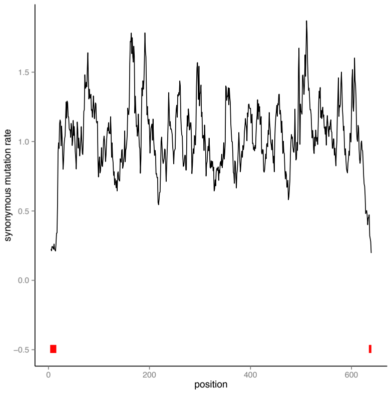

w=10

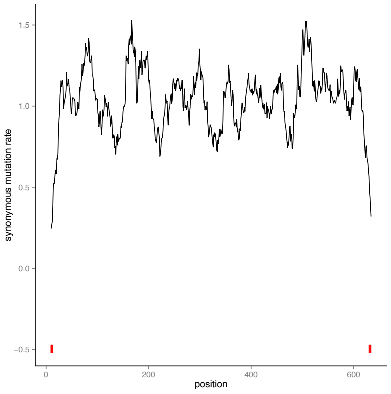

w=20

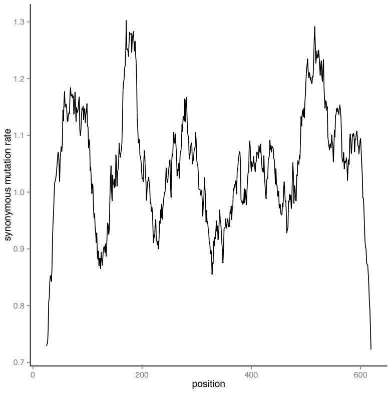

w=50

Bluetongue virus VP5

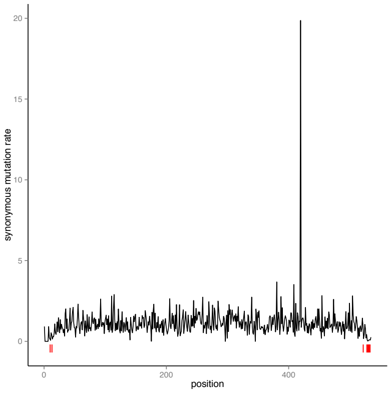

w=1

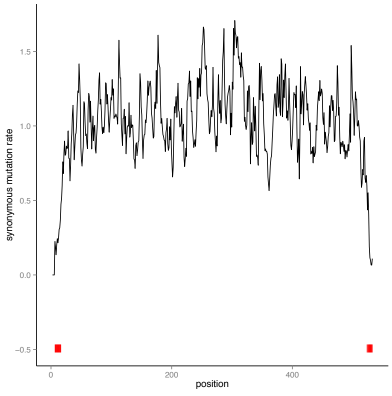

w=5

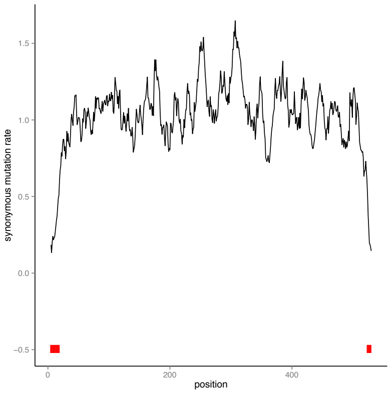

w=10

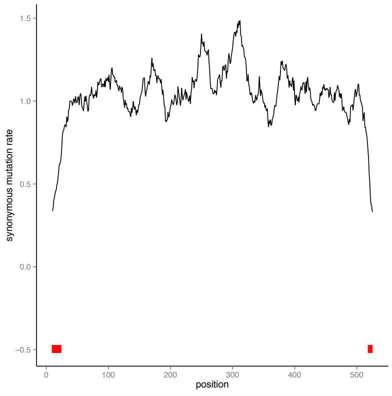

w=20

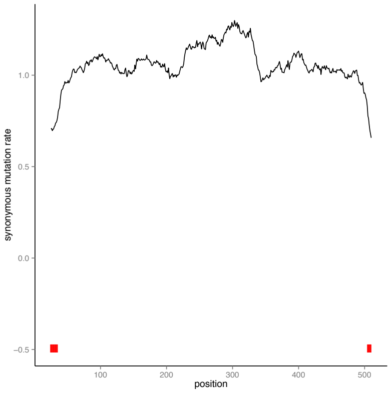

w=50

Bluetongue virus VP6

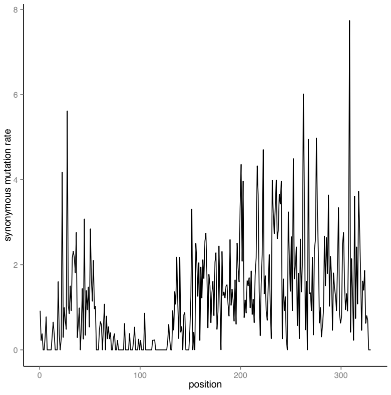

w=1

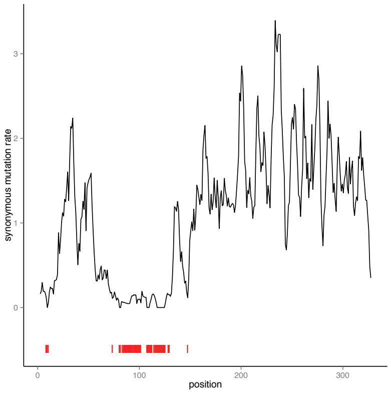

w=5

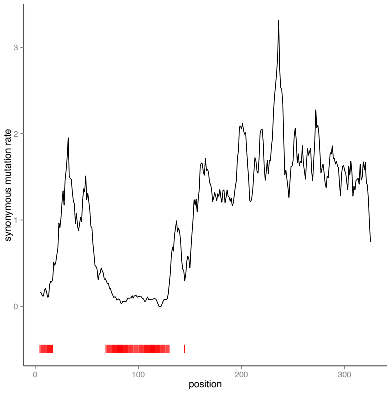

w=10

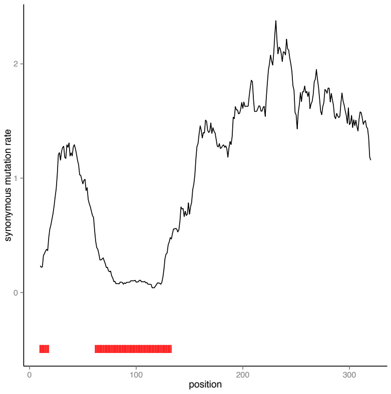

w=20

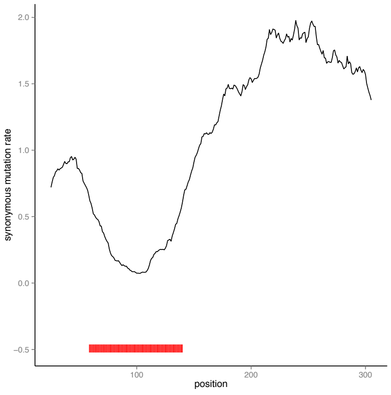

w=50

Bluetongue virus VP7

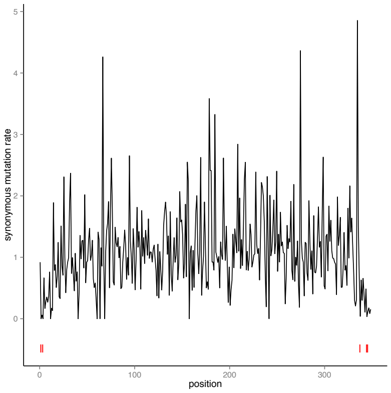

w=1

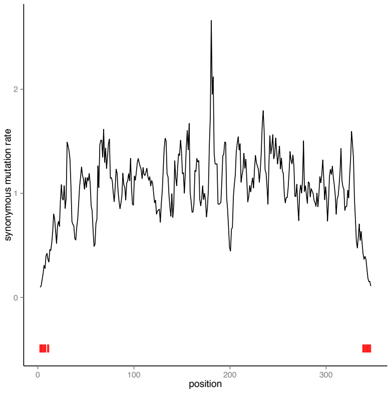

w=5

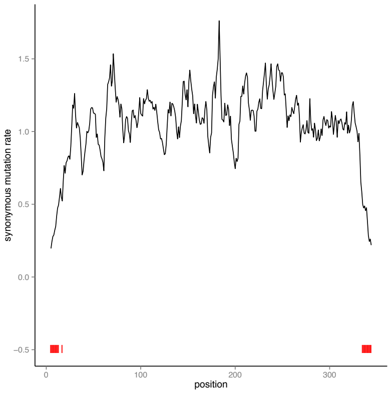

w=10

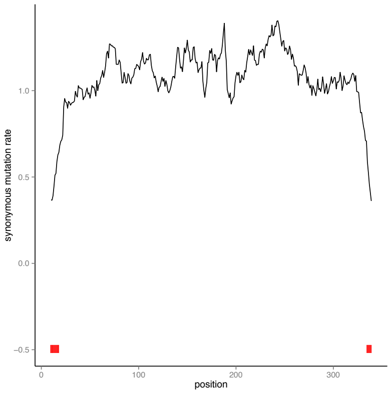

w=20

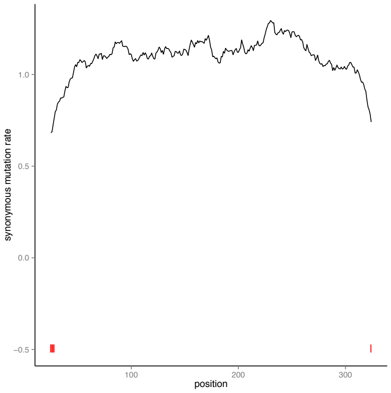

w=50

Bluetongue virus NS1

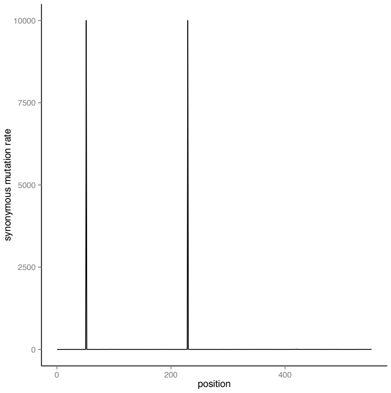

w=1

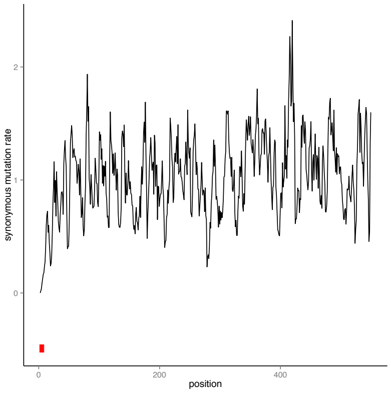

w=5

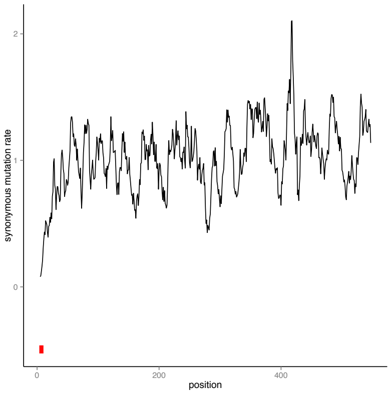

w=10

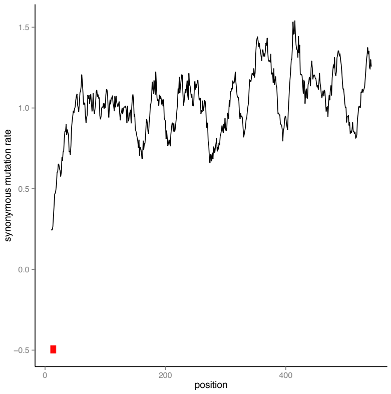

w=20

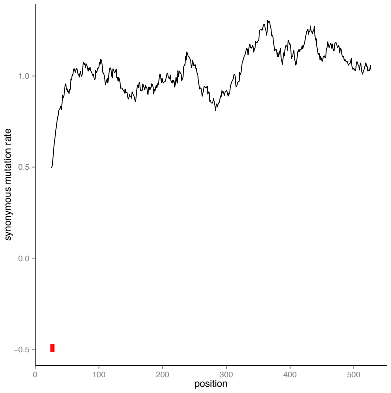

w=50

Bluetongue virus NS2

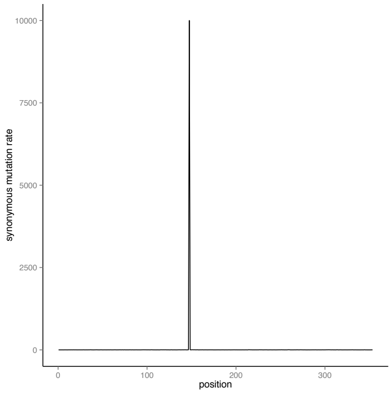

w=1

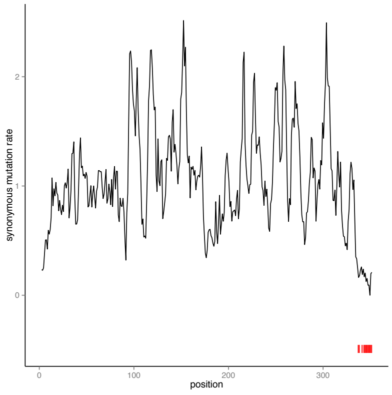

w=5

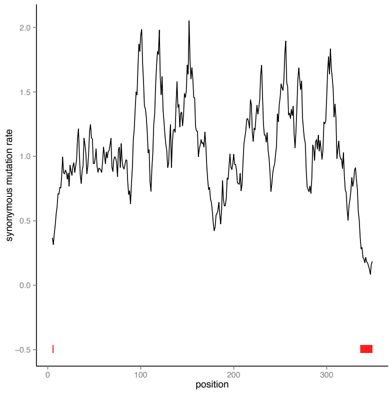

w=10

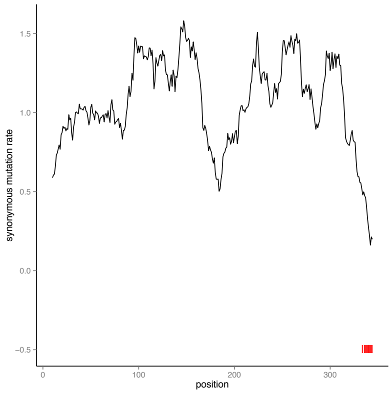

w=20

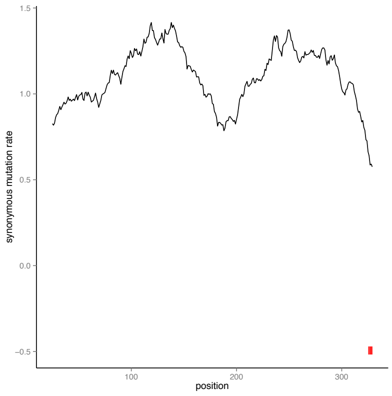

w=50

Bluetongue virus NS3

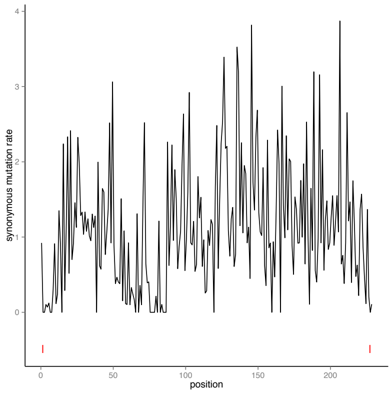

w=1

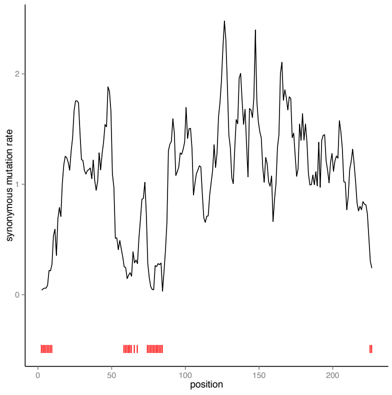

w=5

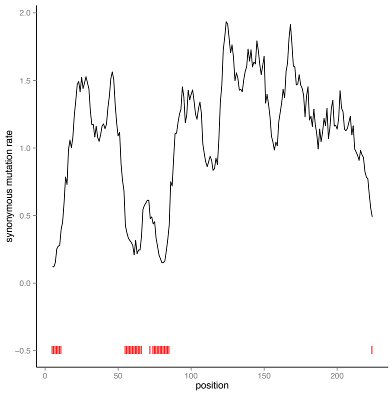

w=10

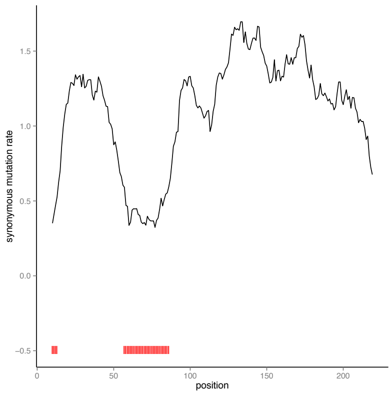

w=20

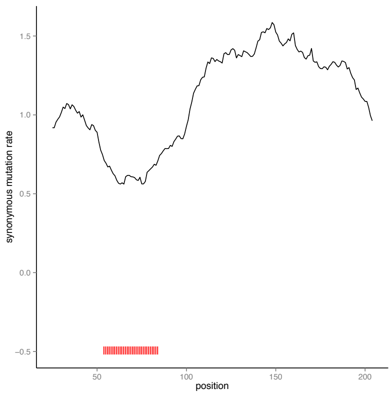

w=50

bocavirus ns

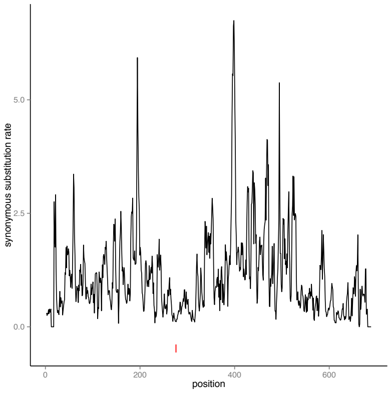

w=5

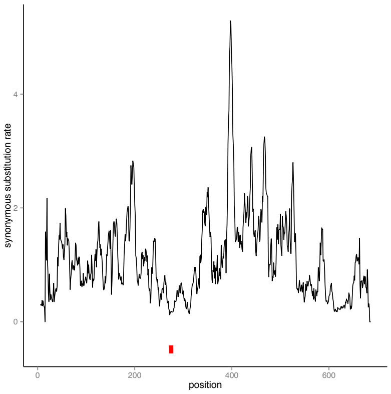

w=10

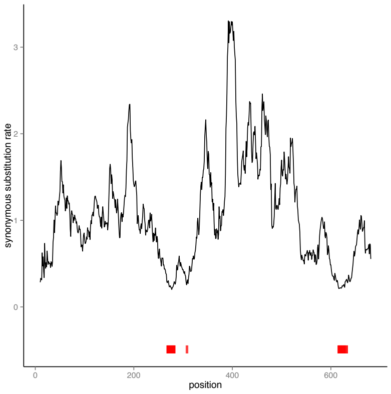

w=20

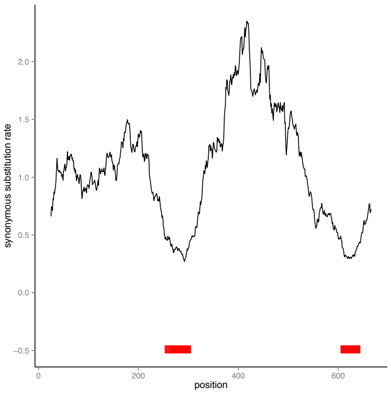

w=50

bocavirus structural

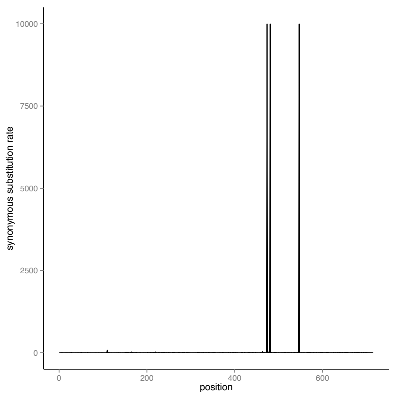

w=1

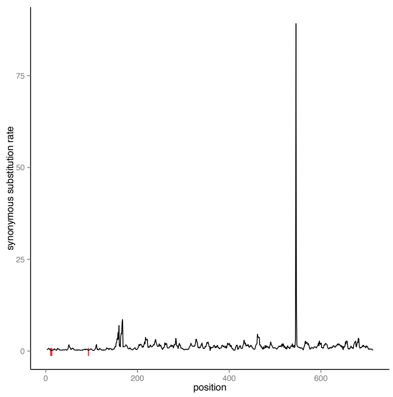

w=5

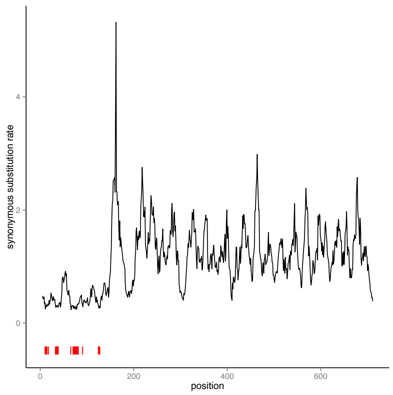

w=10

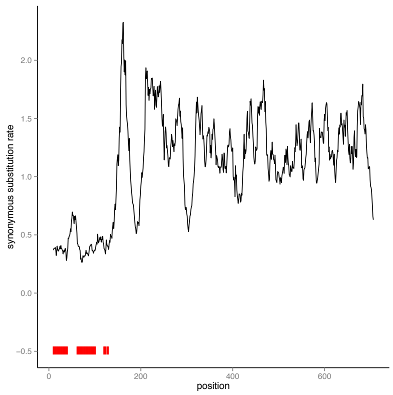

w=20

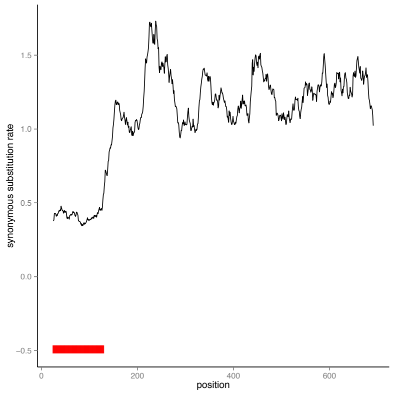

w=50

CCHV glycoprotein

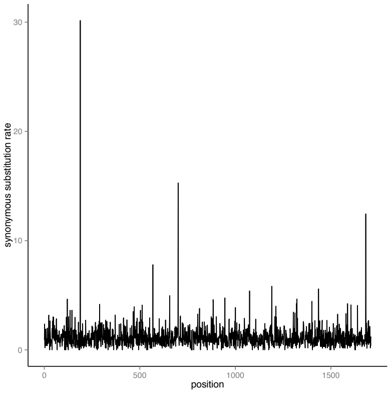

w=1

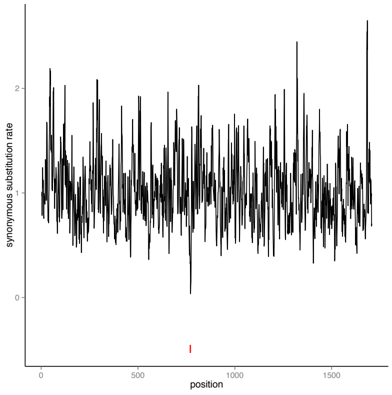

w=5

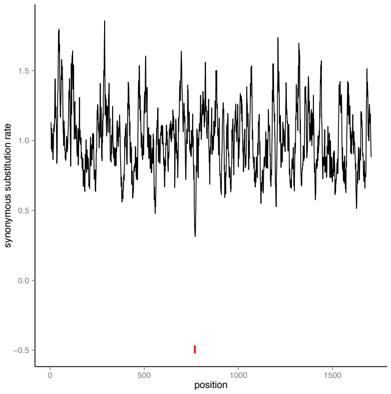

w=10

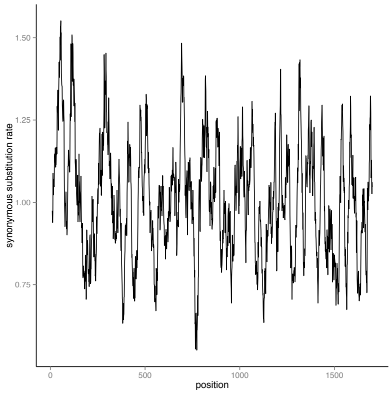

w=20

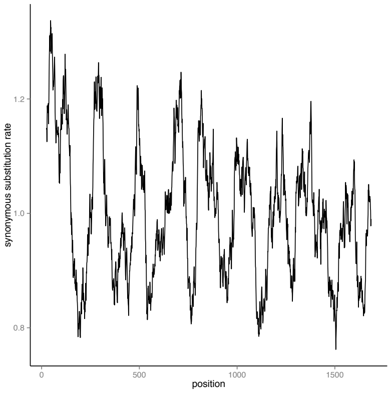

w=50

CCHV nucleoprotein

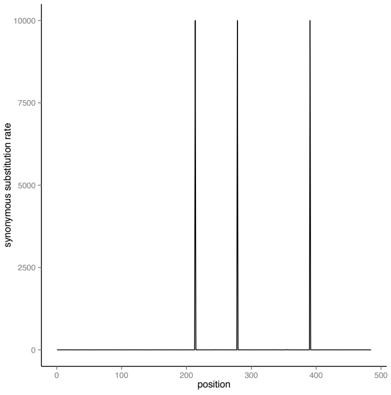

w=1

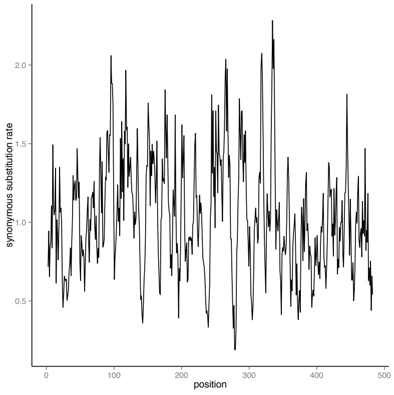

w=5

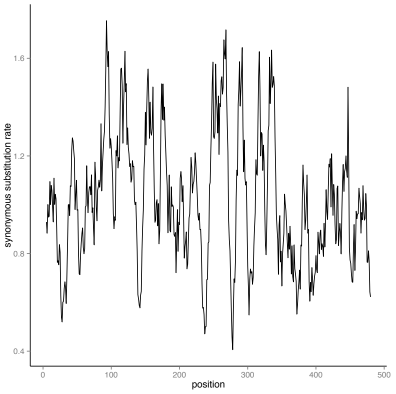

w=10

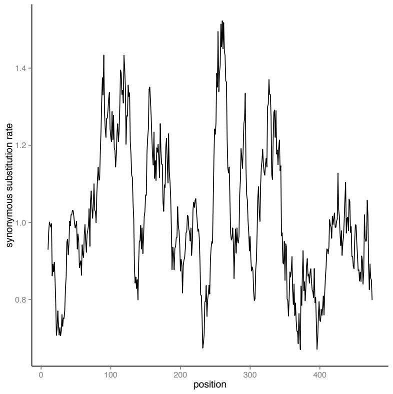

w=20

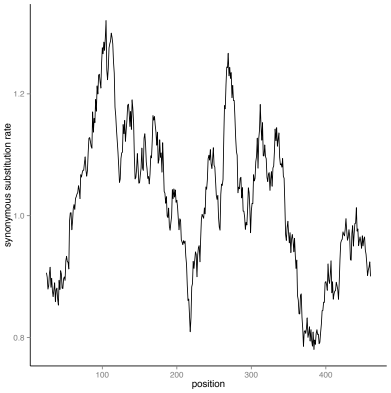

w=50

CCHV polymerase

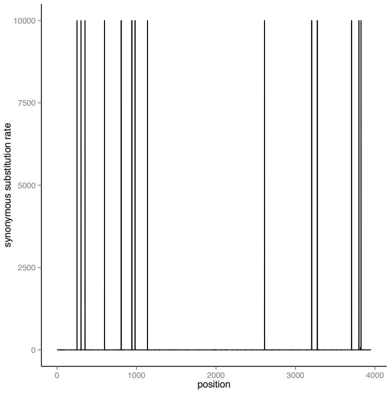

w=1

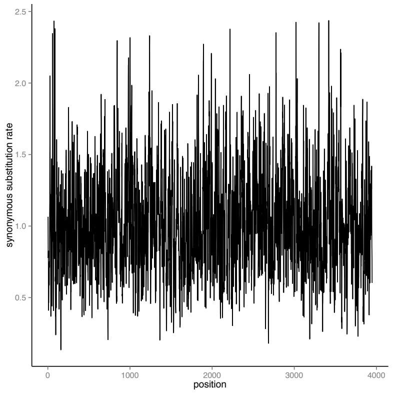

w=5

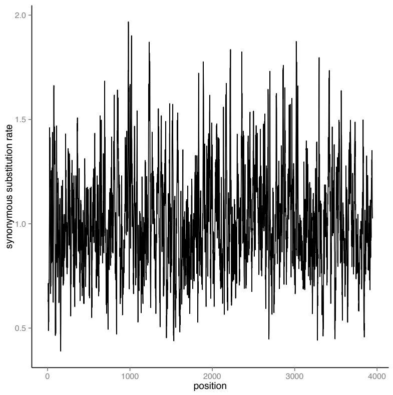

w=10

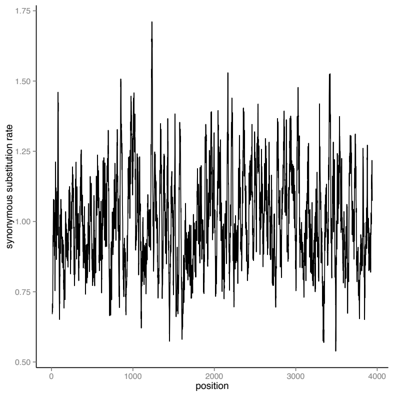

w=20

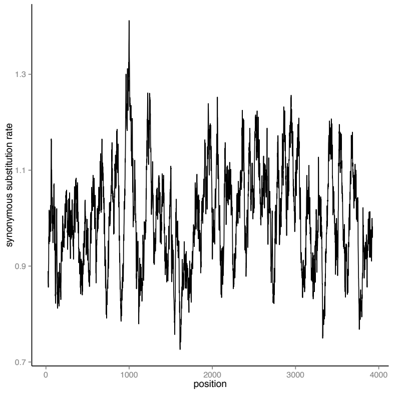

w=50

chikungunya ns

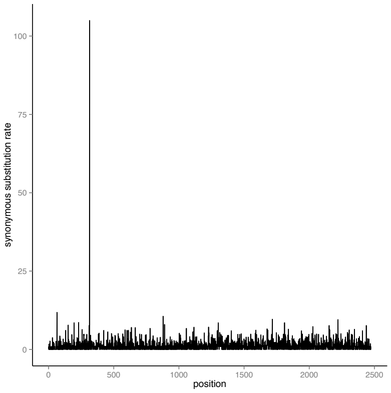

w=1

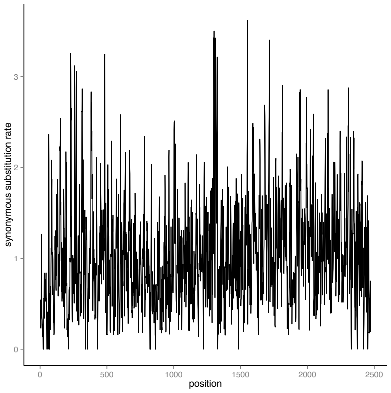

w=5

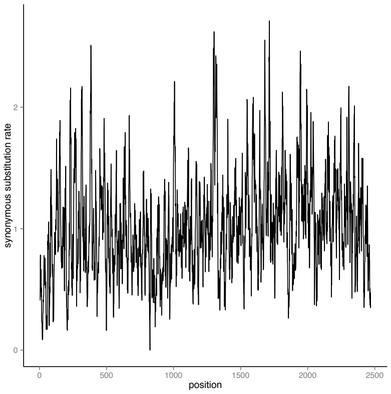

w=10

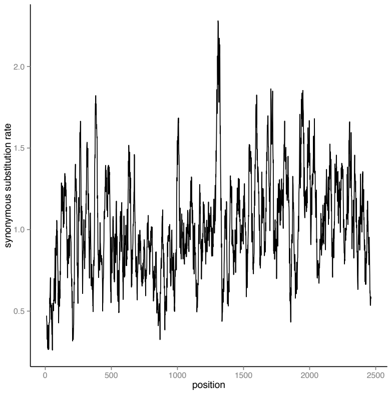

w=20

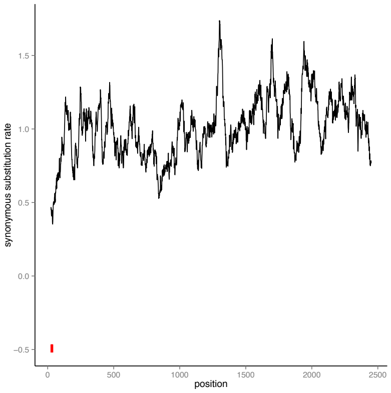

w=50

chikungunya structural

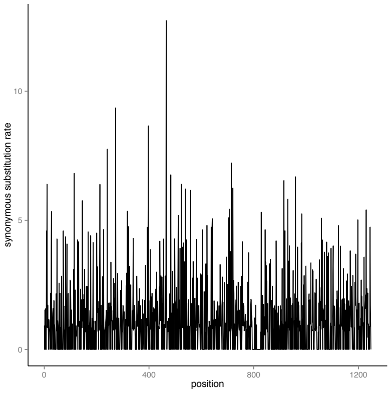

w=1

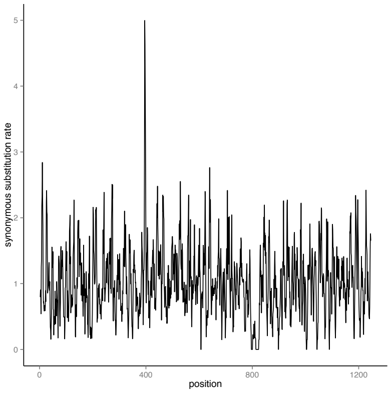

w=5

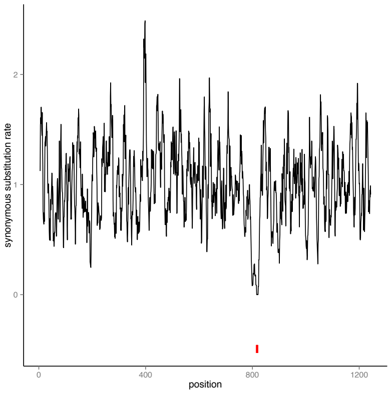

w=10

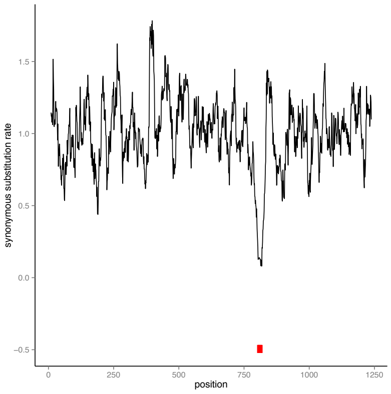

w=20

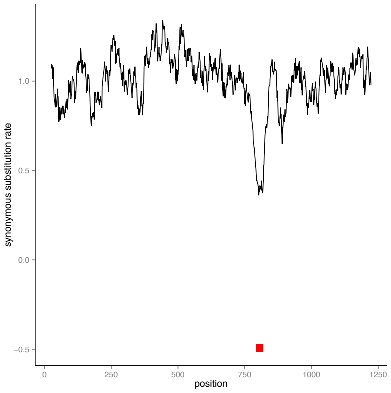

w=50

CucumberMV VP1a

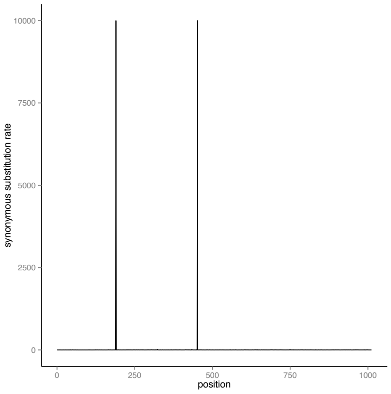

w=1

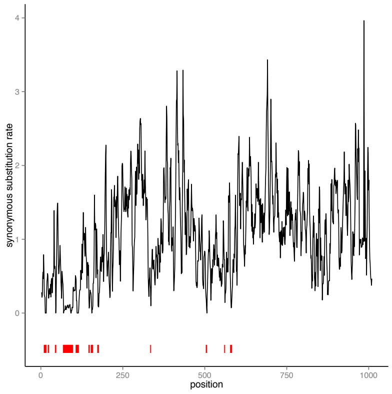

w=5

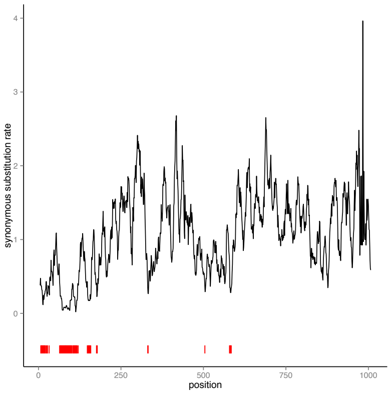

w=10

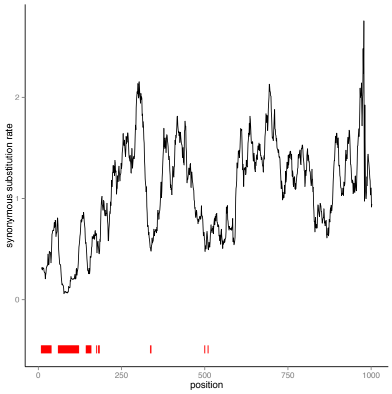

w=20

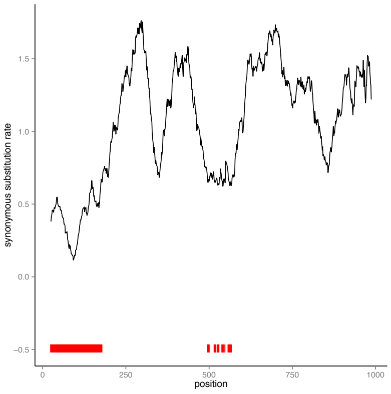

w=50

CucumberMV VP2a

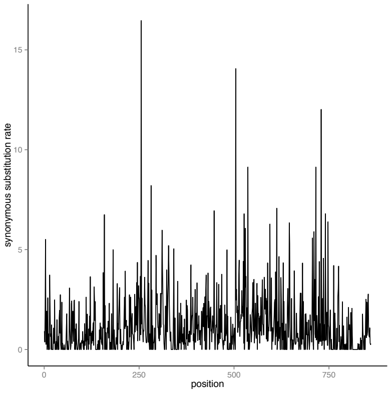

w=1

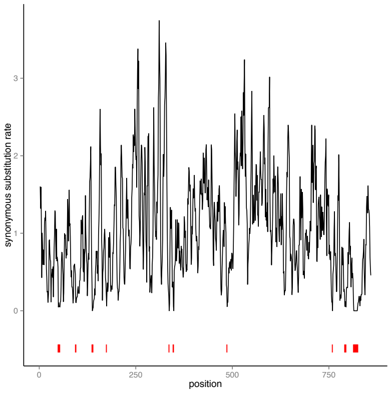

w=5

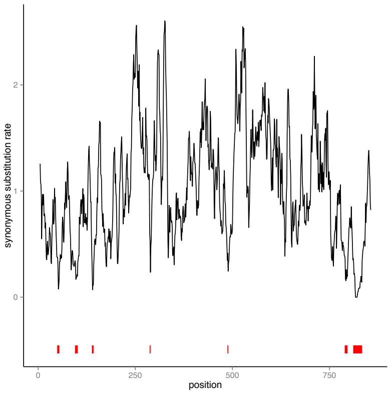

w=10

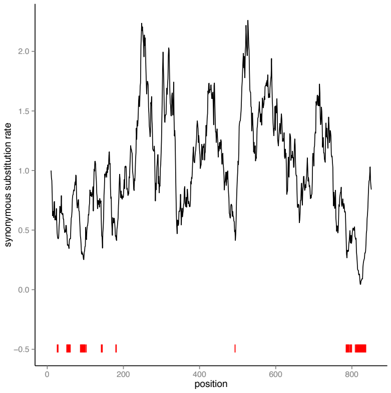

w=20

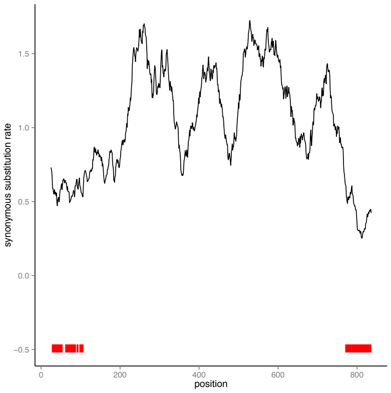

w=50

CucumberMV VP2b

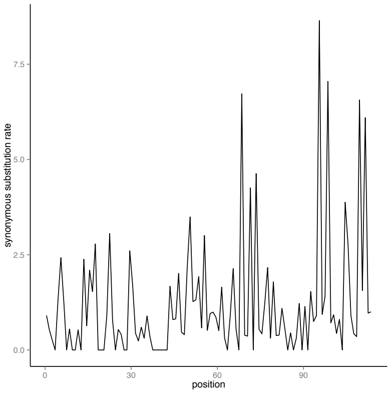

w=1

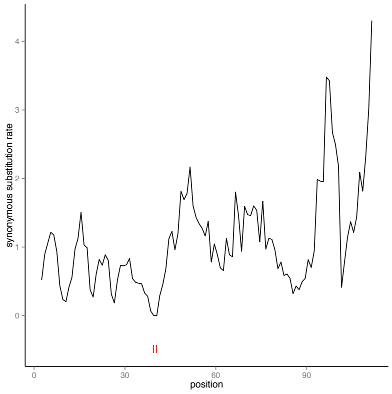

w=5

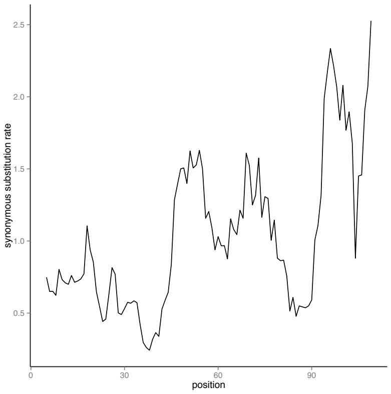

w=10

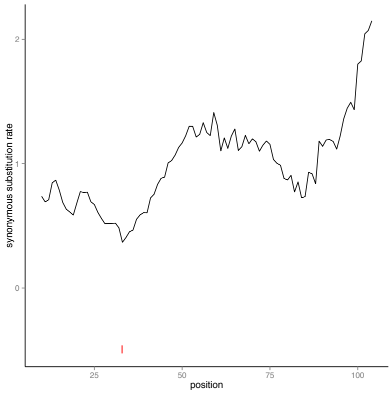

w=20

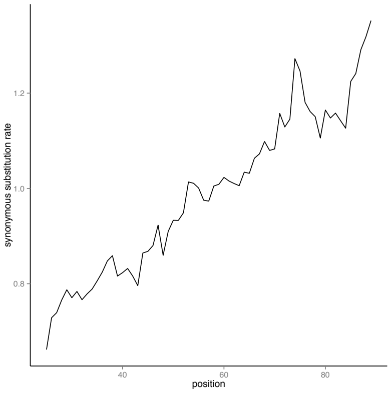

w=50

CucumberMV VP3a

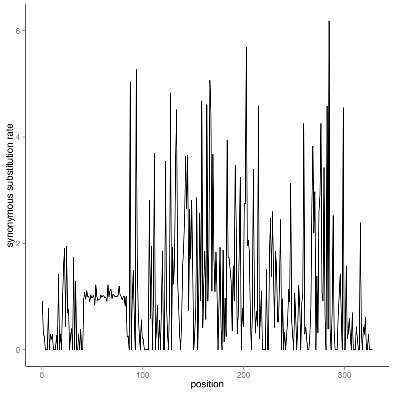

w=1

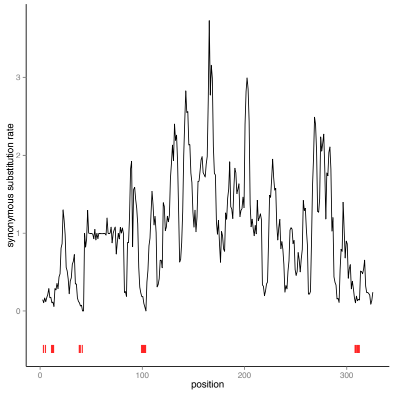

w=5

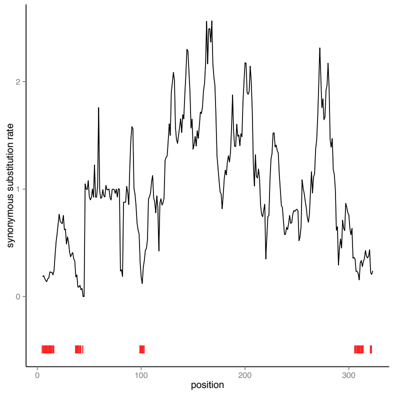

w=10

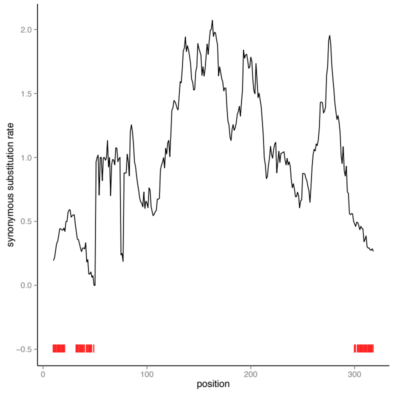

w=20

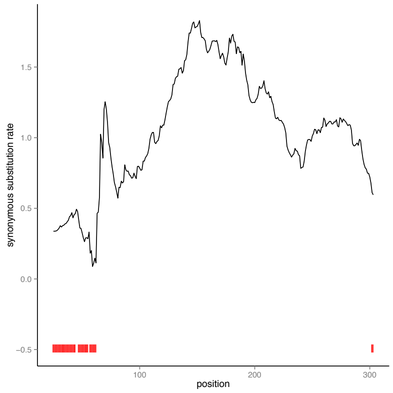

w=50

CucumberMV VP3b

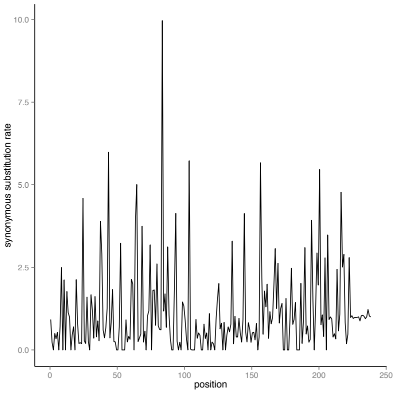

w=1

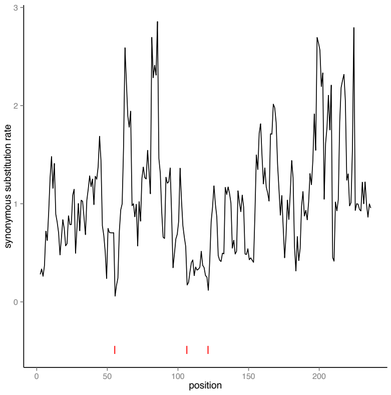

w=5

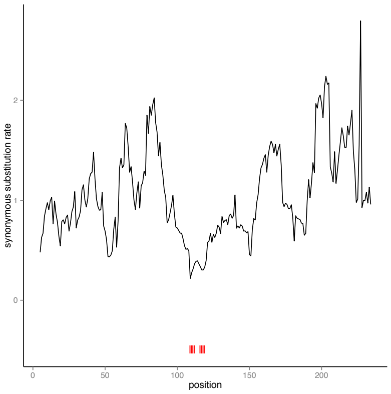

w=10

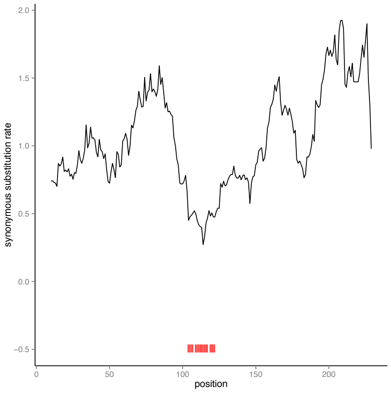

w=20

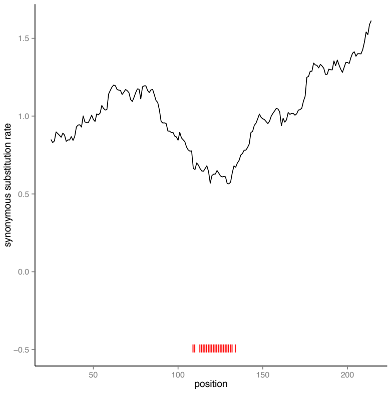

w=50

DENV1

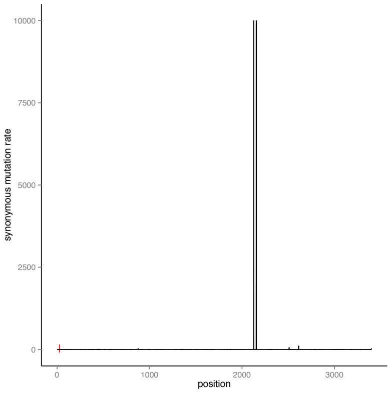

w=1

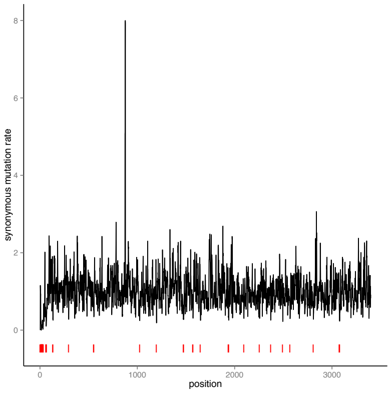

w=5

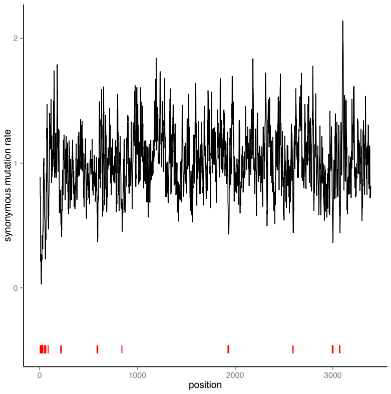

w=10

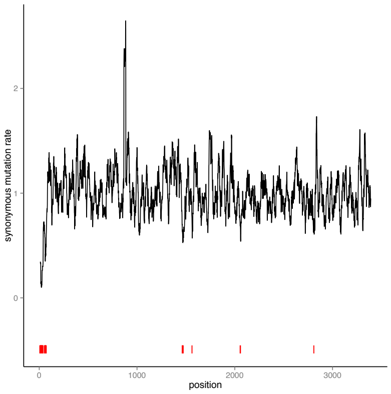

w=20

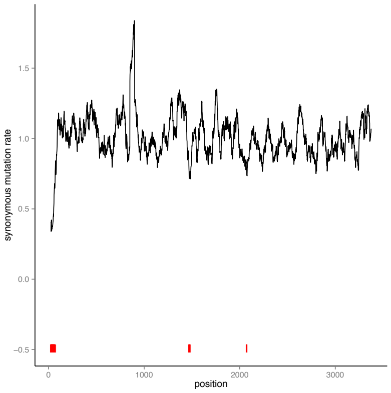

w=50

DENV2

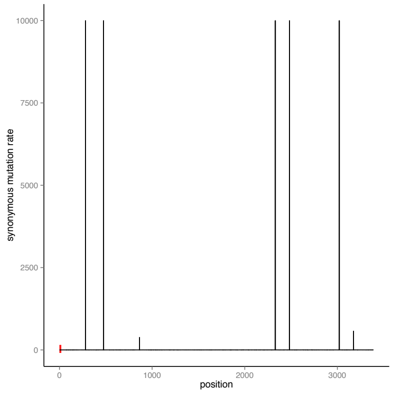

w=1

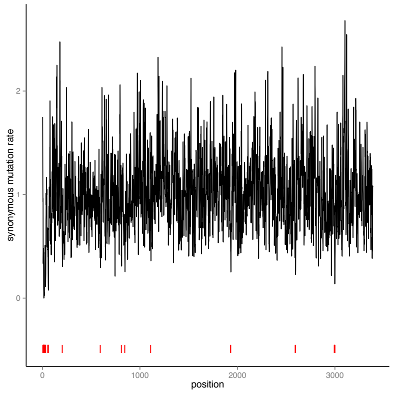

w=5

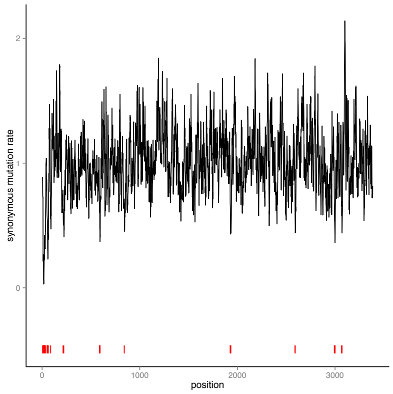

w=10

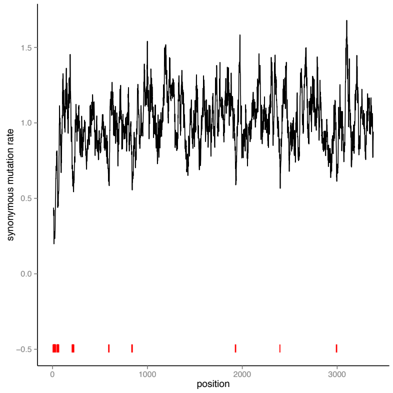

w=20

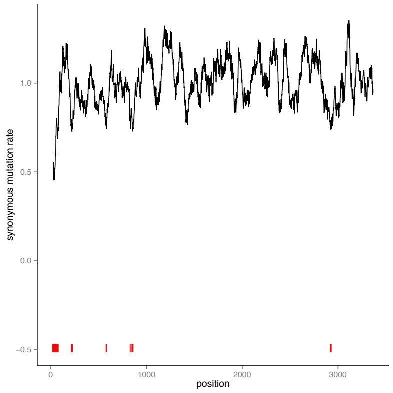

w=50

DENV3

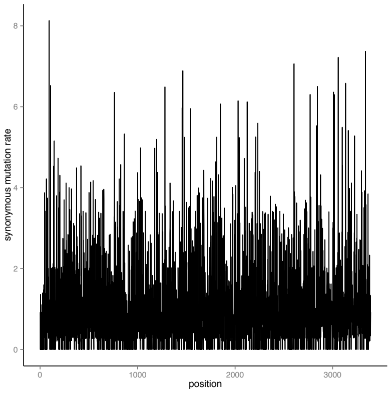

w=1

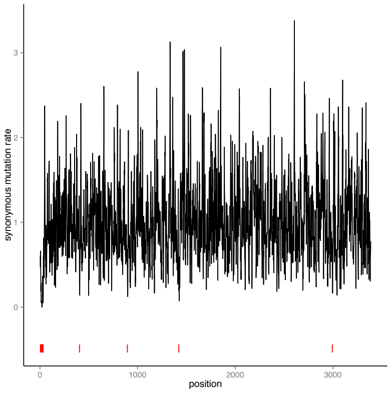

w=5

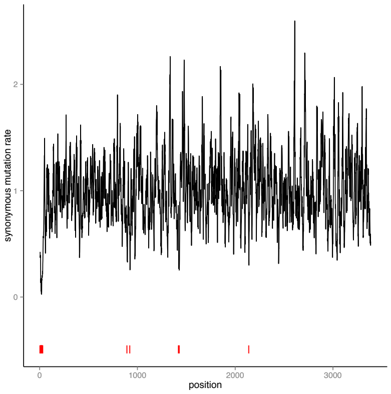

w=10

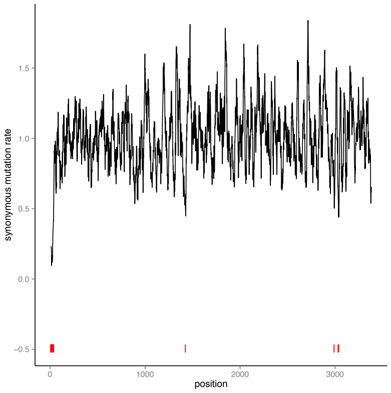

w=20

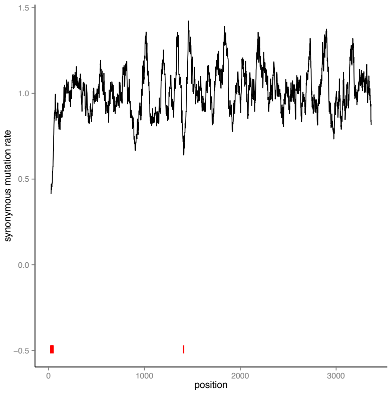

w=50

DENV4

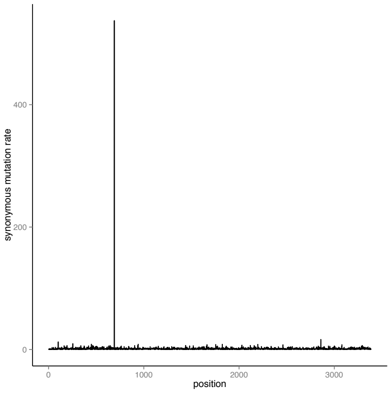

w=1

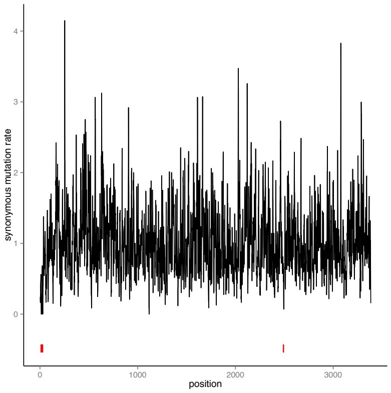

w=5

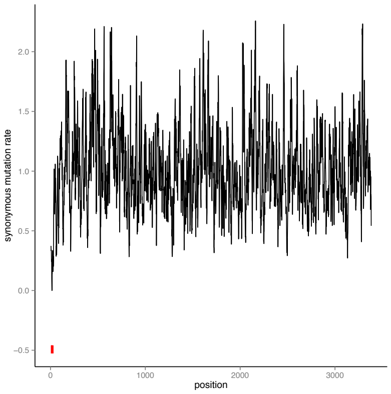

w=10

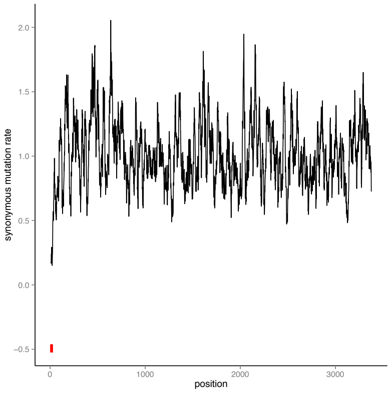

w=20

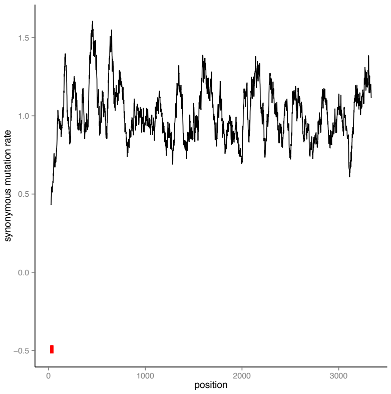

w=50

Enterovirus a71

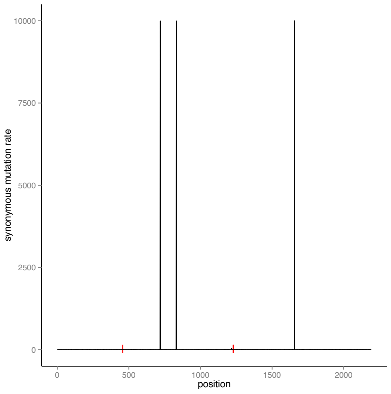

w=1

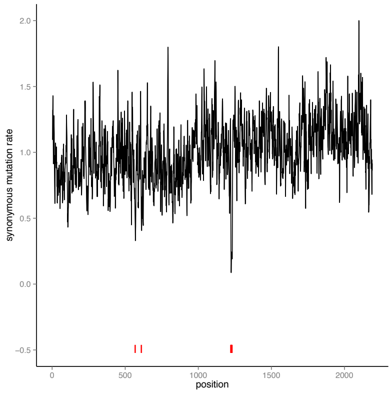

w=5

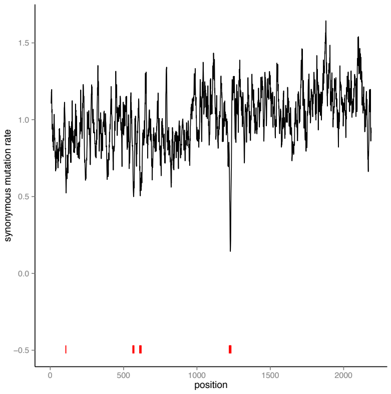

w=10

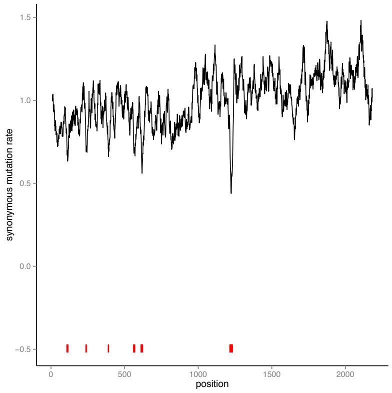

w=20

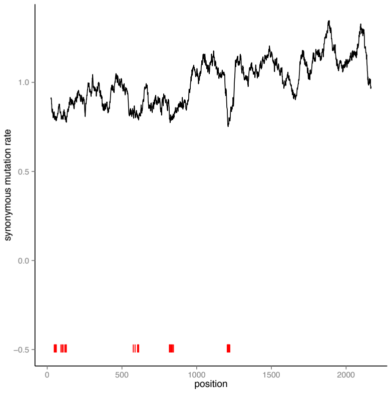

w=50

FMDV polyprotein

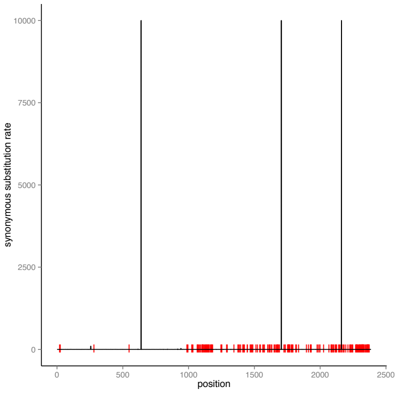

w=1

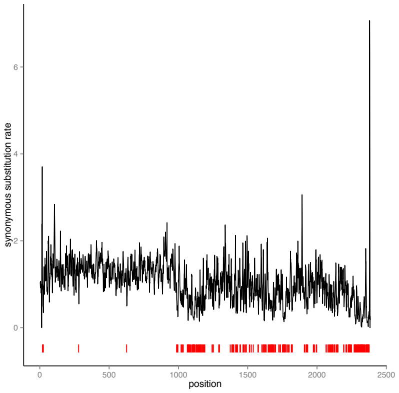

w=5

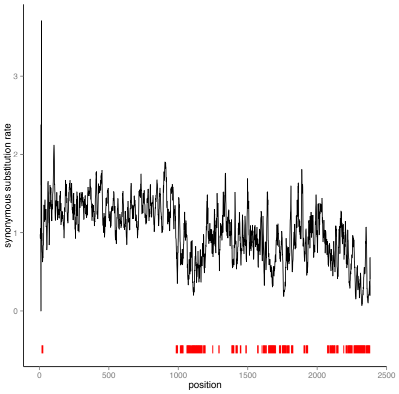

w=10

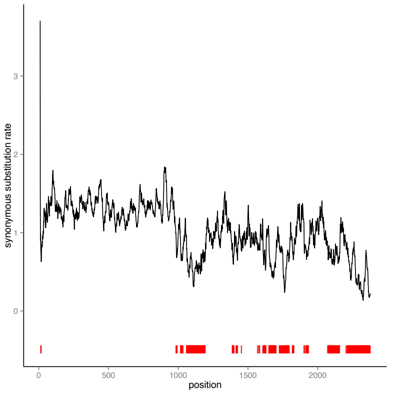

w=20

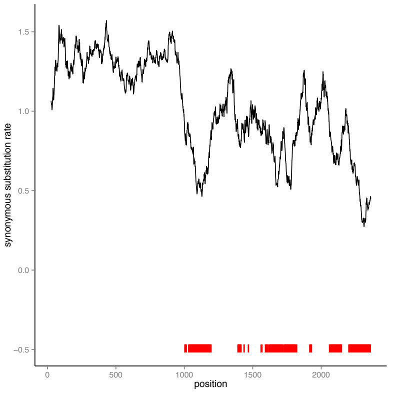

w=50

HBV P

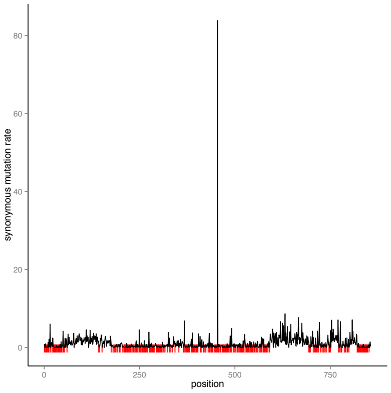

w=1

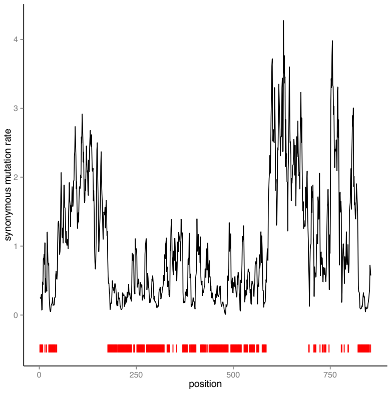

w=5

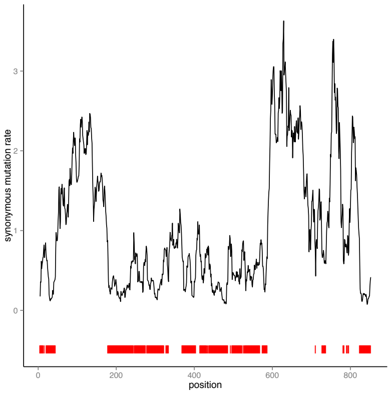

w=10

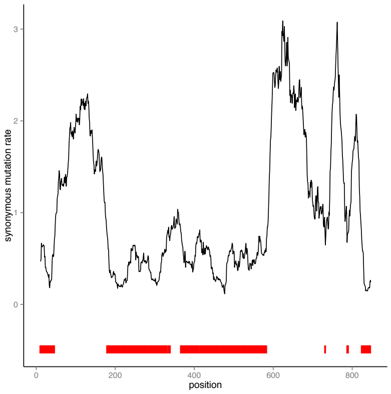

w=20

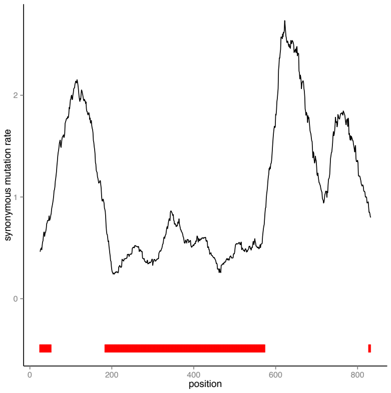

w=50

HCV1a

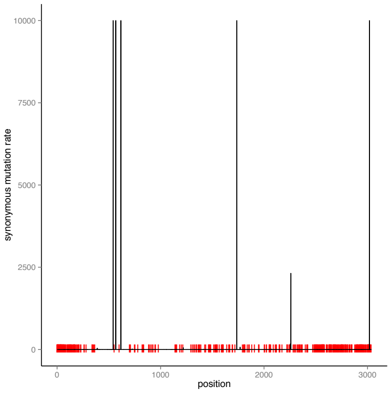

w=1

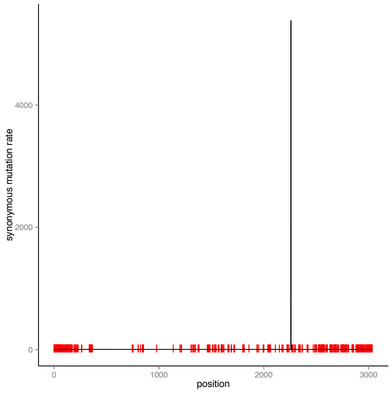

w=5

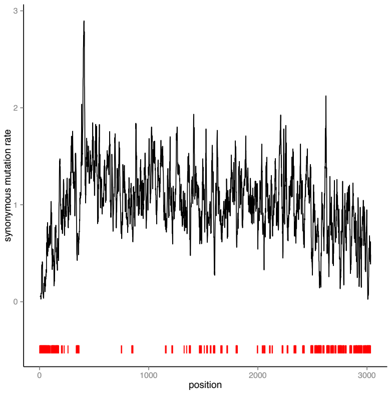

w=10

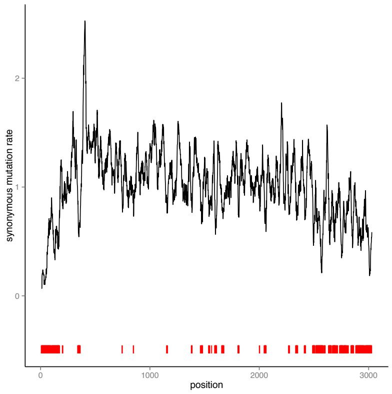

w=20

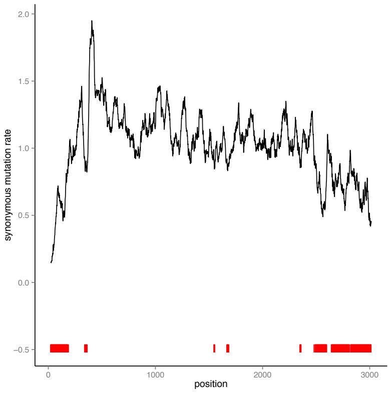

w=50

HCV1b

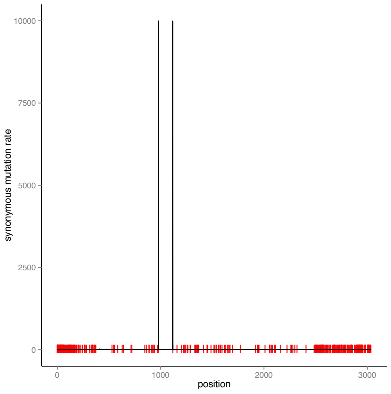

w=1

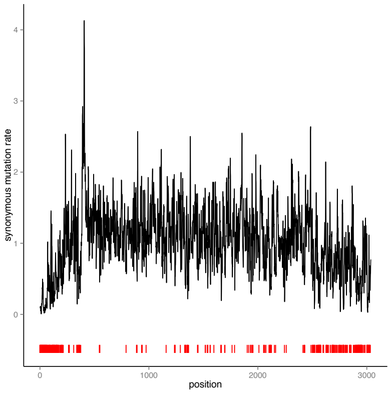

w=5

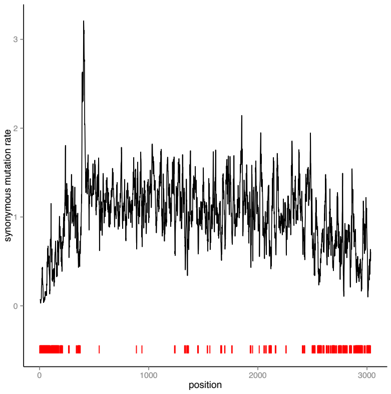

w=10

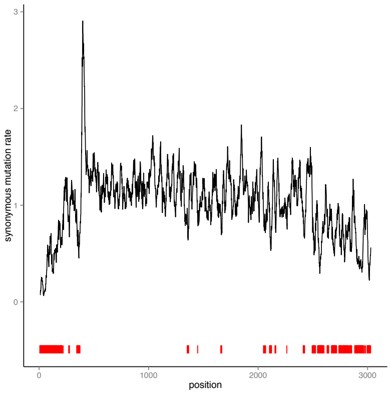

w=20

w=50

HepA polyprotein

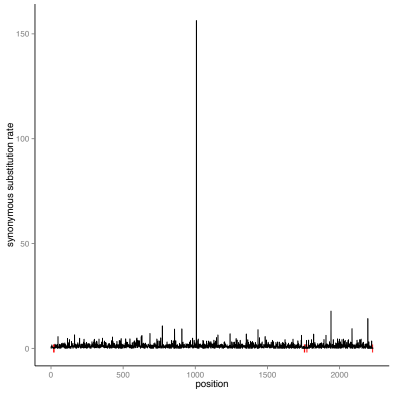

w=1

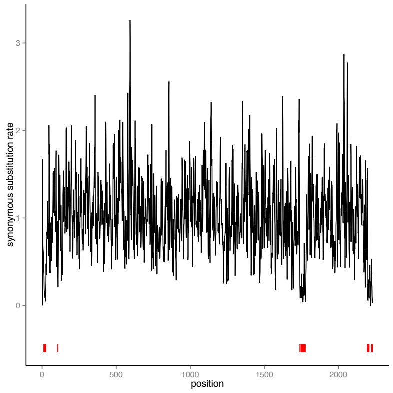

w=5

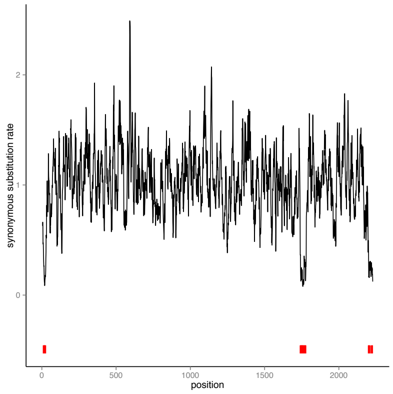

w=10

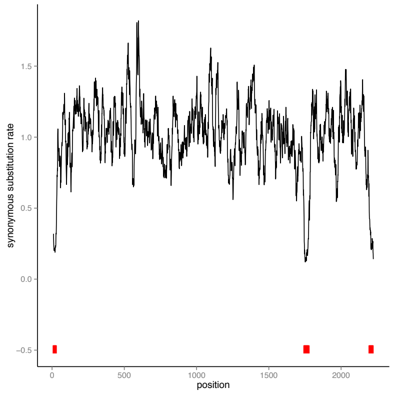

w=20

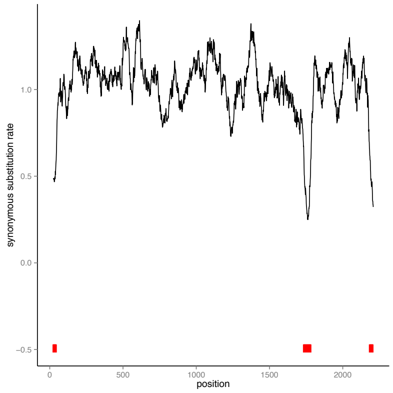

w=50

HepE ORF1

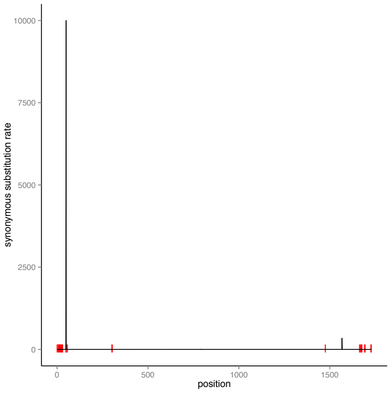

w=1

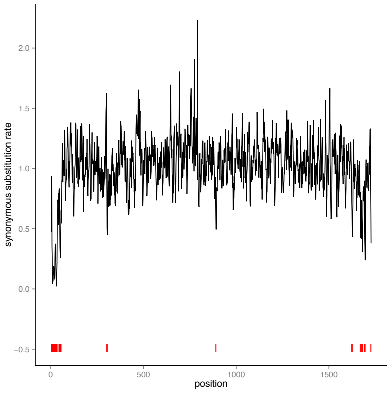

w=5

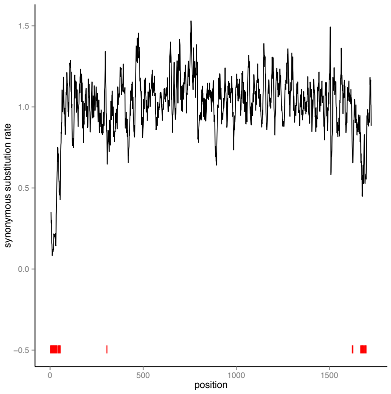

w=10

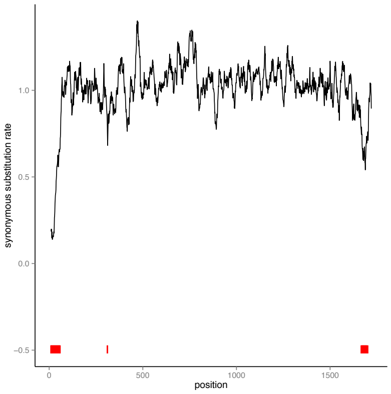

w=20

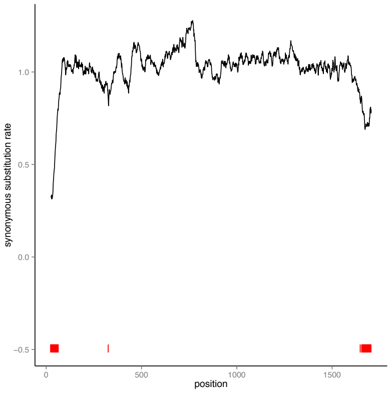

w=50

HepE ORF2

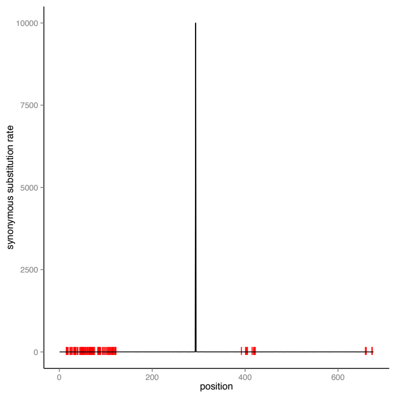

w=1

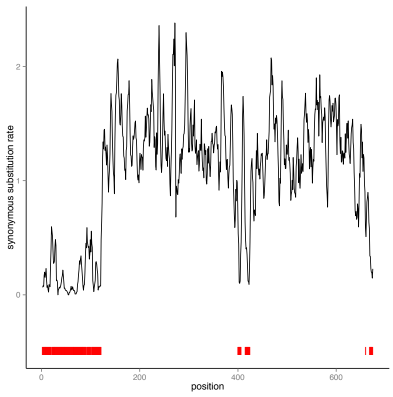

w=5

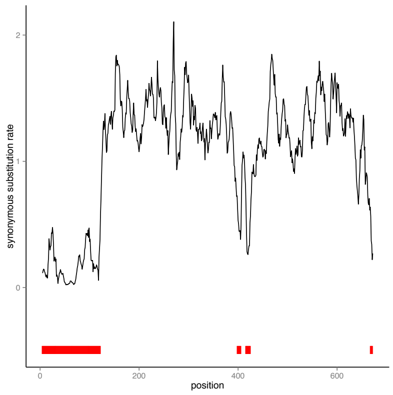

w=10

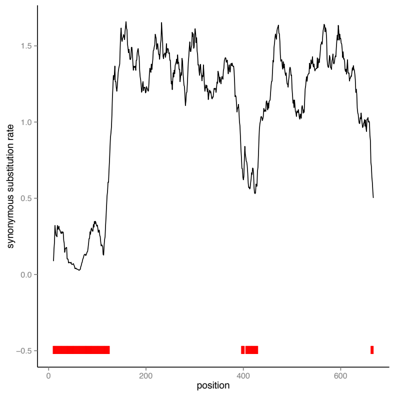

w=20

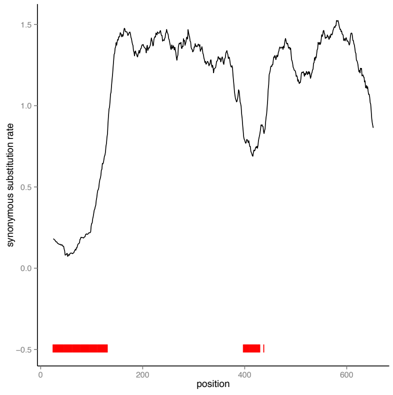

w=50

HepE ORF3

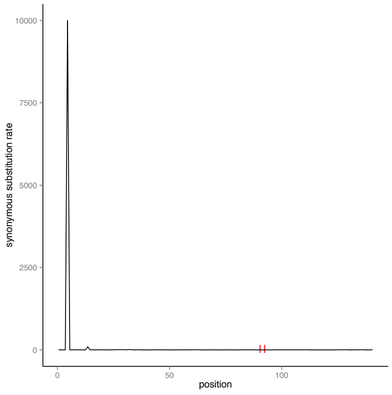

w=1

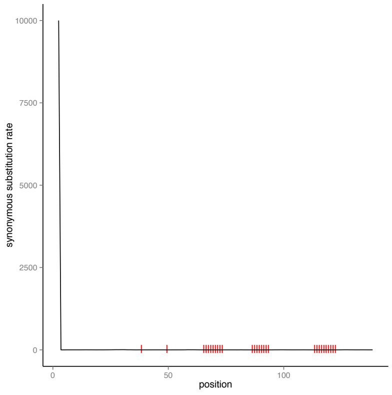

w=5

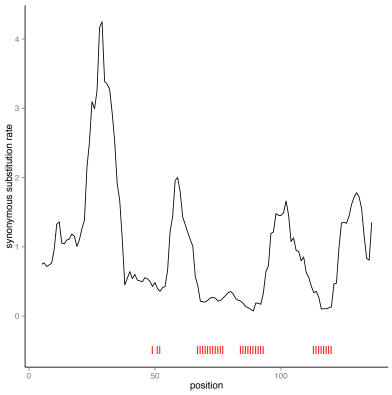

w=10

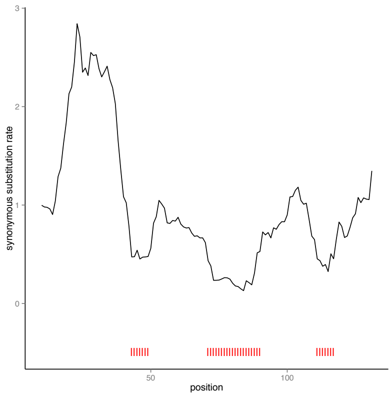

w=20

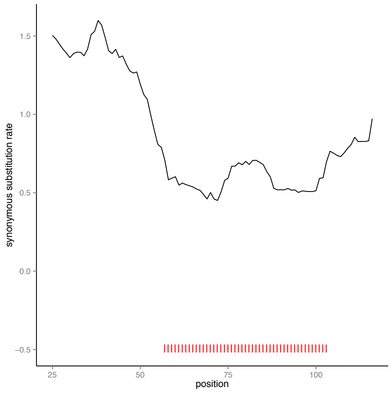

w=50

HPV6 E1

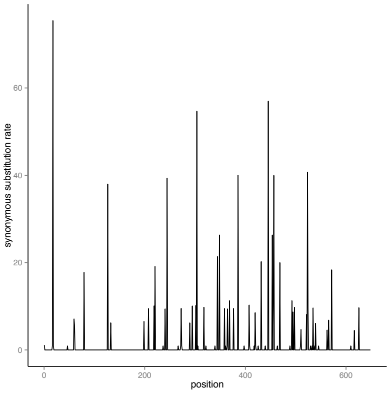

w=1

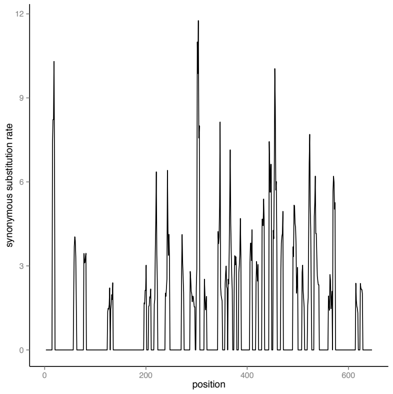

w=5

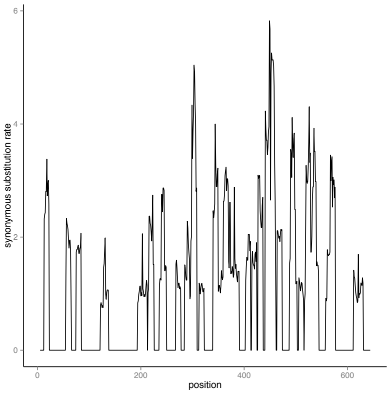

w=10

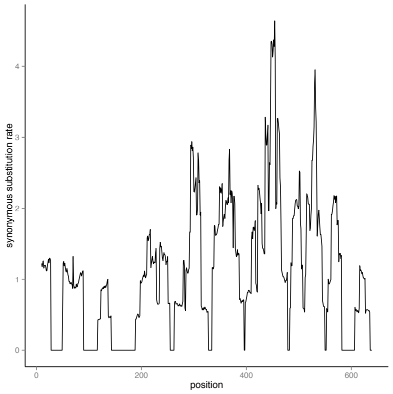

w=20

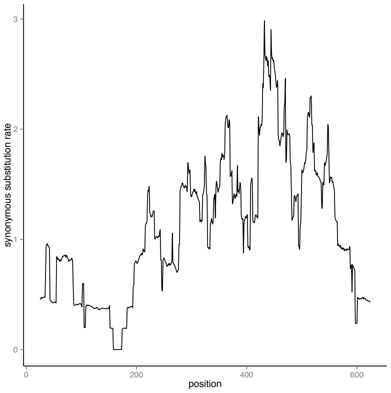

w=50

HPV6 E2

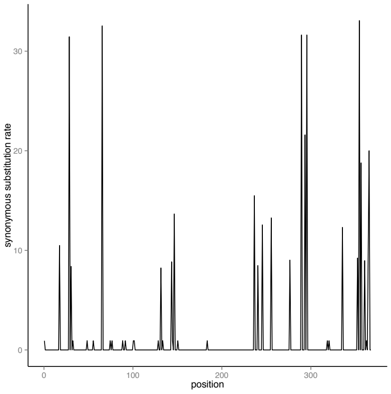

w=1

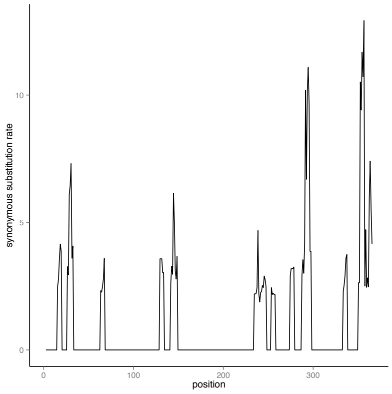

w=5

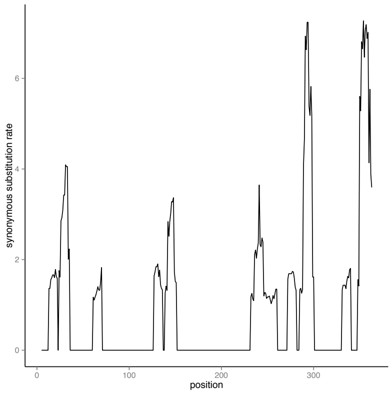

w=10

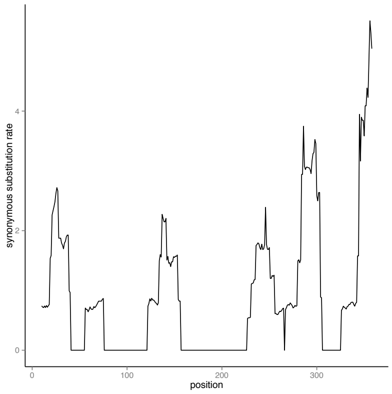

w=20

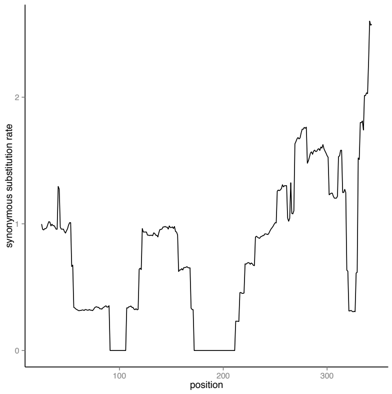

w=50

HPV6 E4

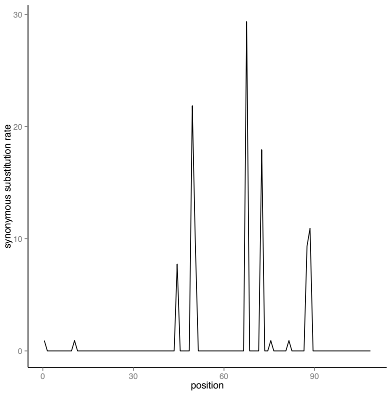

w=1

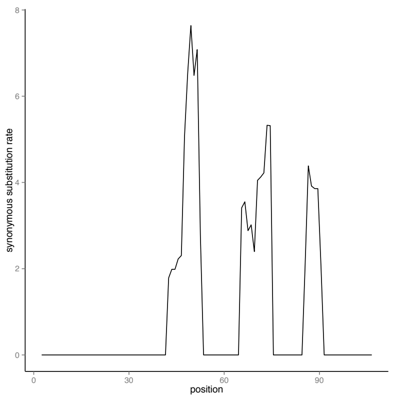

w=5

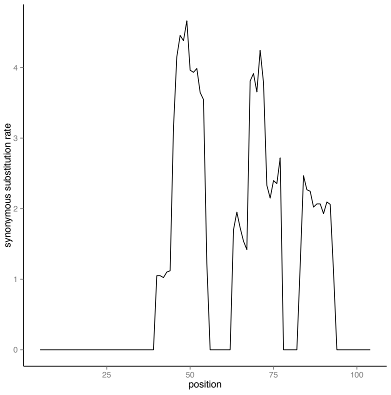

w=10

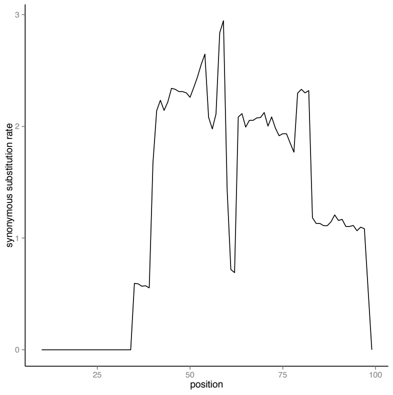

w=20

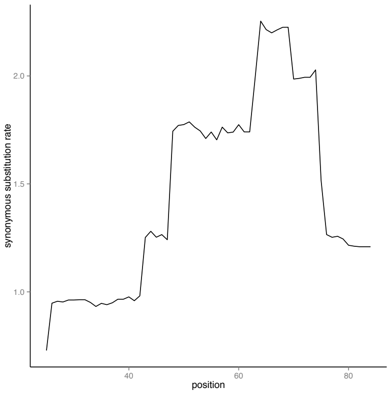

w=50

HPV6 E5A

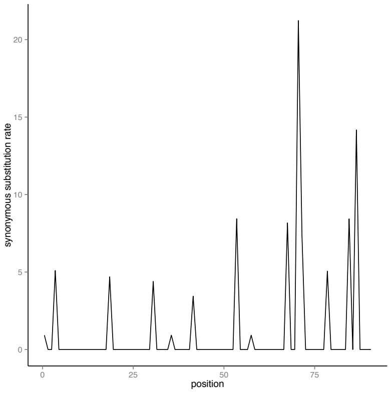

w=1

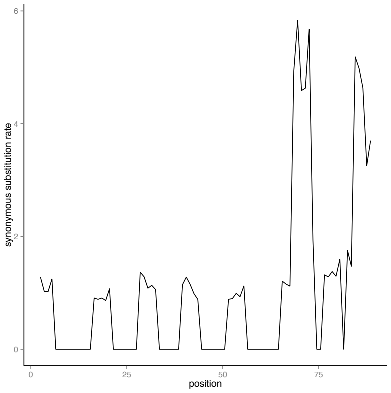

w=5

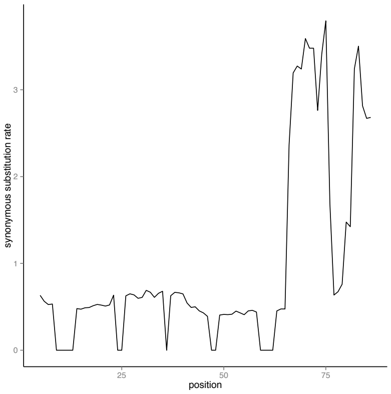

w=10

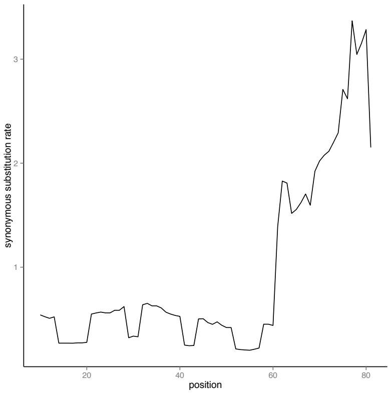

w=20

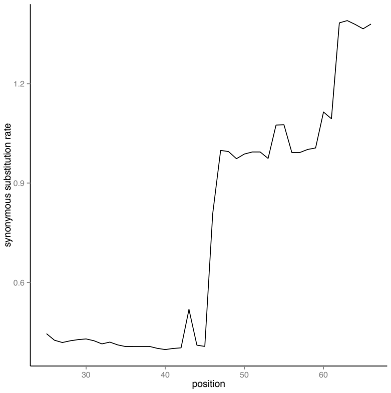

w=50

HPV6 E5B

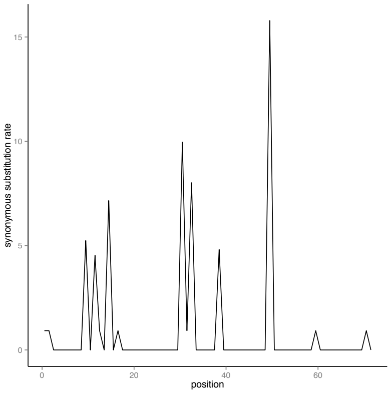

w=1

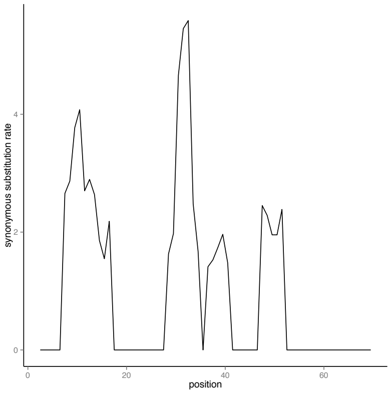

w=5

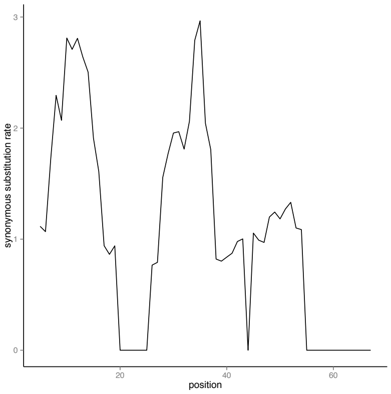

w=10

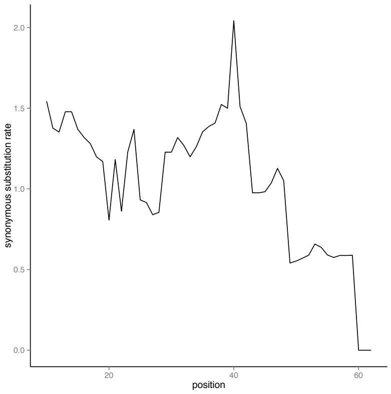

w=20

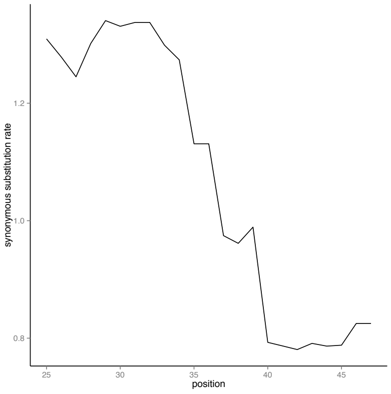

w=50

HPV6 E6

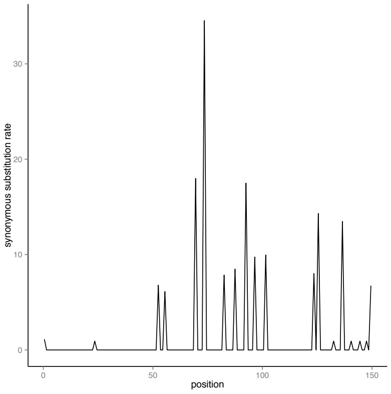

w=1

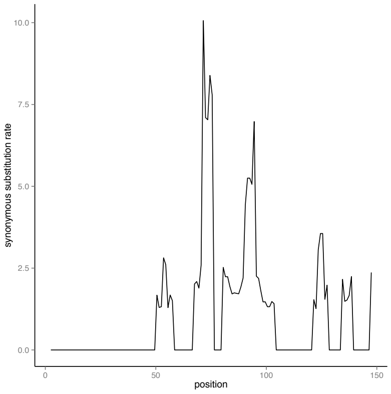

w=5

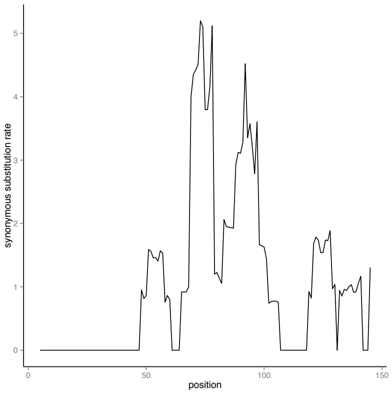

w=10

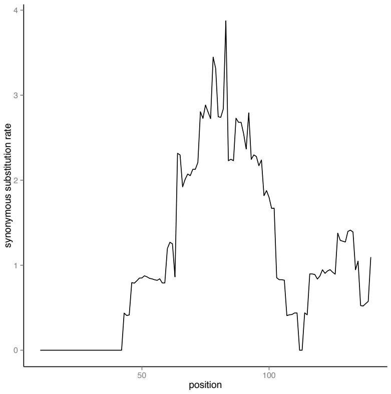

w=20

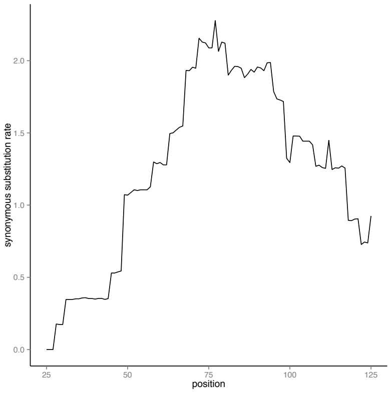

w=50

HPV6 E7

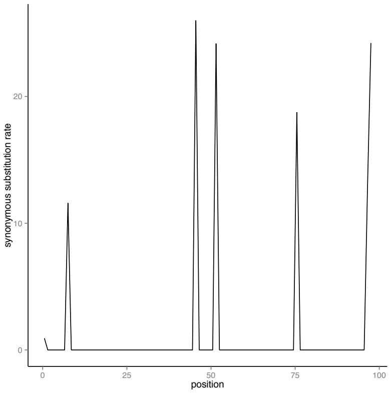

w=1

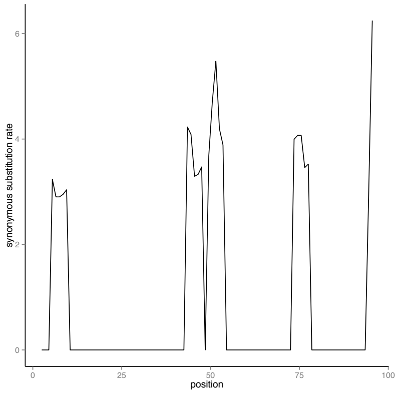

w=5

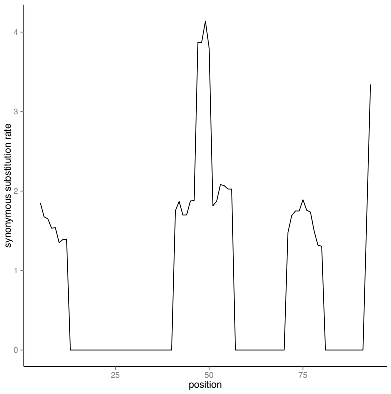

w=10

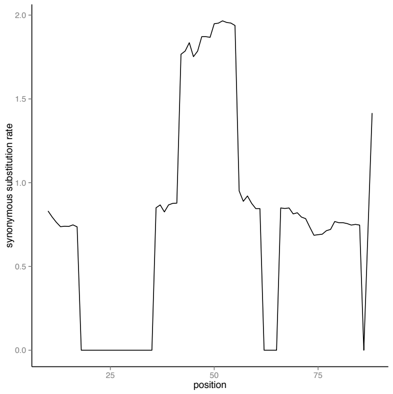

w=20

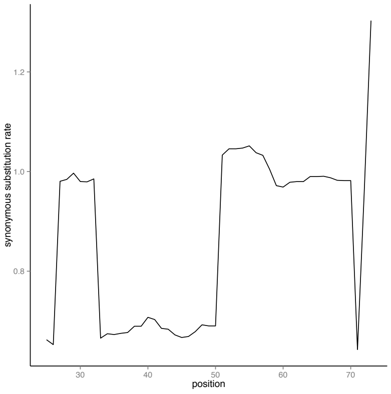

w=50

HPV6 L1

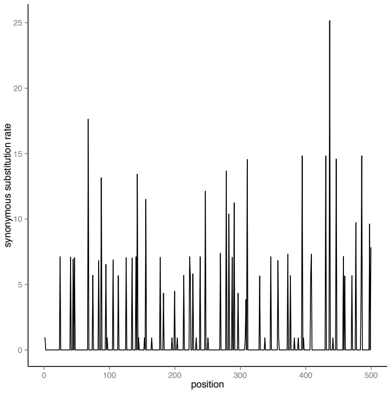

w=1

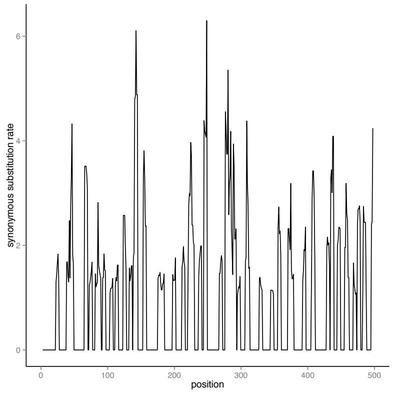

w=5

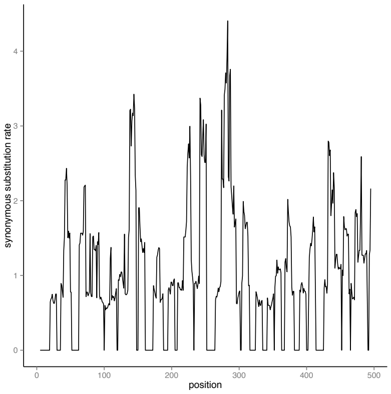

w=10

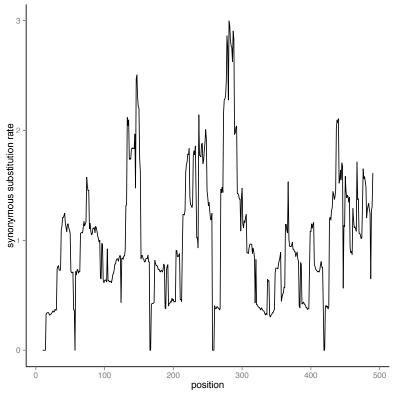

w=20

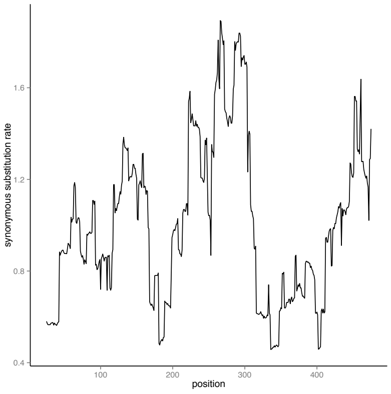

w=50

HPV6 L2

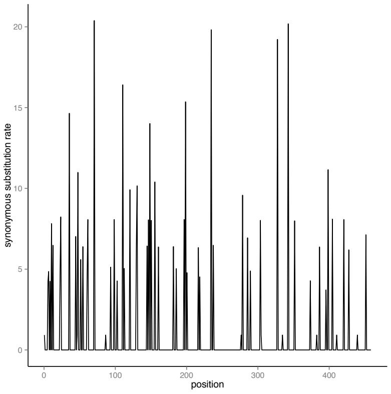

w=1

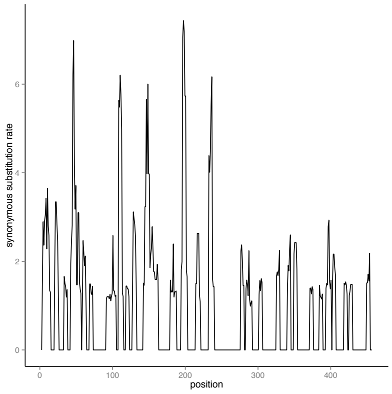

w=5

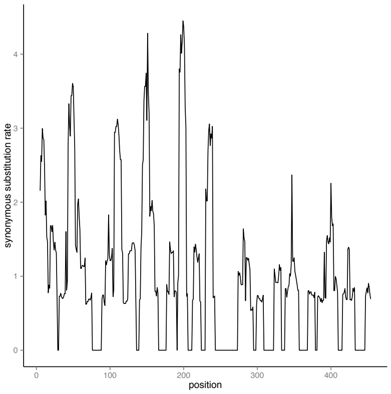

w=10

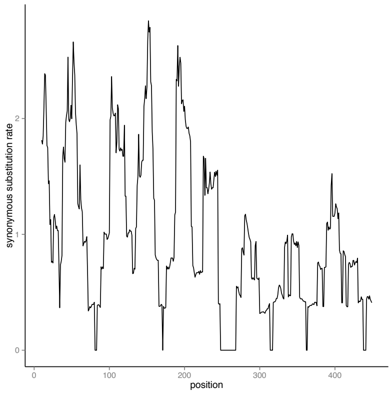

w=20

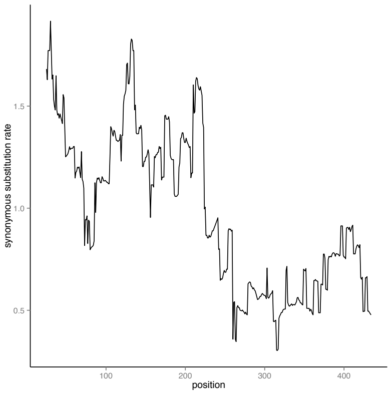

w=50

HPV16 E1

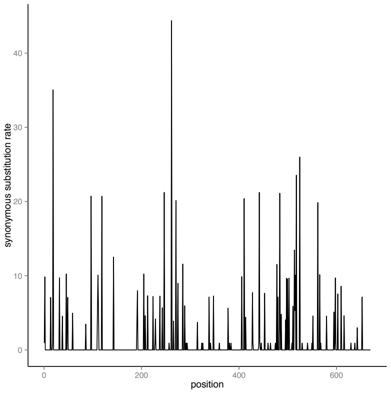

w=1

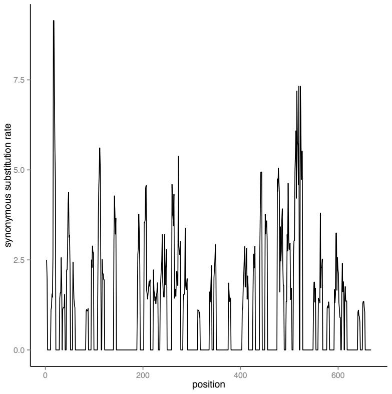

w=5

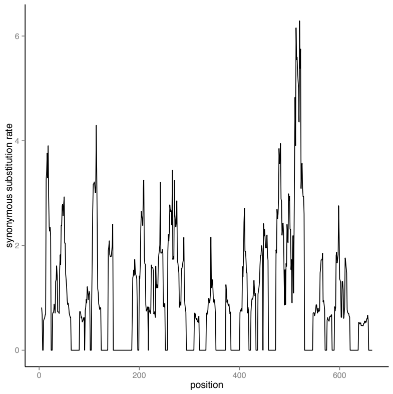

w=10

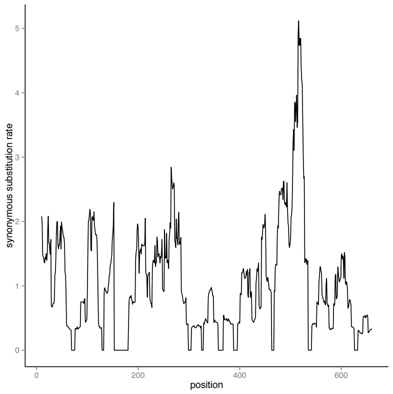

w=20

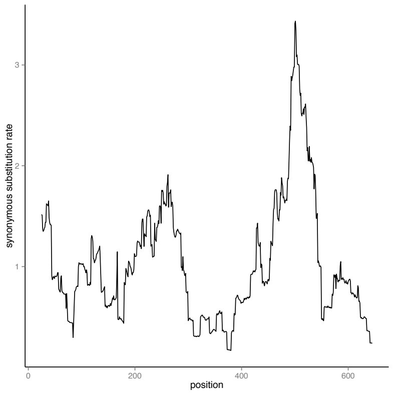

w=50

HPV16 E2

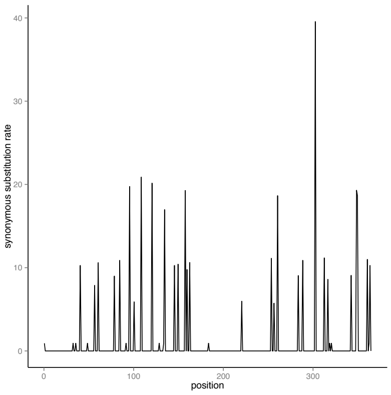

w=1

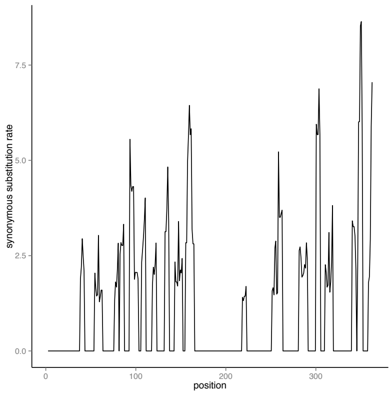

w=5

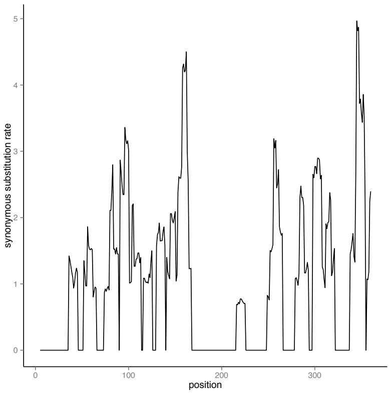

w=10

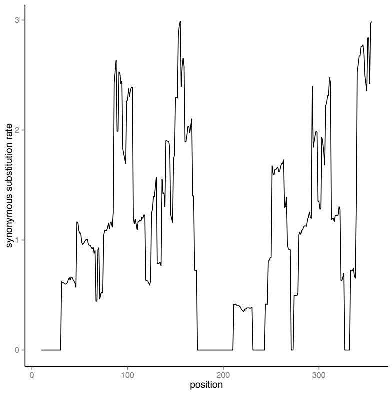

w=20

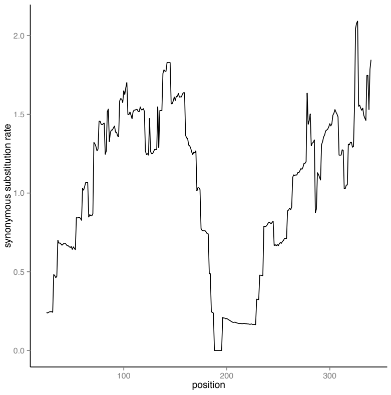

w=50

HPV16 E4

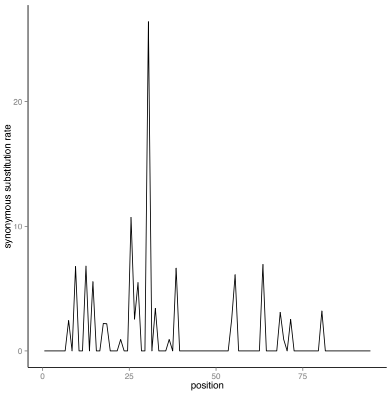

w=1

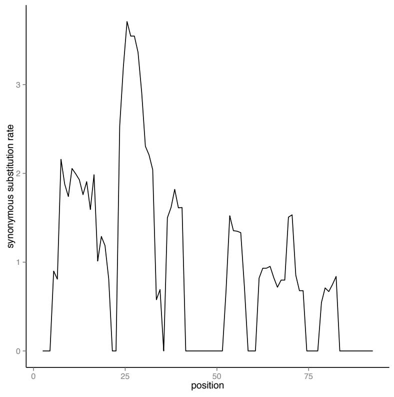

w=5

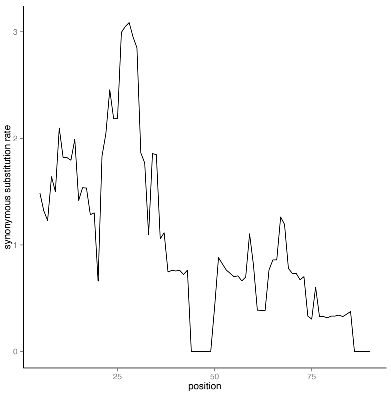

w=10

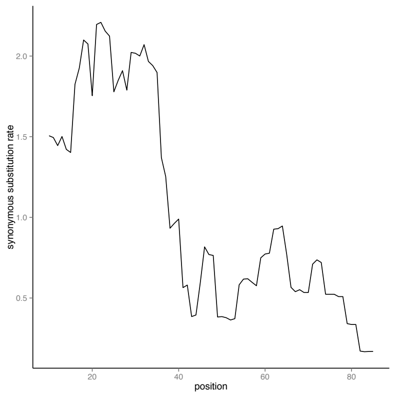

w=20

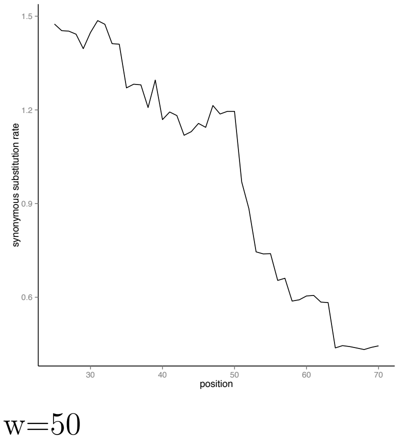

w=50

HPV16 E5A

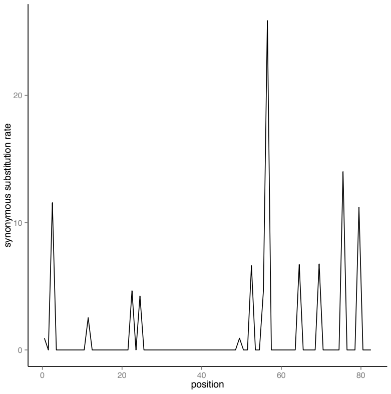

w=1

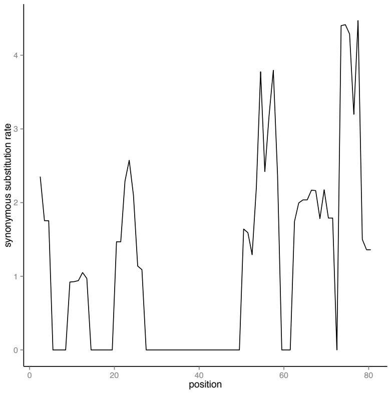

w=5

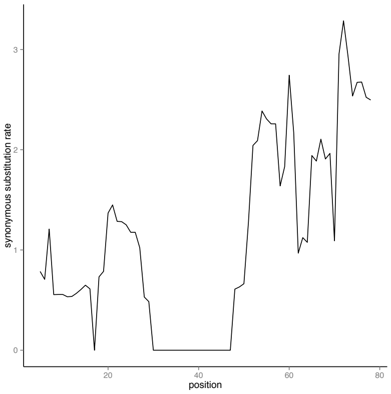

w=10

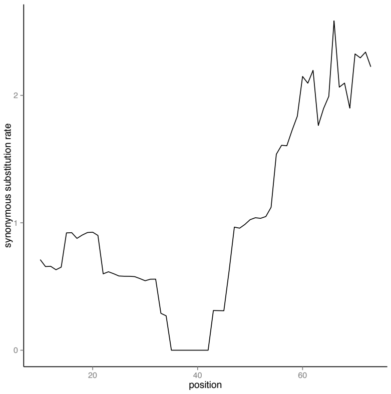

w=20

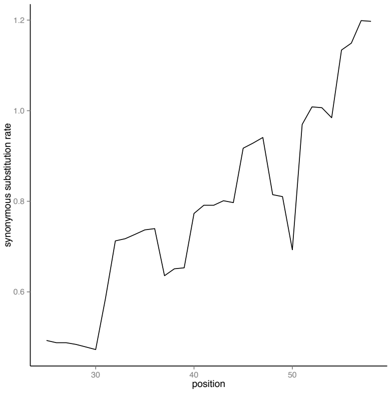

w=50

HPV16 E6

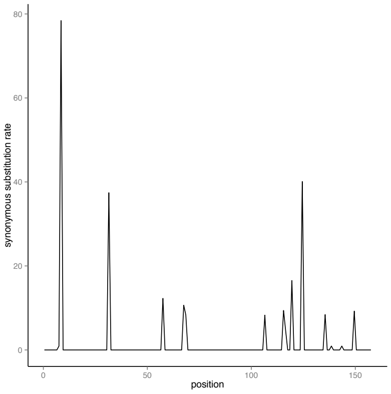

w=1

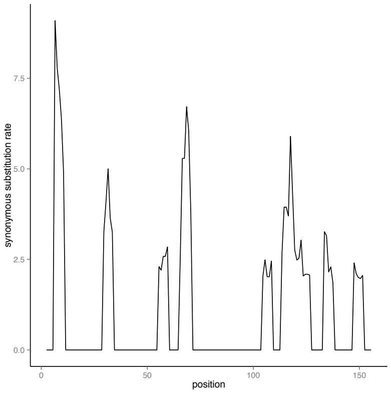

w=5

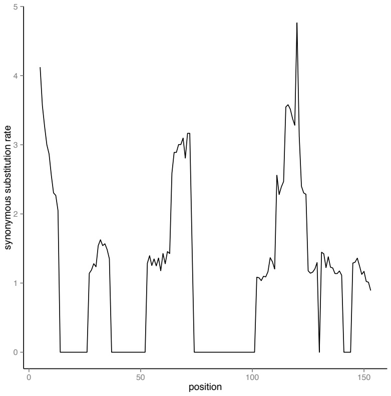

w=10

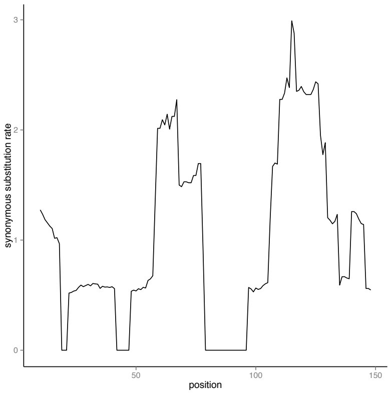

w=20

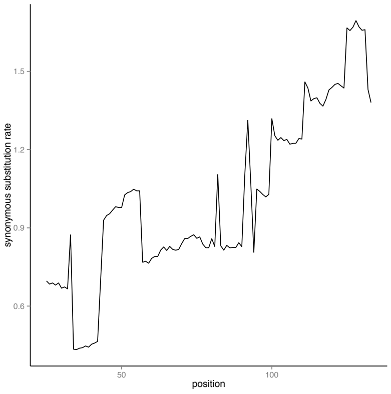

w=50

HPV16 E7

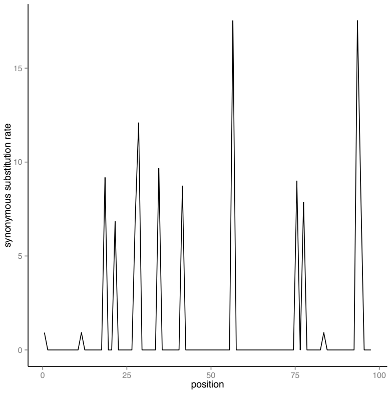

w=1

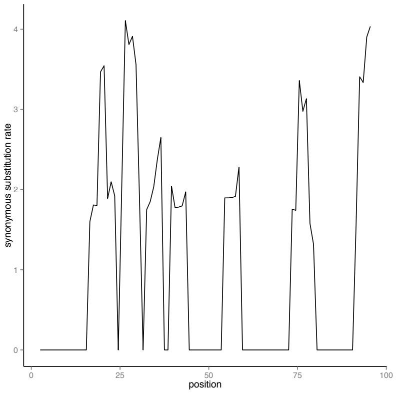

w=5

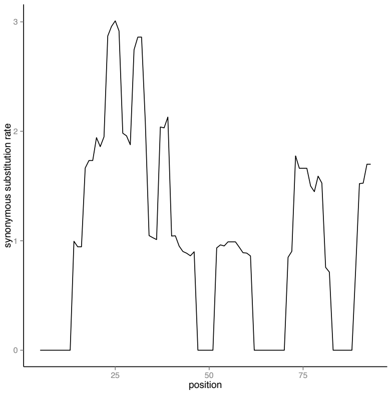

w=10

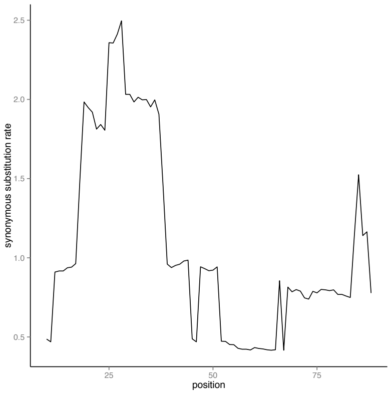

w=20

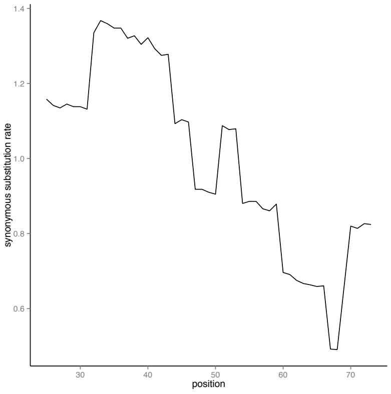

w=50

HPV16 L1

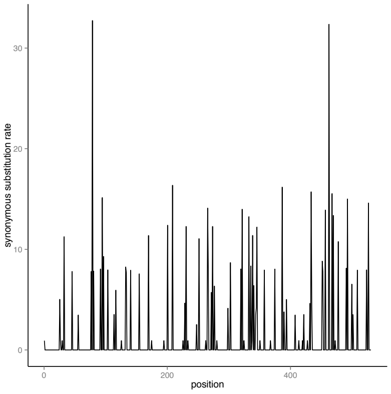

w=1

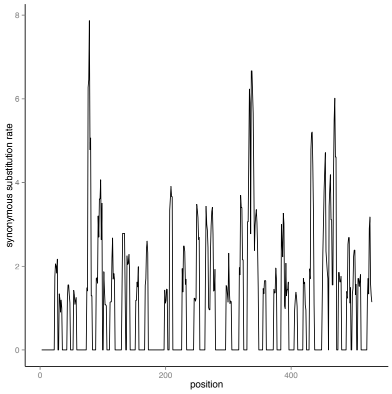

w=5

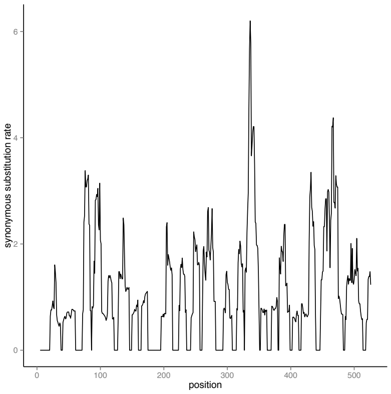

w=10

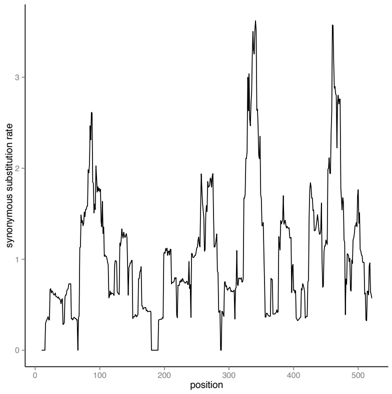

w=20

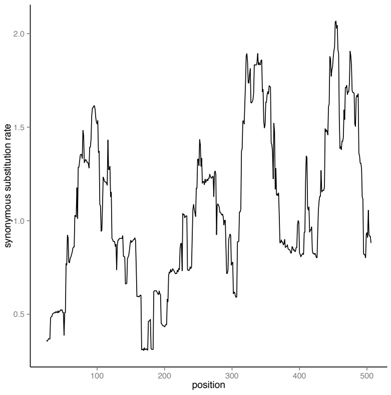

w=50

HPV16 L2

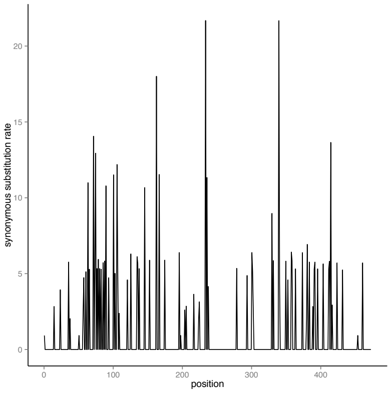

w=1

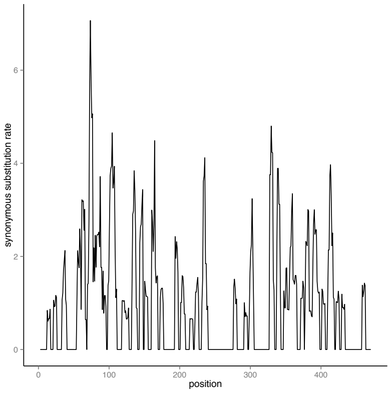

w=5

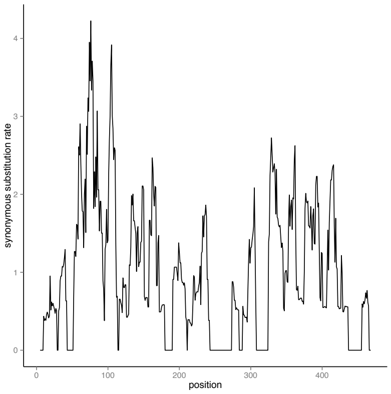

w=10

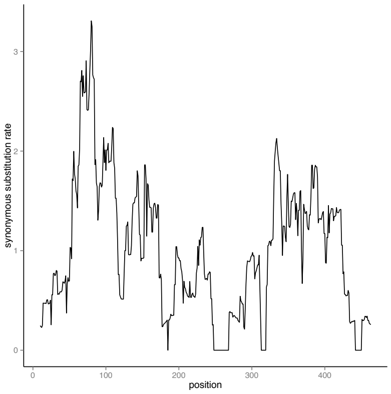

w=20

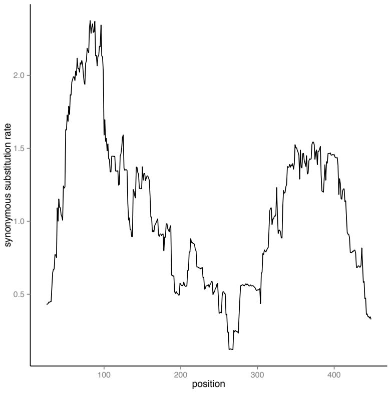

w=50

HRSV F

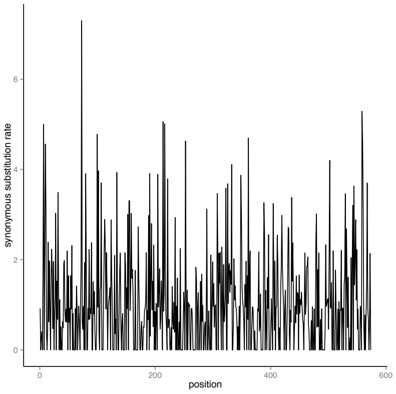

w=1

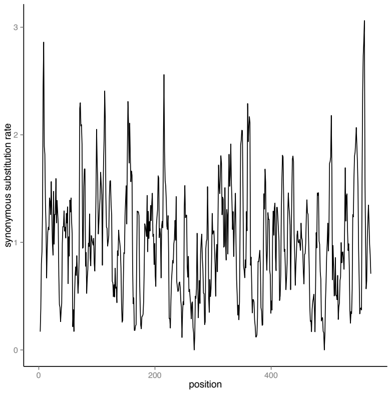

w=5

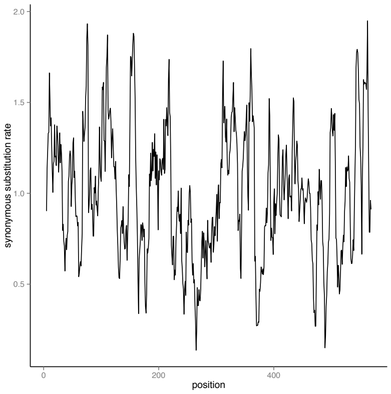

w=10

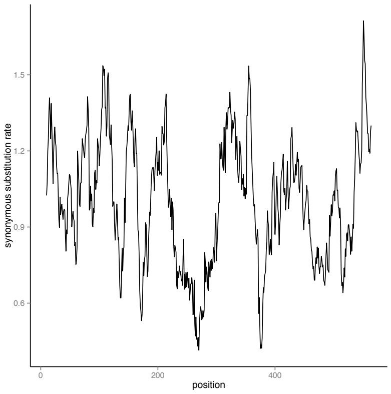

w=20

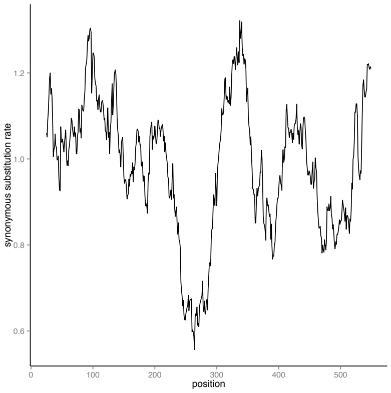

w=50

HRSV G

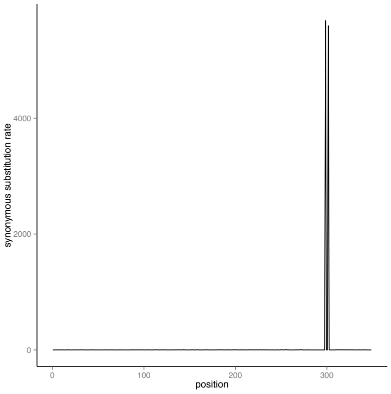

w=1

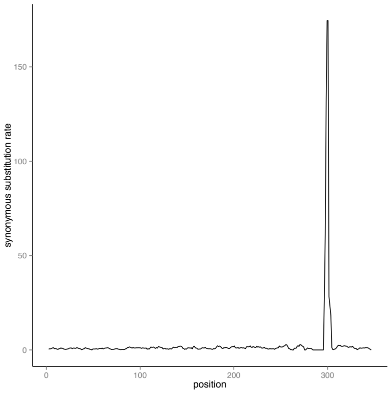

w=5

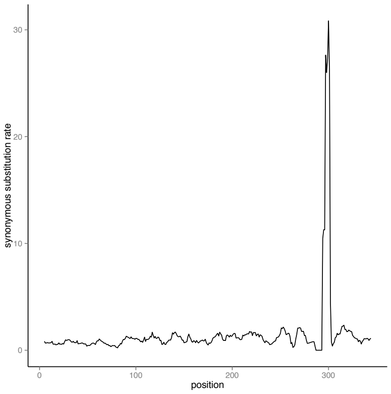

w=10

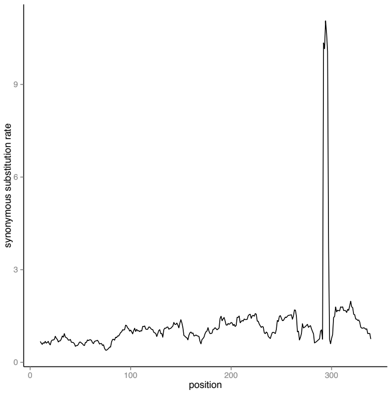

w=20

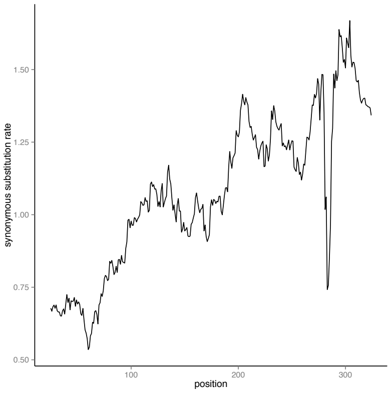

w=50

HRSV L

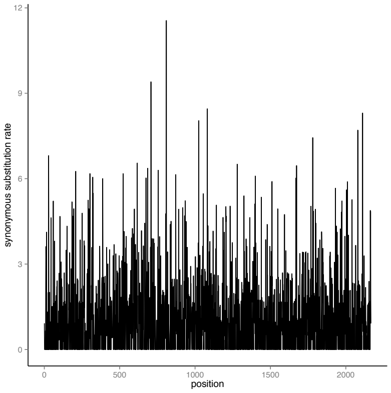

w=1

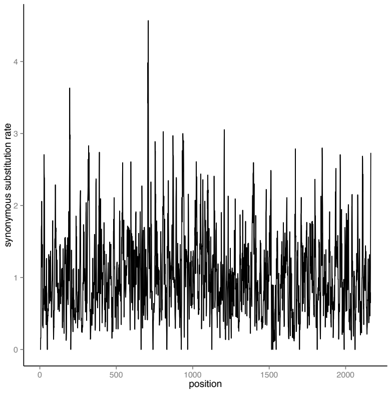

w=5

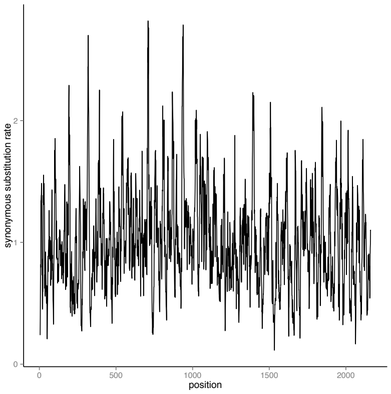

w=10

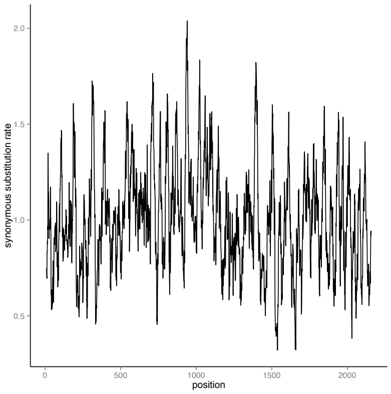

w=20

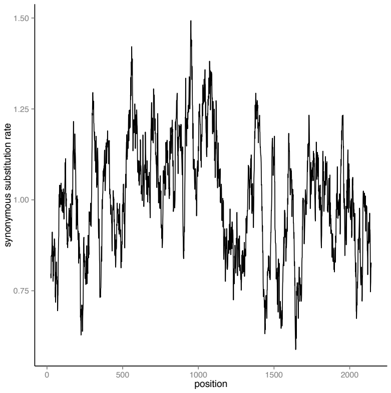

w=50

HRSV M

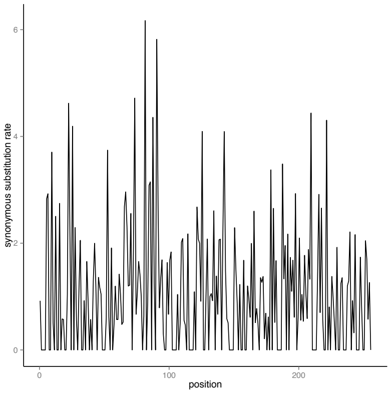

w=1

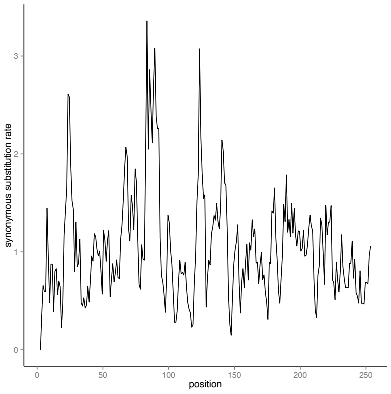

w=5

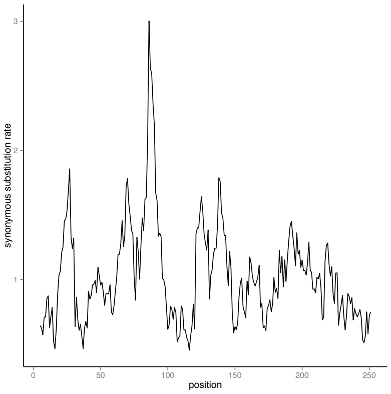

w=10

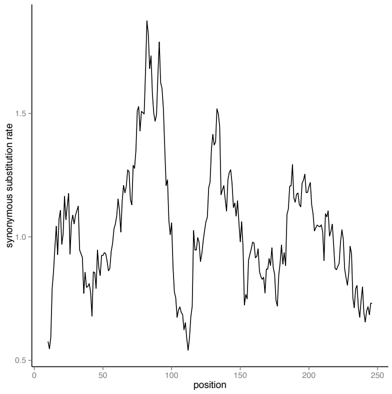

w=20

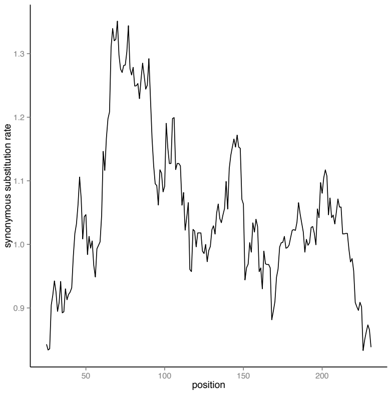

w=50

HRSV N

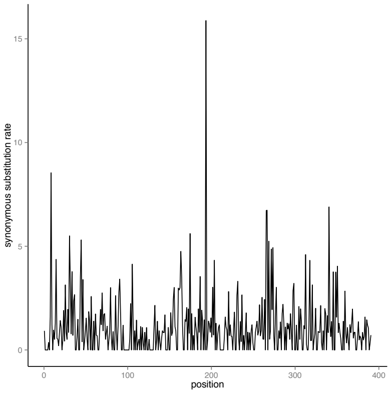

w=1

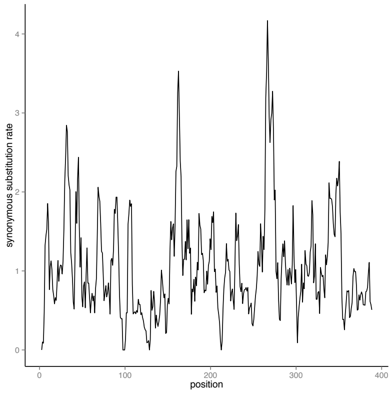

w=5

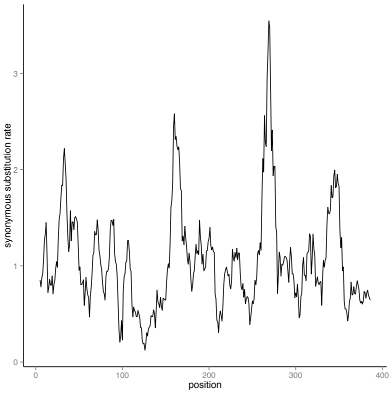

w=10

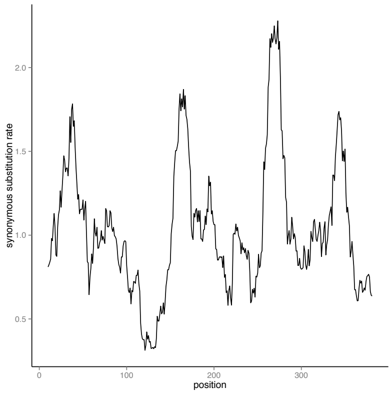

w=20

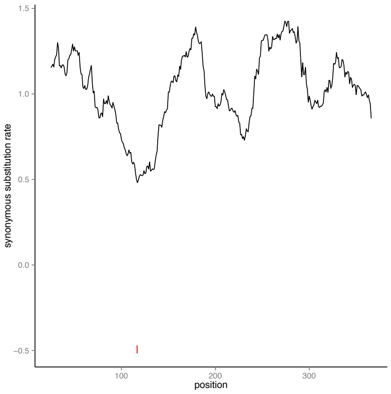

w=50

HRSV NS1

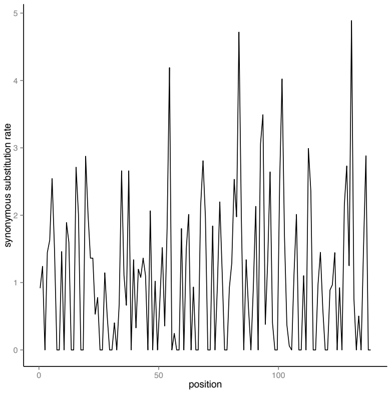

w=1

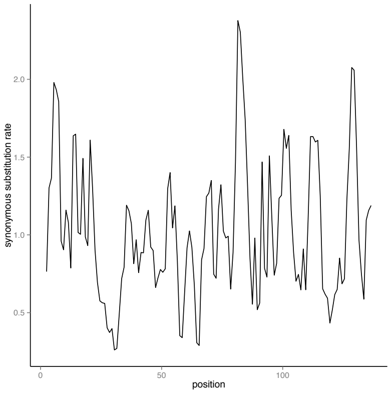

w=5

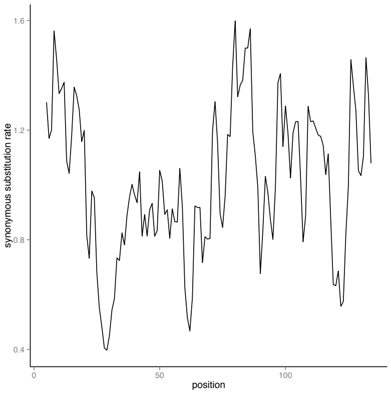

w=10

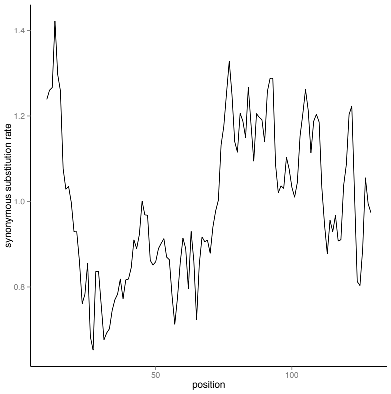

w=20

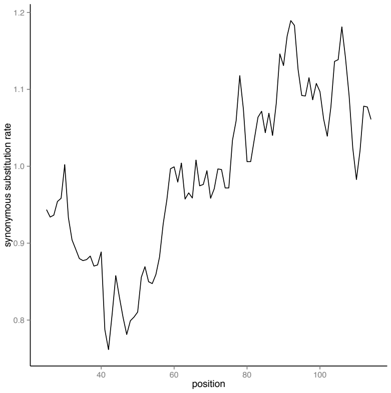

w=50

HRSV NS2

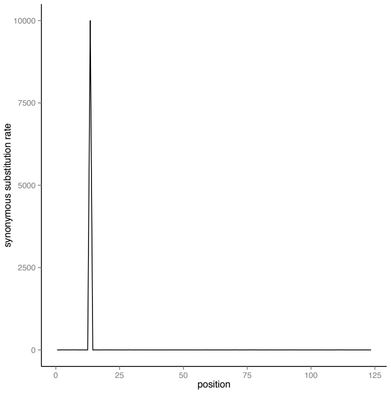

w=1

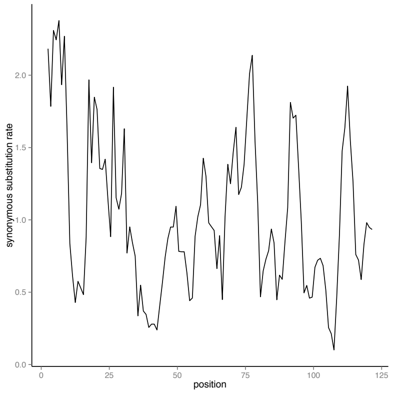

w=5

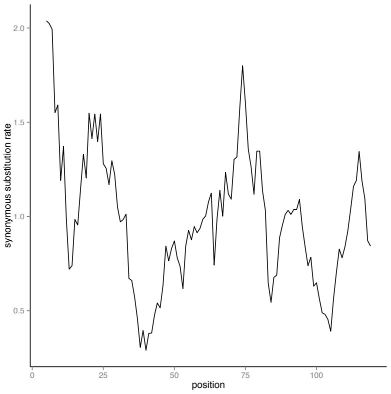

w=10

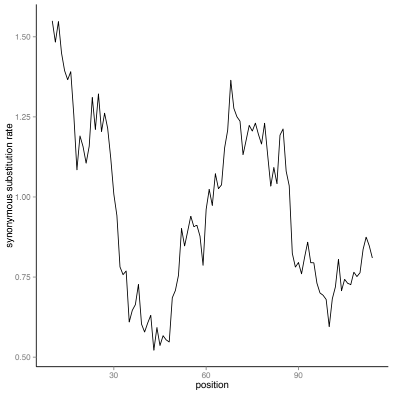

w=20

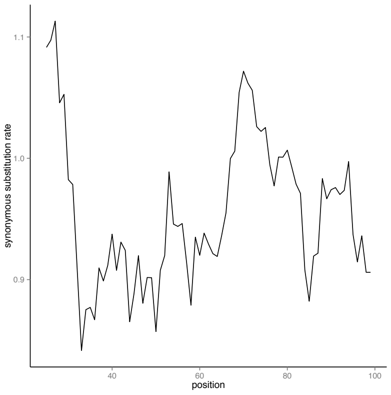

w=50

HRSV P

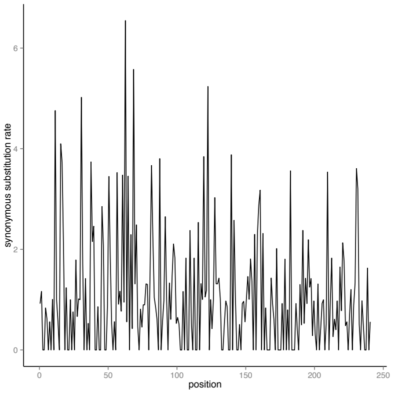

w=1

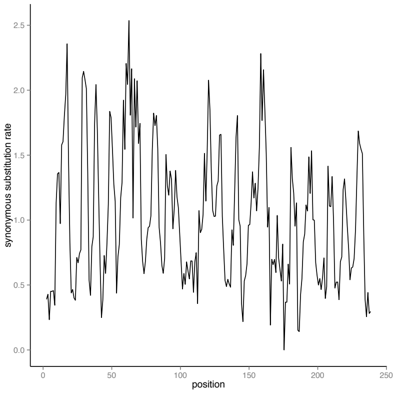

w=5

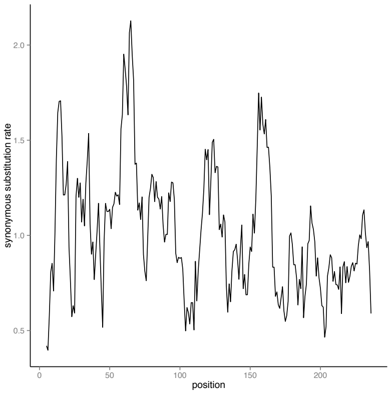

w=10

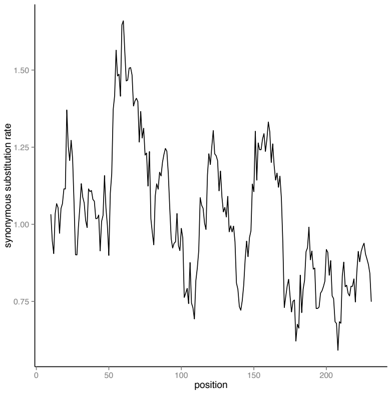

w=20

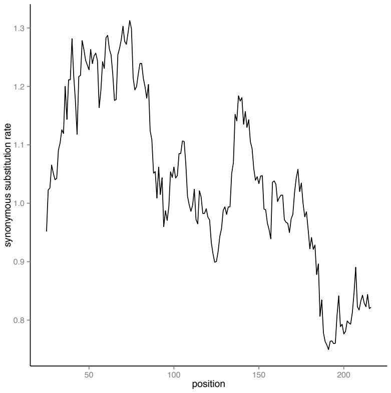

w=50

HRSV SH

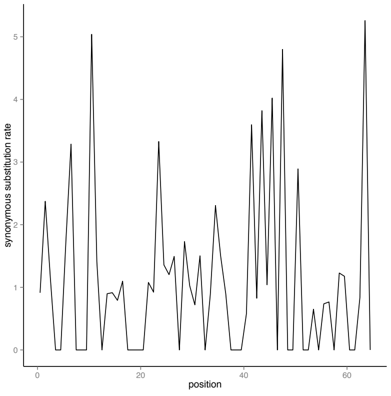

w=1

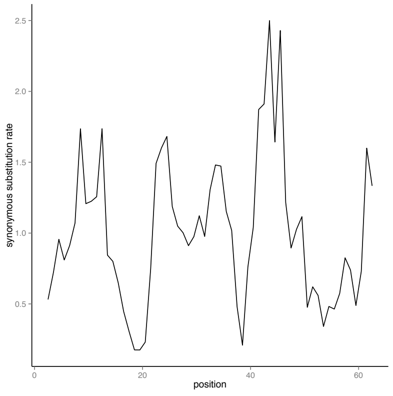

w=5

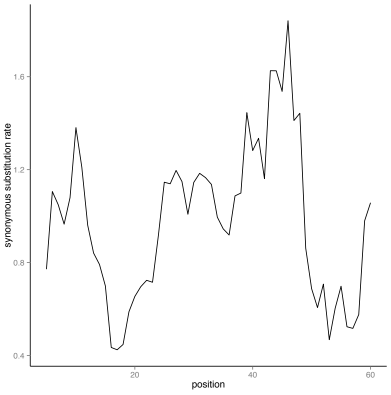

w=10

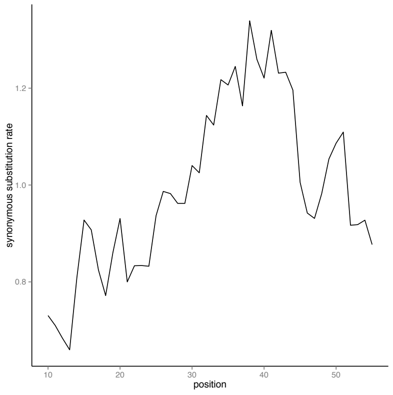

w=20

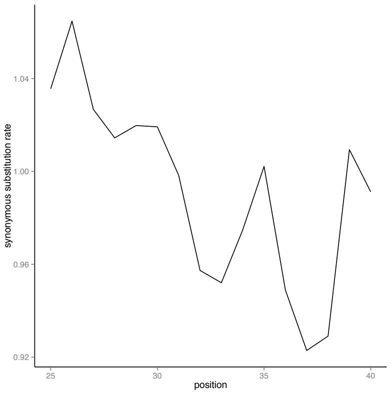

w=50

IBDV polymerase

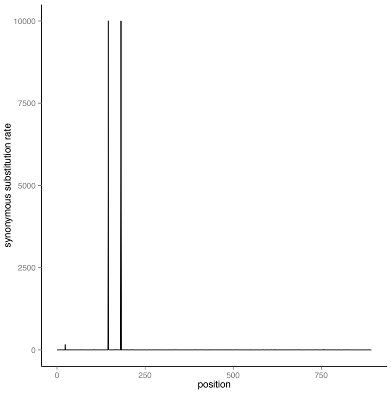

w=1

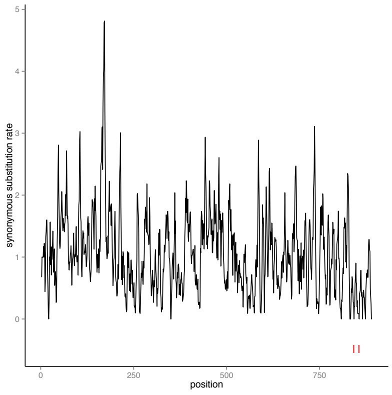

w=5

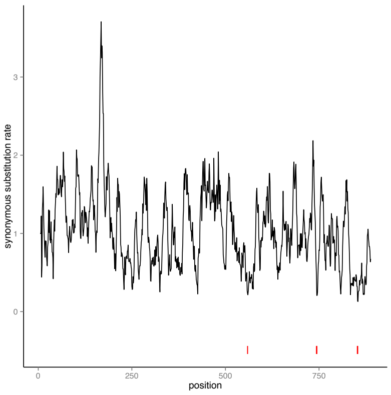

w=10

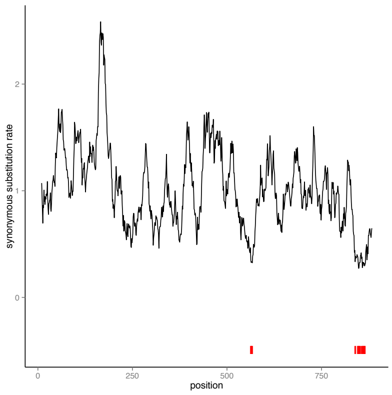

w=20

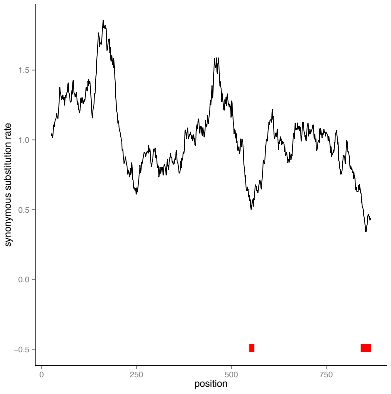

w=50

IBDV polyprotein

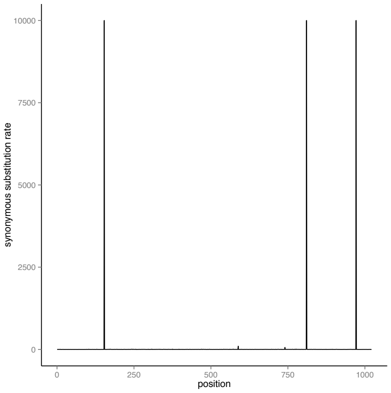

w=1

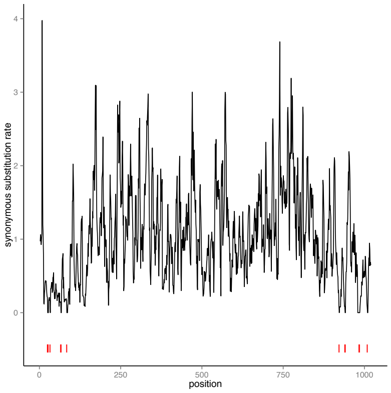

w=5

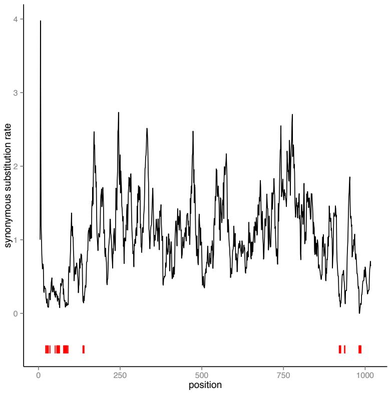

w=10

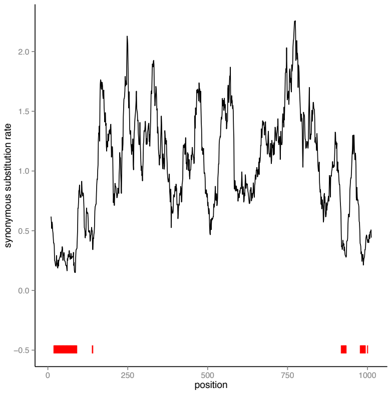

w=20

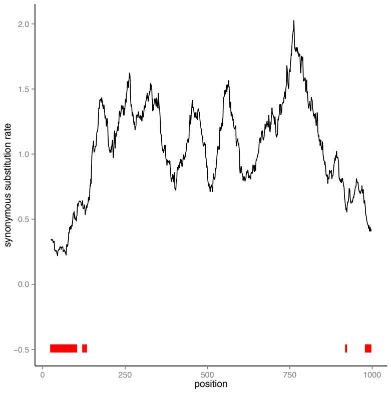

w=50

JCV large T

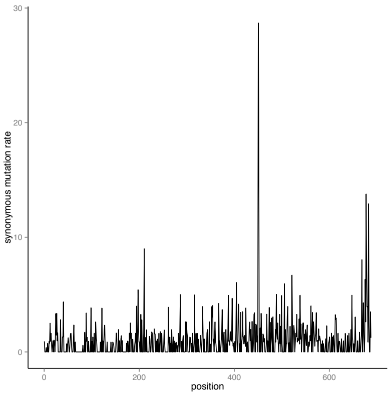

w=1

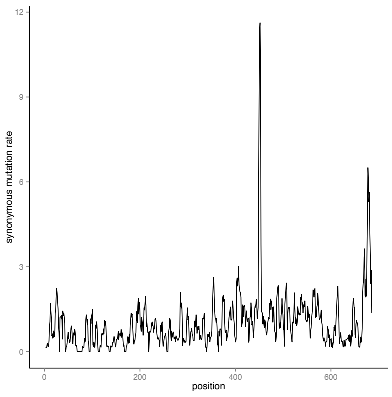

w=5

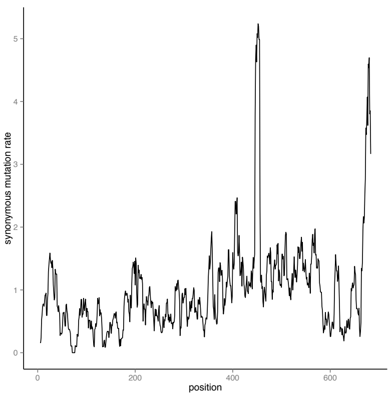

w=10

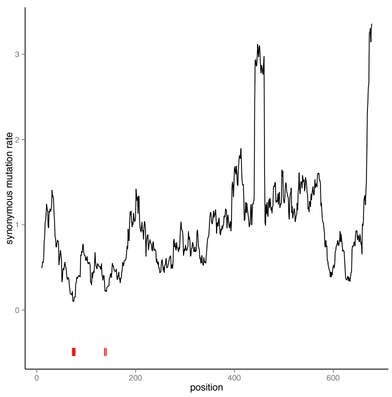

w=20

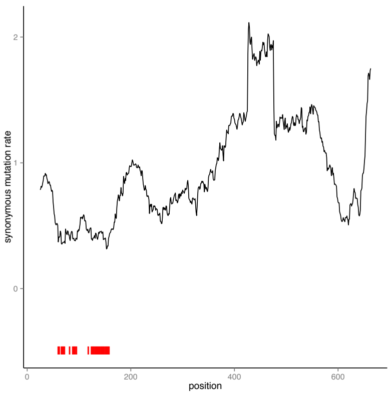

w=50

JCV small T

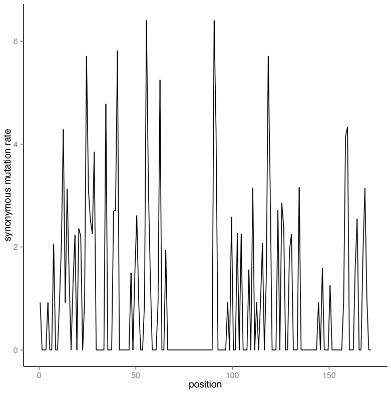

w=1

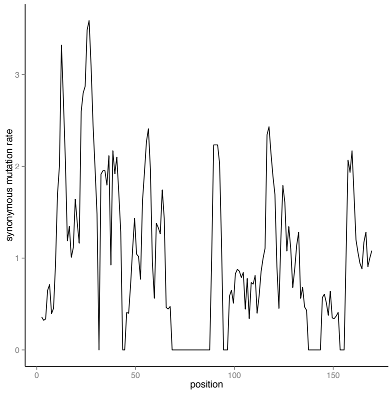

w=5

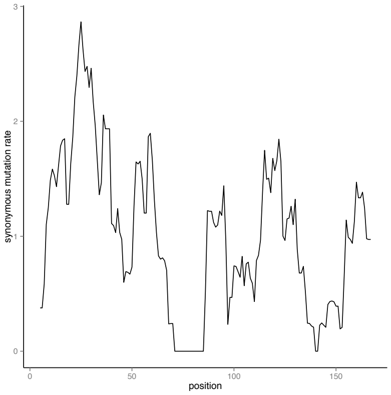

w=10

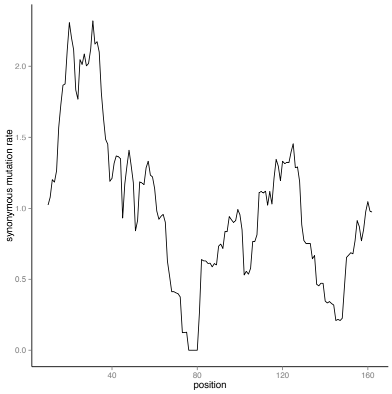

w=20

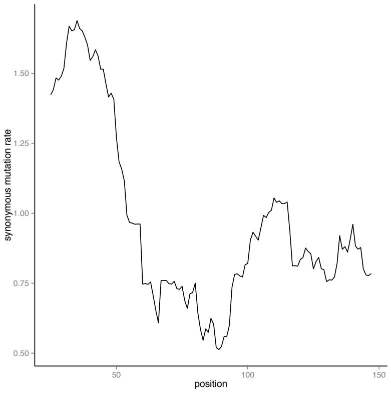

w=50

JCV VP1

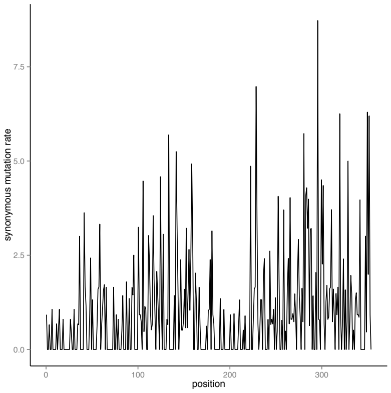

w=1

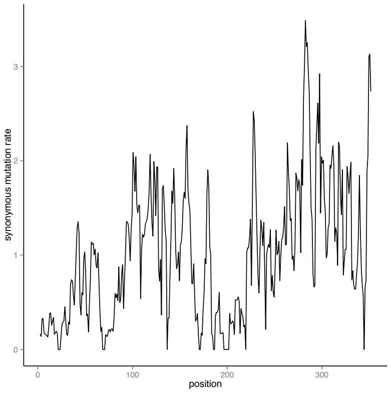

w=5

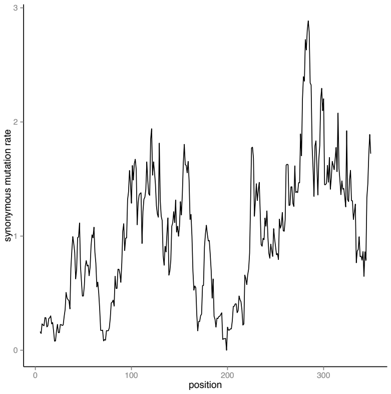

w=10

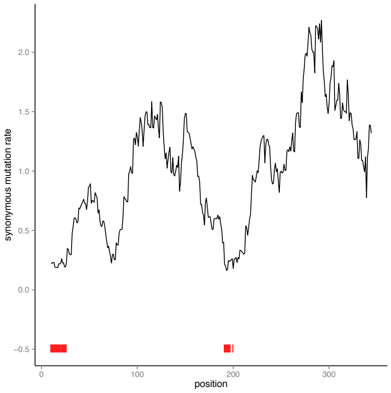

w=20

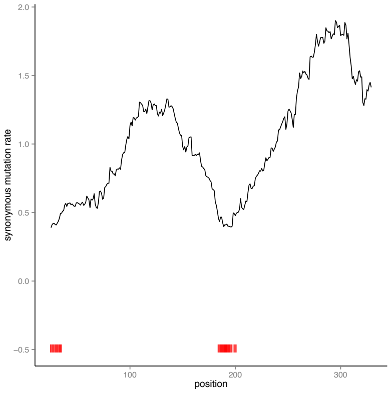

w=50

JCV VP2

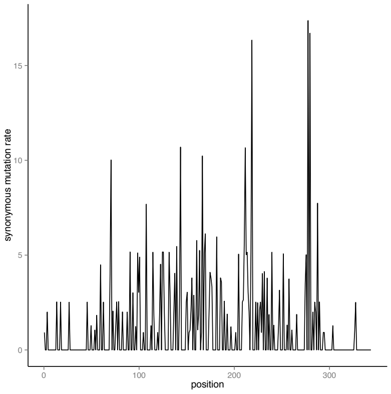

w=1

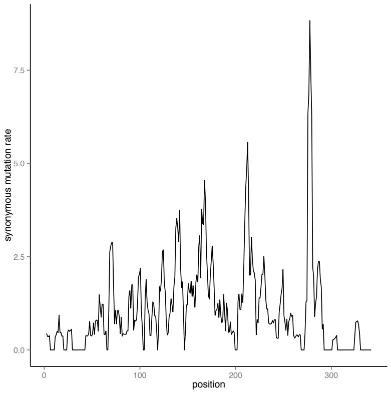

w=5

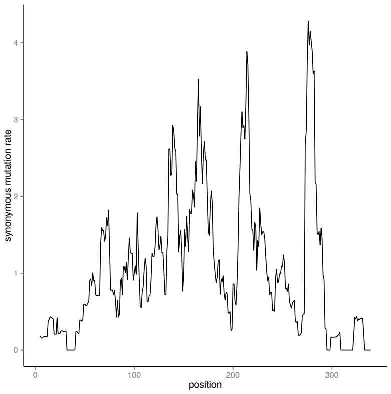

w=10

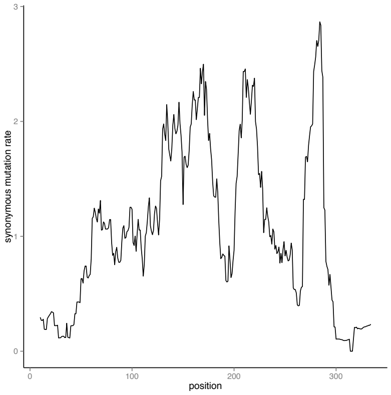

w=20

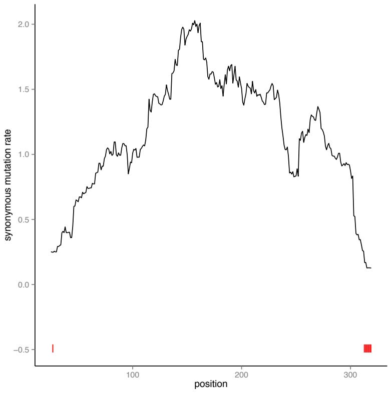

w=50

JCV VP3

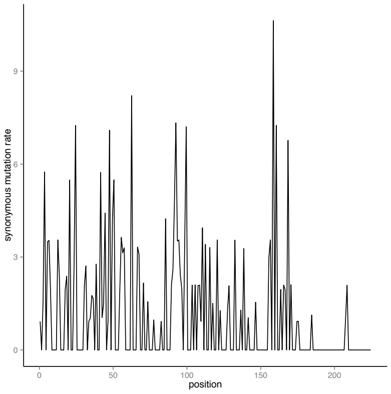

w=1

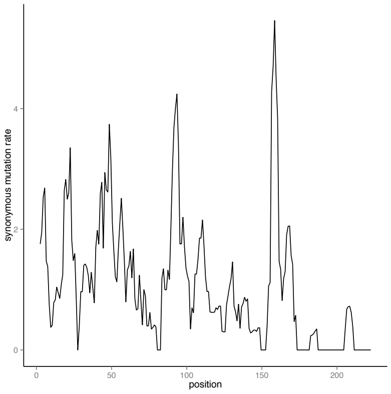

w=5

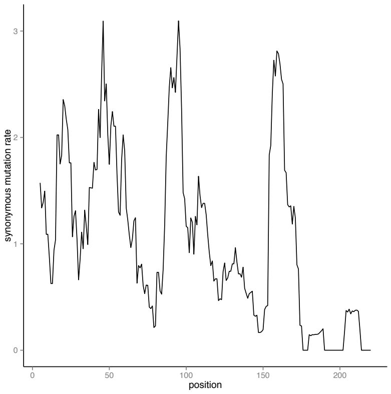

w=10

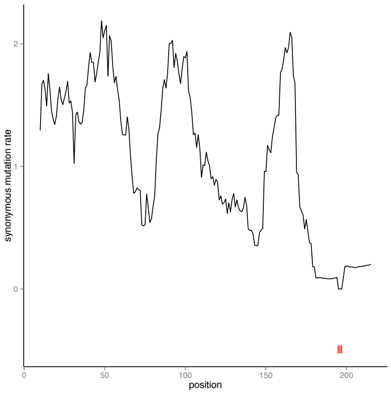

w=20

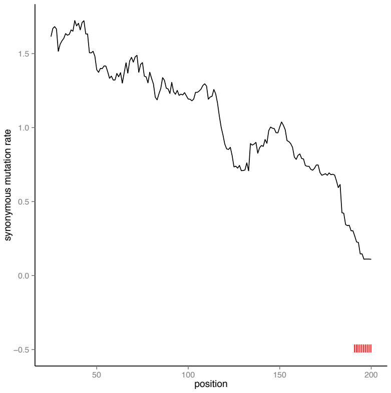

w=50

JCV agno

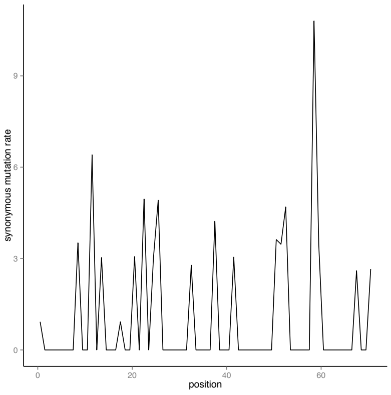

w=1

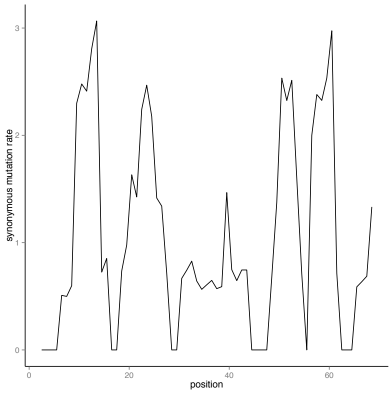

w=5

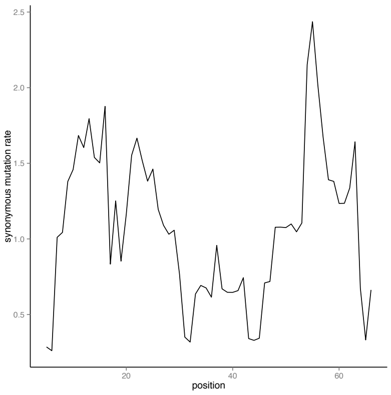

w=10

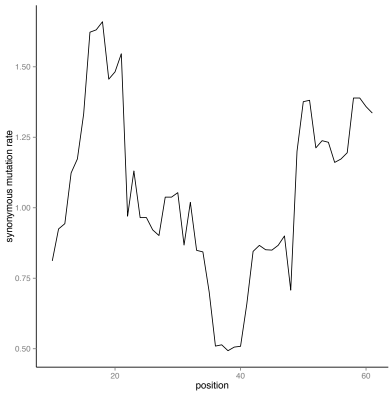

w=20

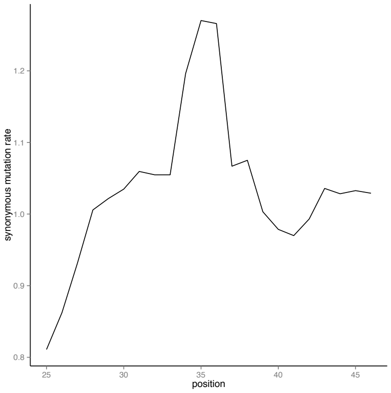

w=50

JEV polyprotein

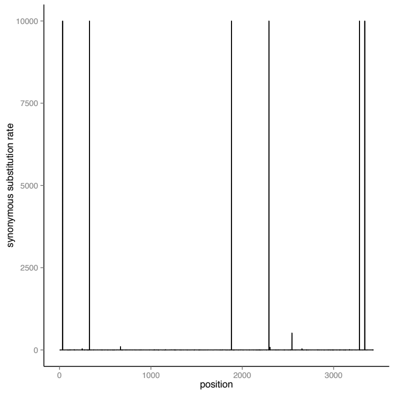

w=1

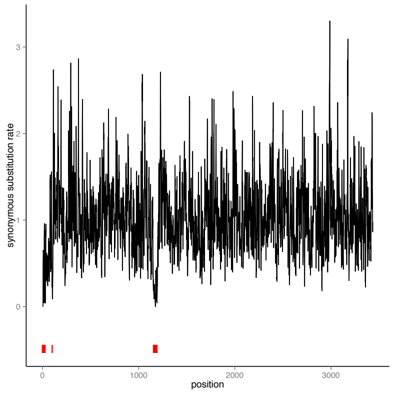

w=5

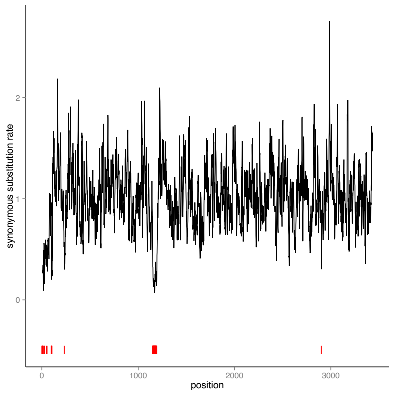

w=10

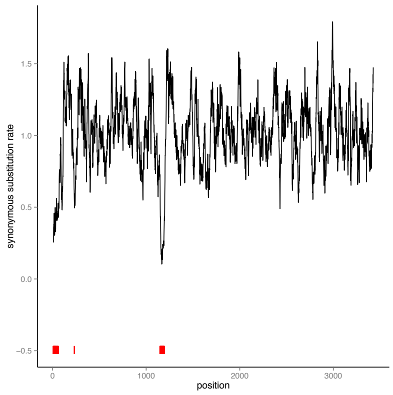

w=20

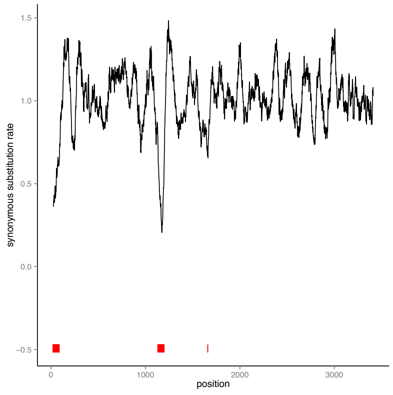

w=50

MSV RepA

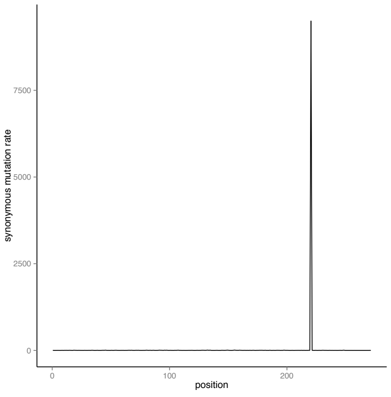

w=1

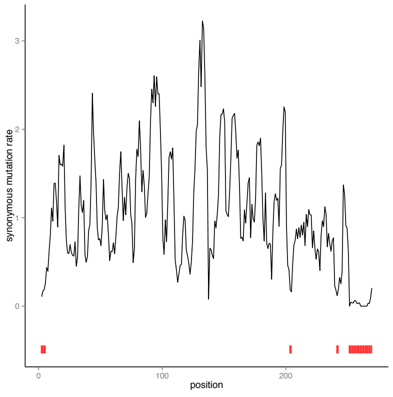

w=5

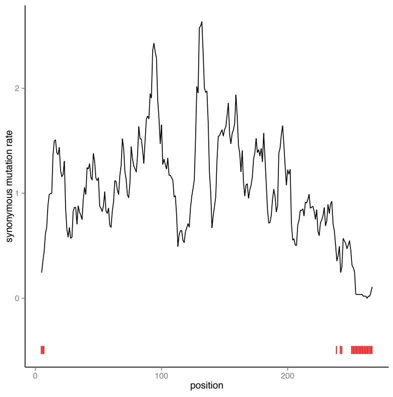

w=10

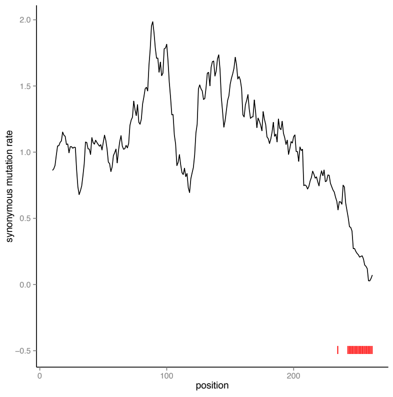

w=20

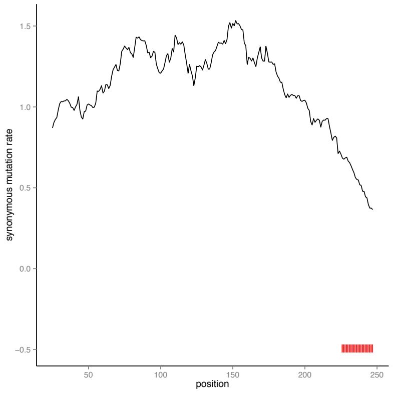

w=50

MSV RepB

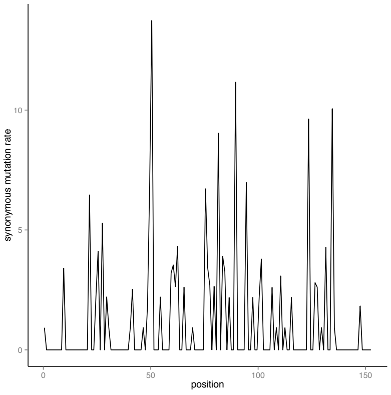

w=1

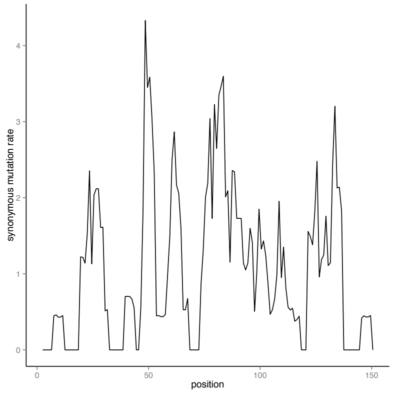

w=5

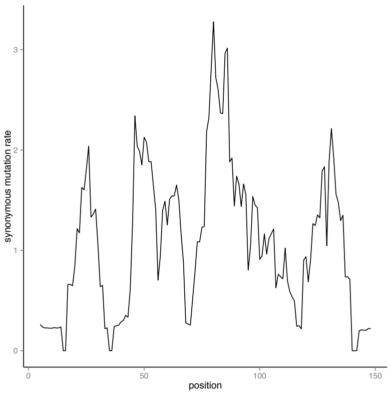

w=10

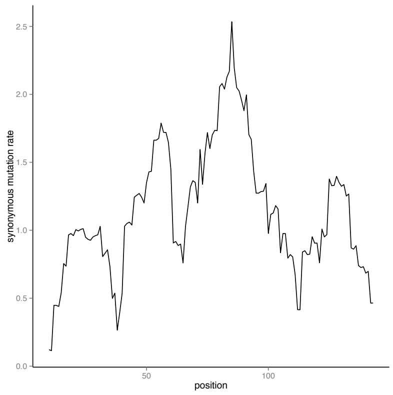

w=20

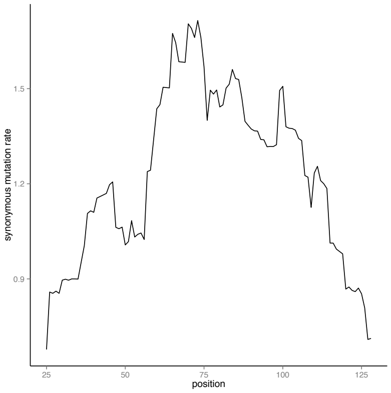

w=50

MSV MP

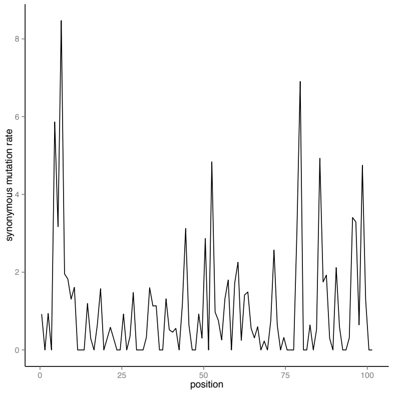

w=1

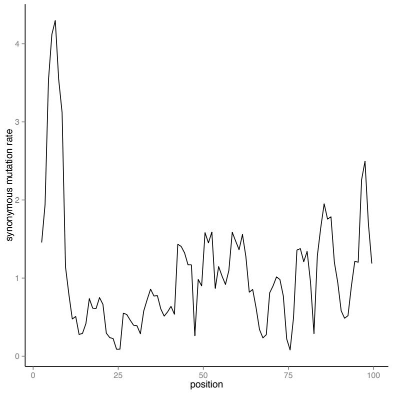

w=5

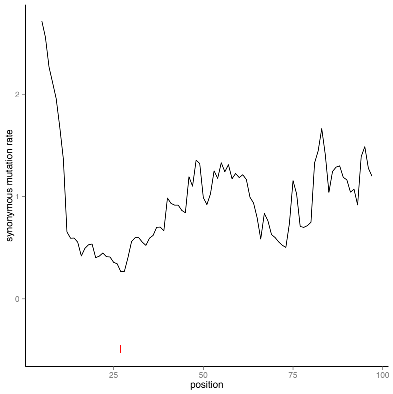

w=10

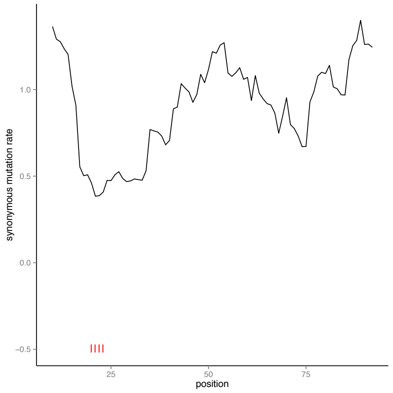

w=20

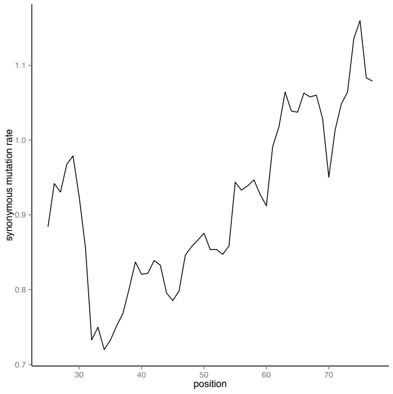

w=50

MSV CP

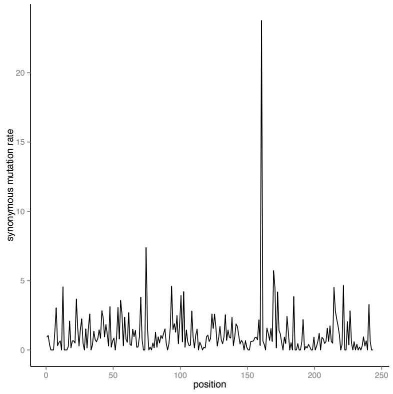

w=1

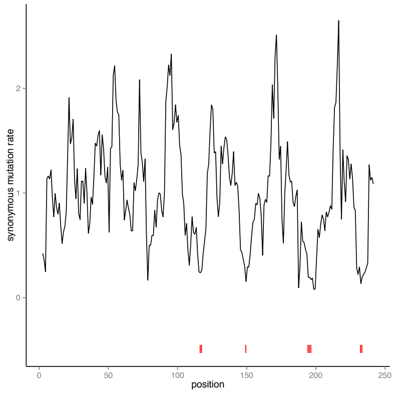

w=5

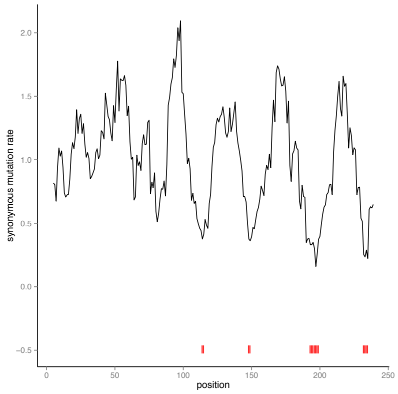

w=10

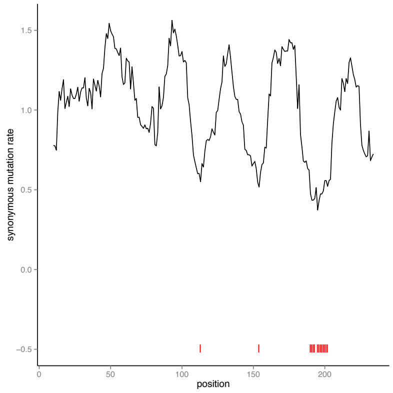

w=20

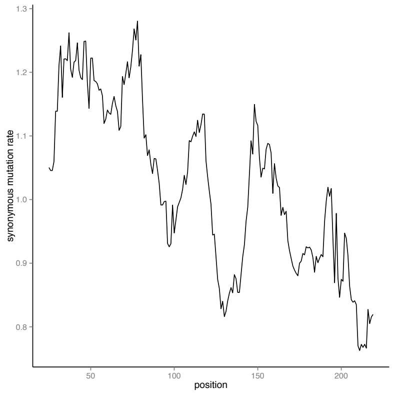

w=50

Newcastle F

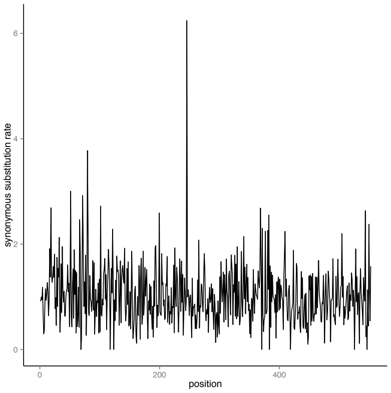

w=1

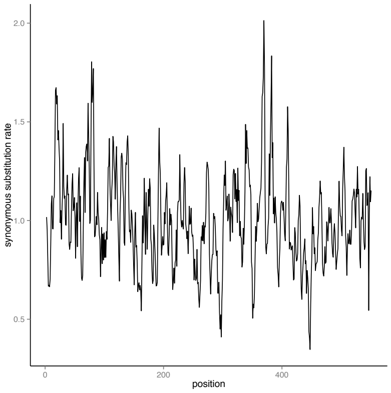

w=5

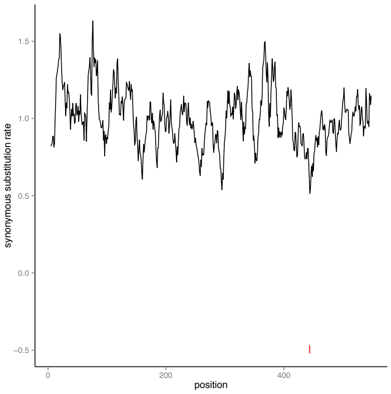

w=10

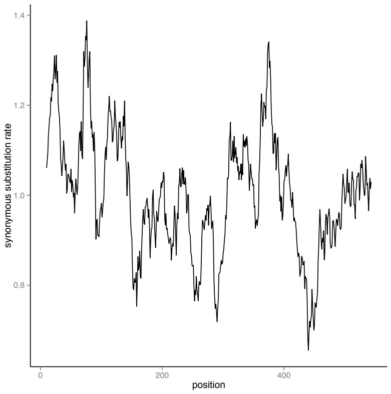

w=20

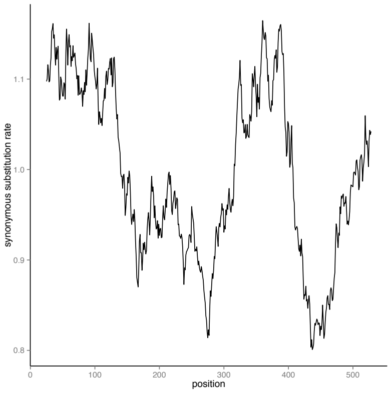

w=50

Newcastle HN

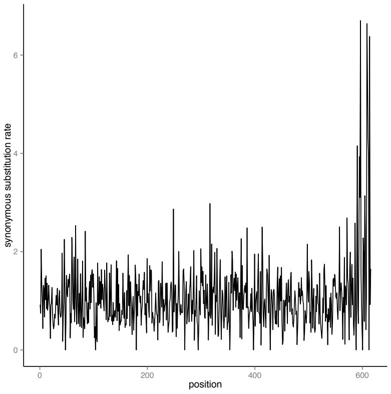

w=1

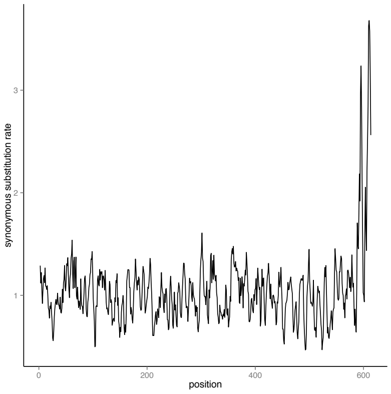

w=5

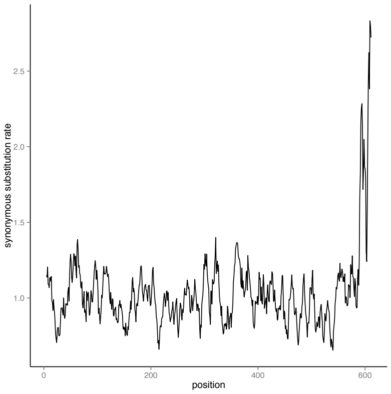

w=10

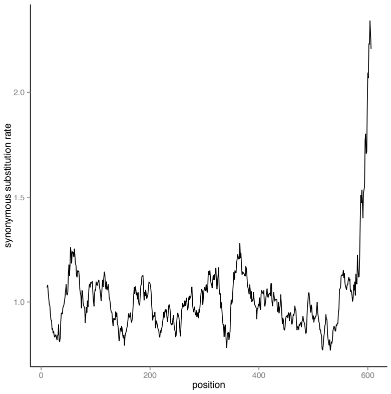

w=20

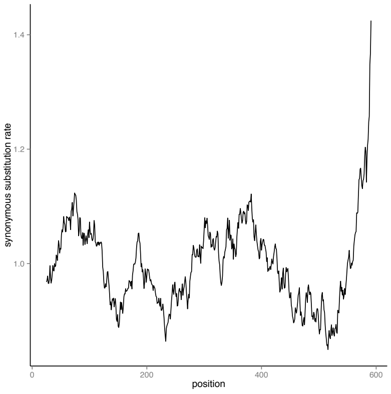

w=50

Newcastle L

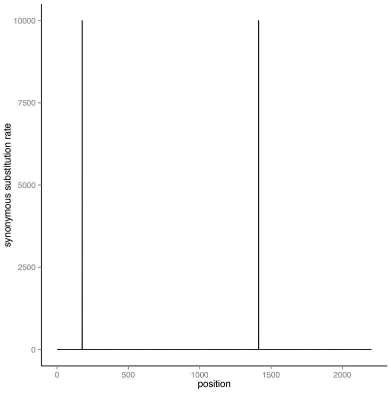

w=1

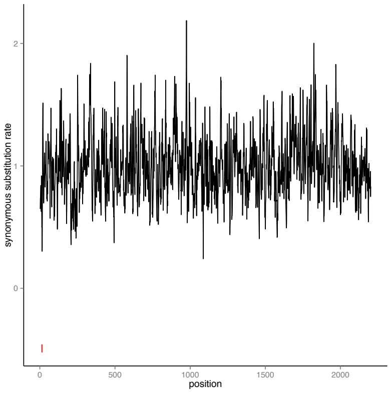

w=5

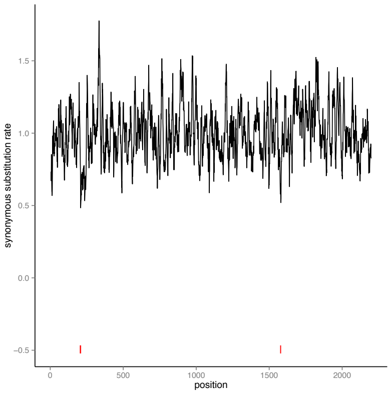

w=10

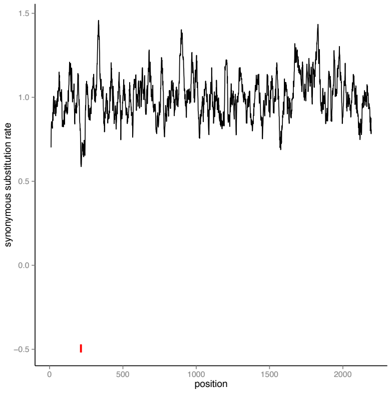

w=20

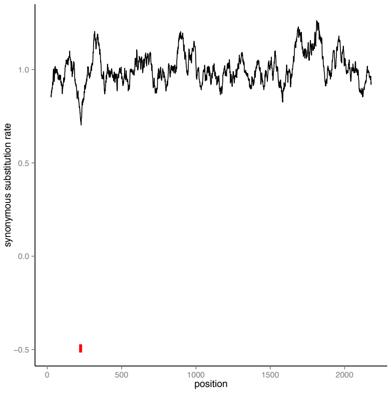

w=50

Newcastle M

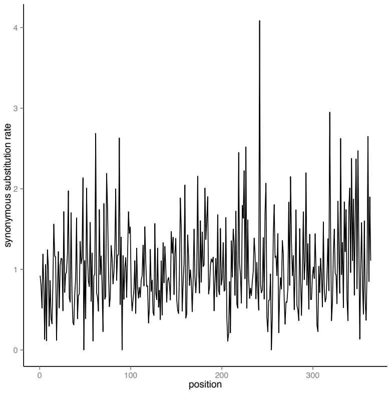

w=1

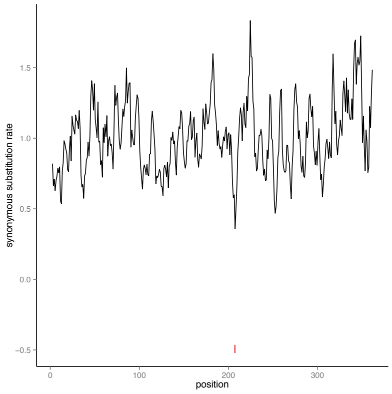

w=5

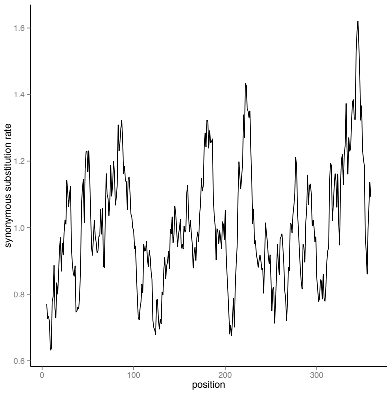

w=10

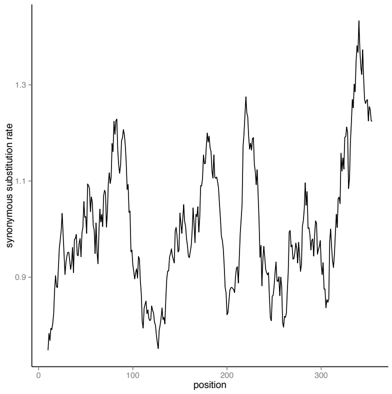

w=20

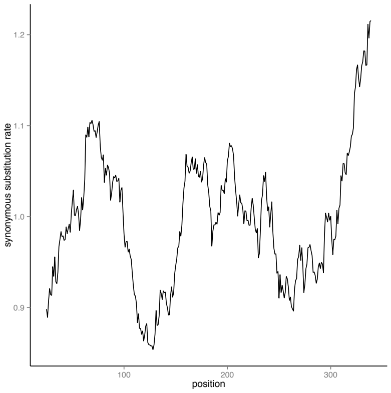

w=50

Newcastle NP

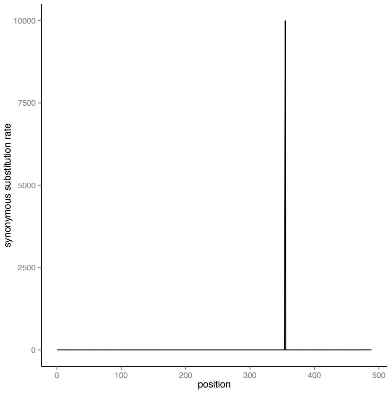

w=1

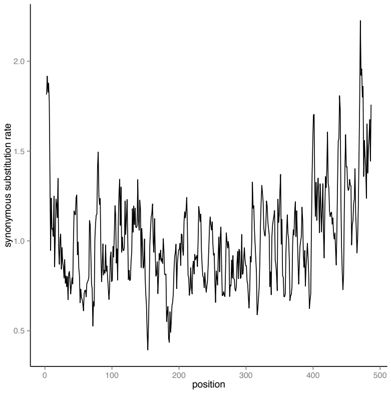

w=5

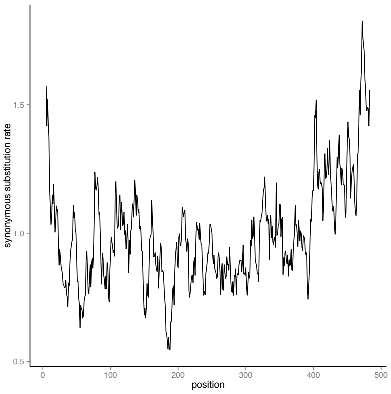

w=10

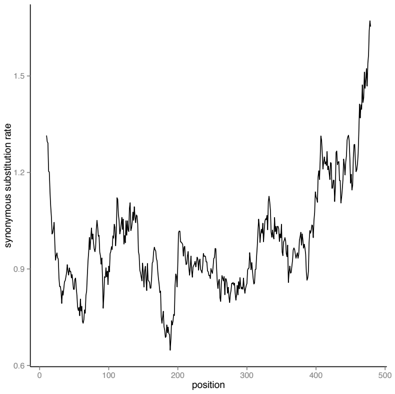

w=20

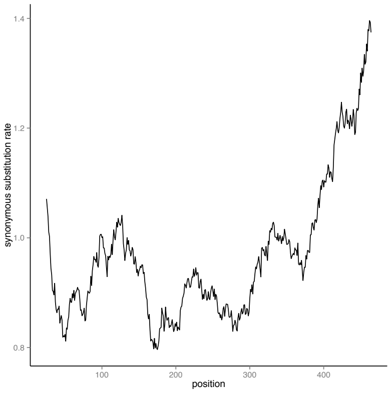

w=50

Newcastle P

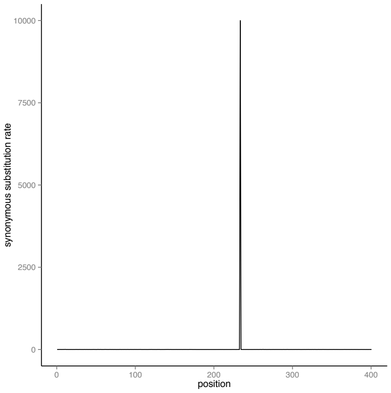

w=1

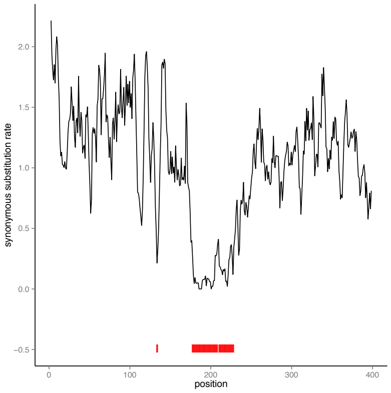

w=5

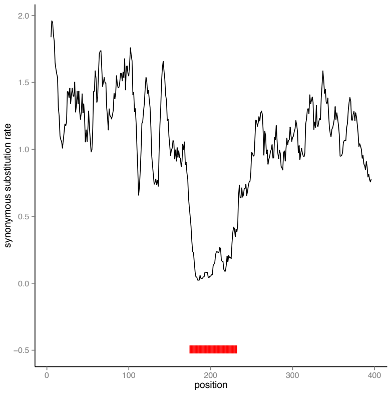

w=10

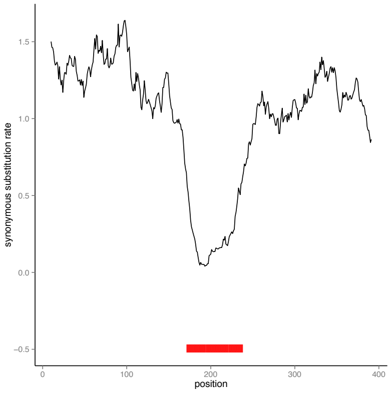

w=20

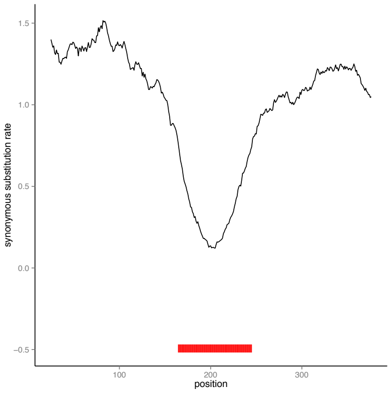

w=50

PCV rep

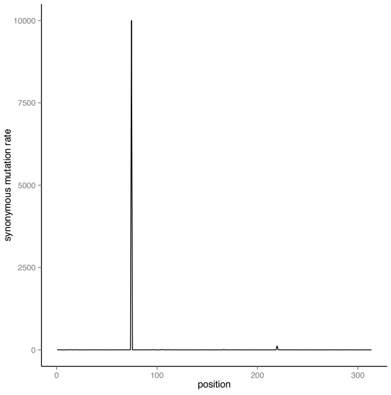

w=1

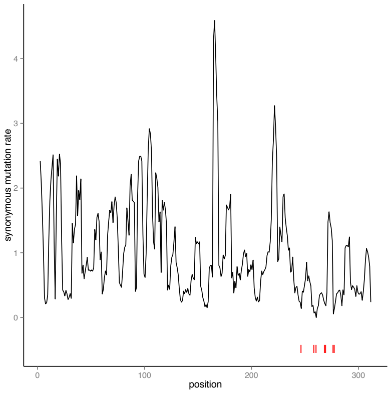

w=5

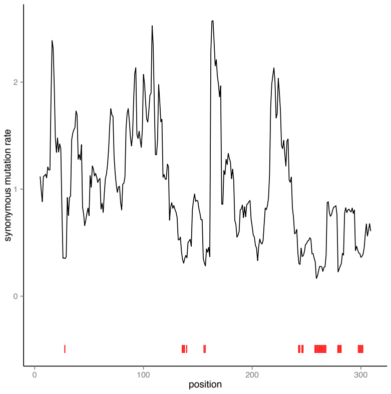

w=10

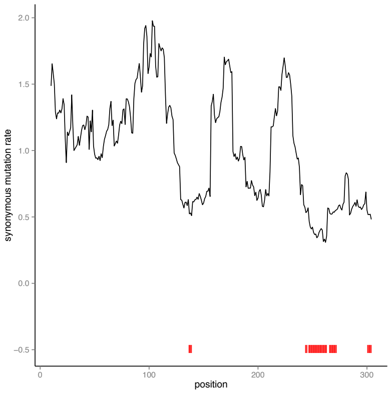

w=20

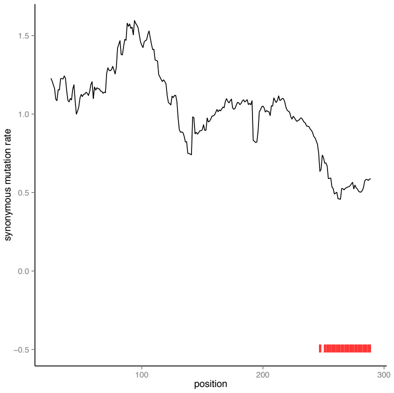

w=50

PCV cap

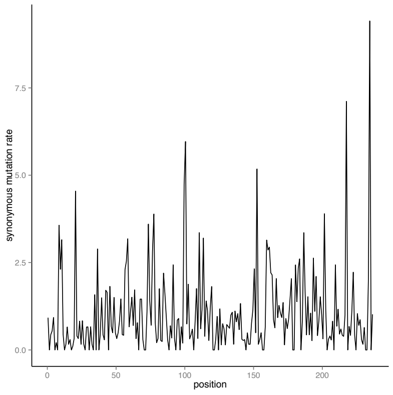

w=1

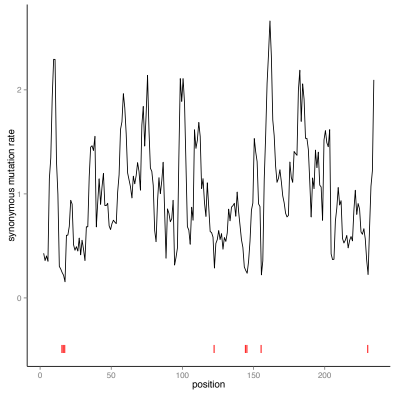

w=5

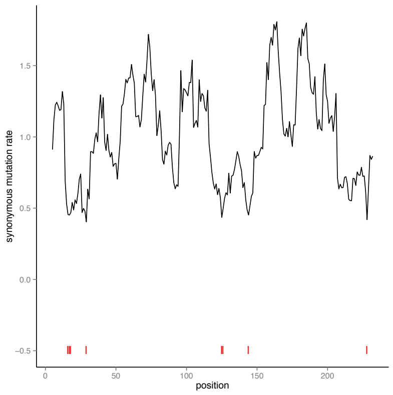

w=10

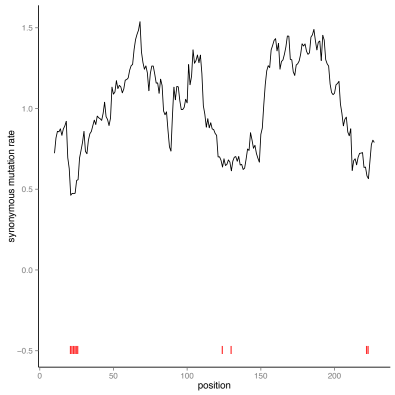

w=20

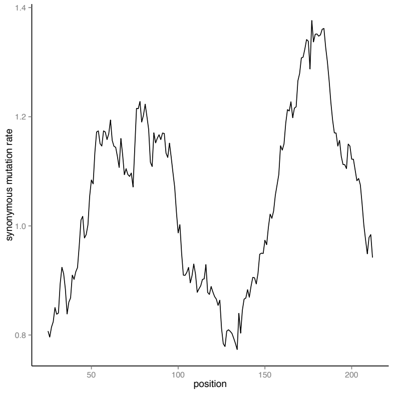

w=50

poliovirus

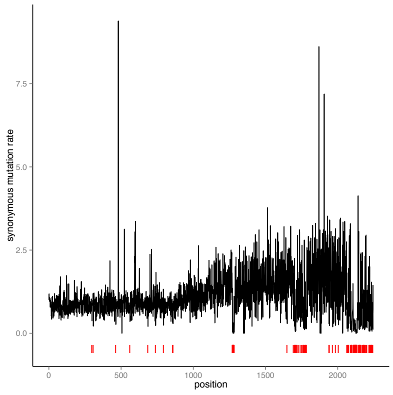

w=1

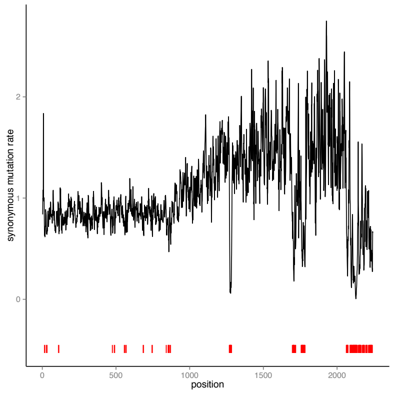

w=5

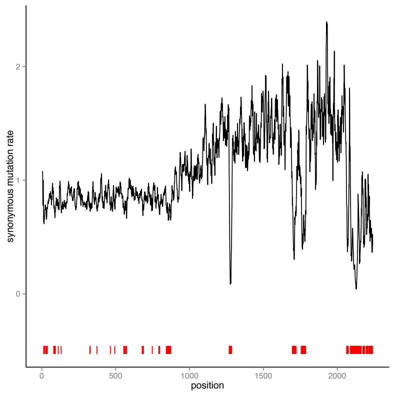

w=10

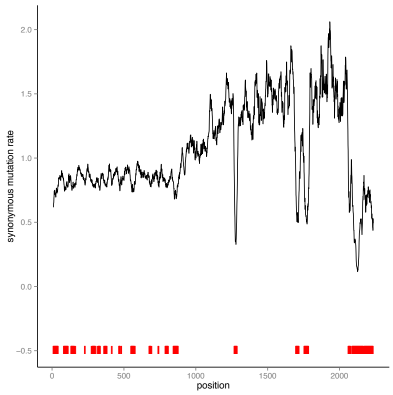

w=20

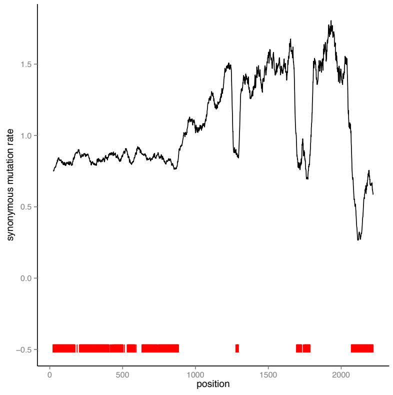

w=50

PVY polyprotein

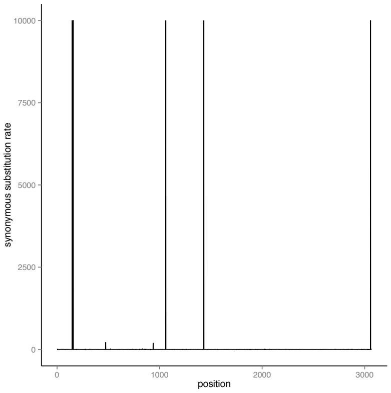

w=1

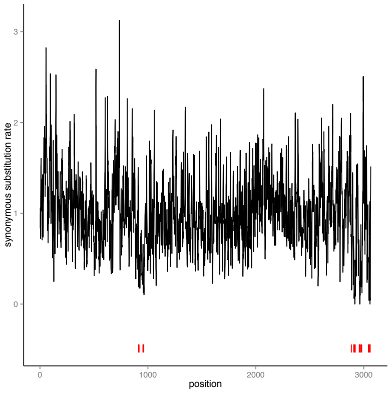

w=5

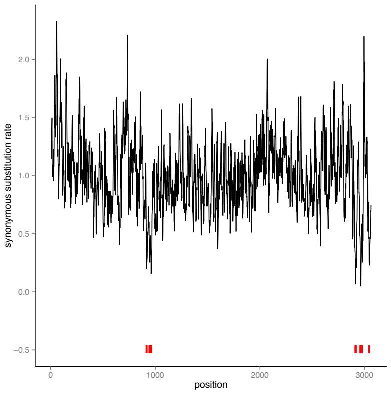

w=10

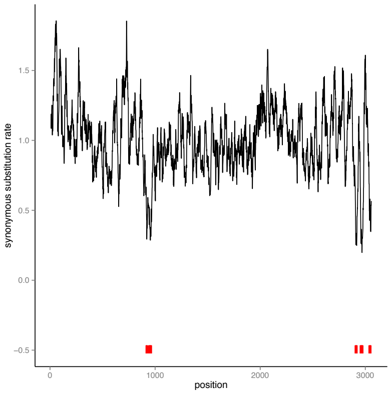

w=20

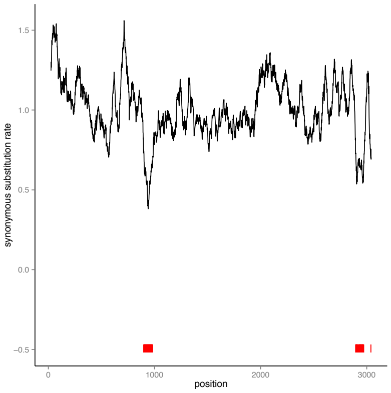

w=50

Rabies NP

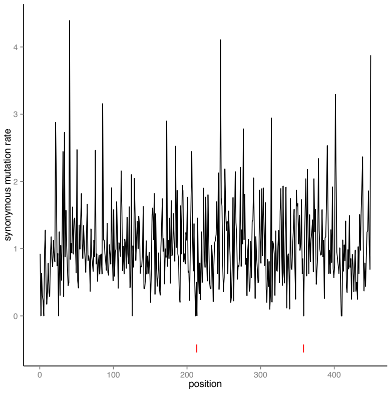

w=1

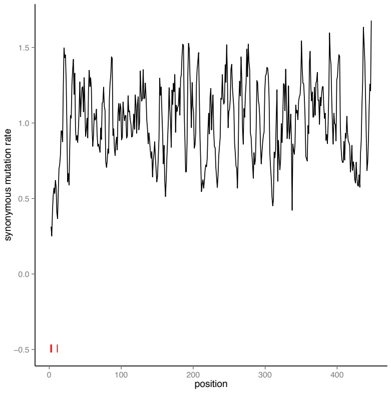

w=5

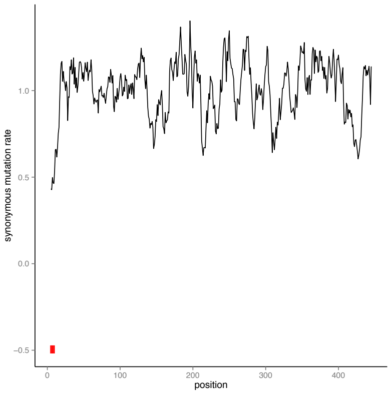

w=10

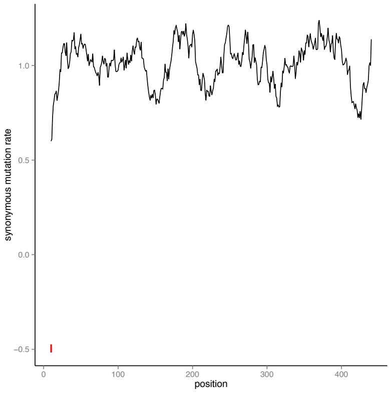

w=20

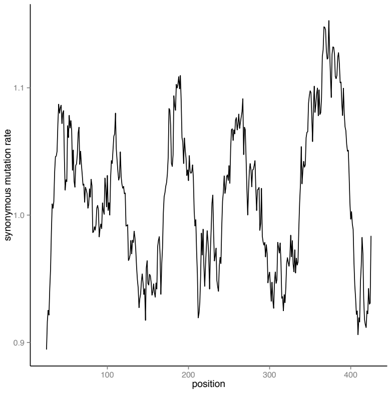

w=50

Rabies PP

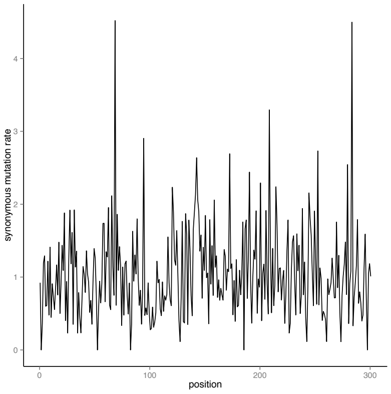

w=1

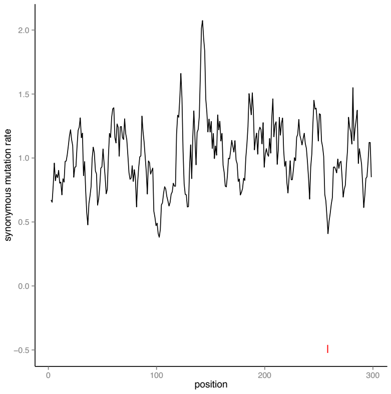

w=5

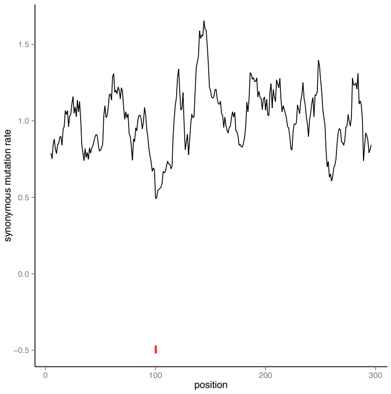

w=10

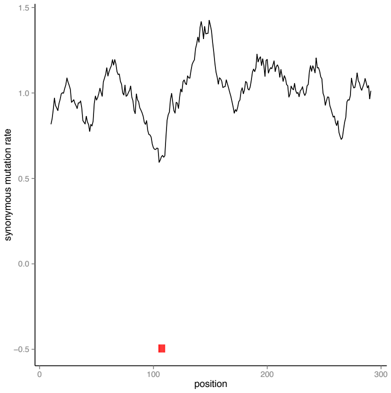

w=20

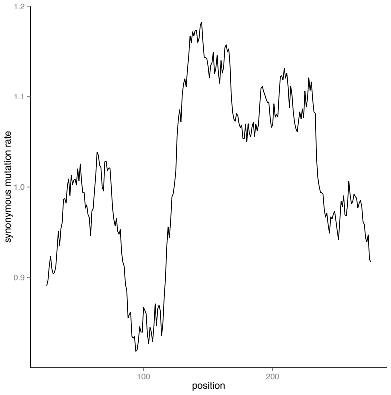

w=50

Rabies MP

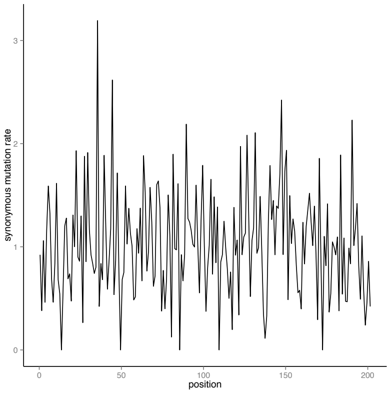

w=1

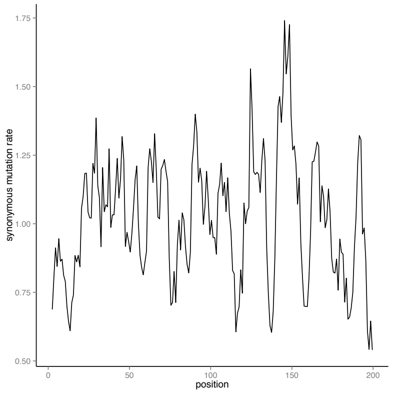

w=5

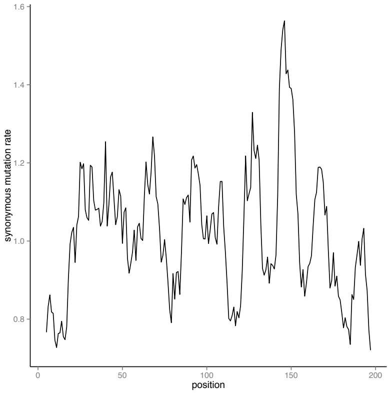

w=10

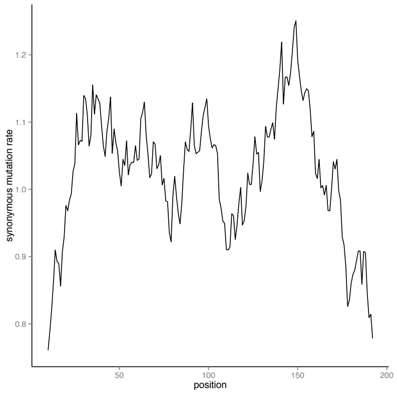

w=20

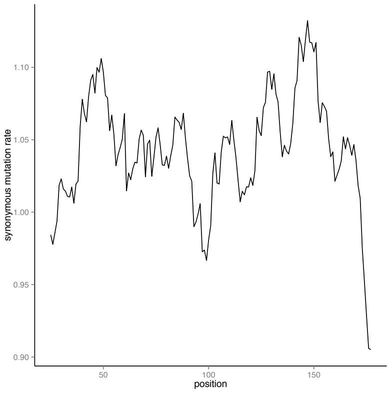

w=50

Rabies GP

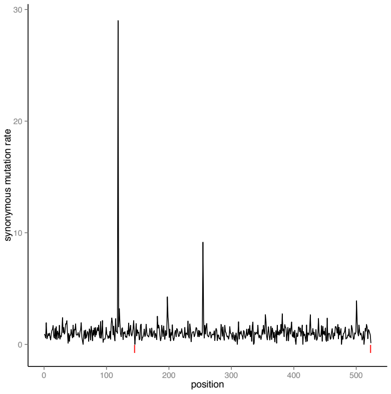

w=1

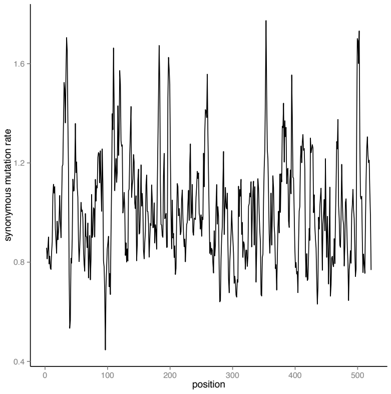

w=5

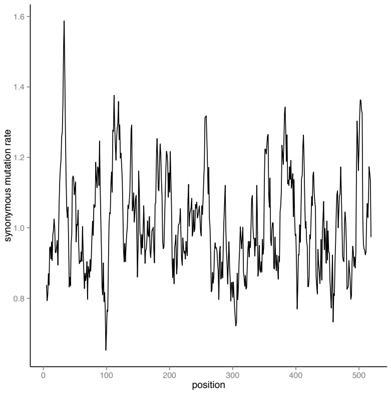

w=10

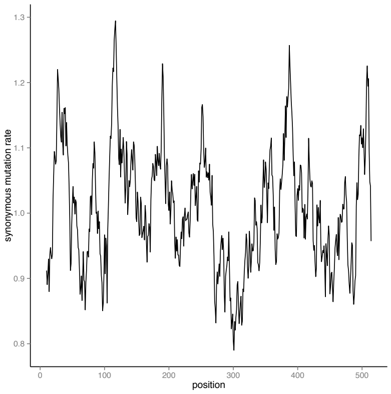

w=20

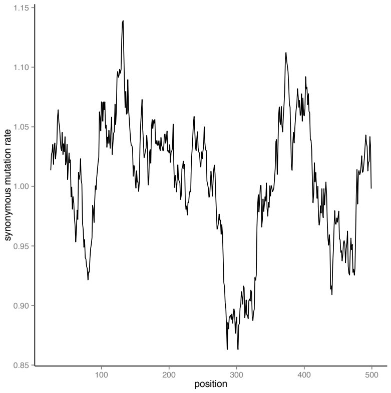

w=50

Rabies LP

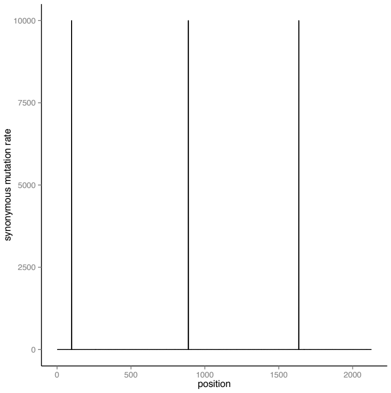

w=1

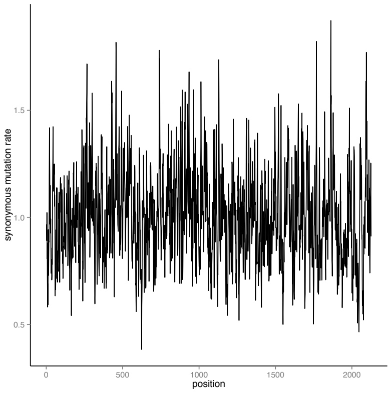

w=5

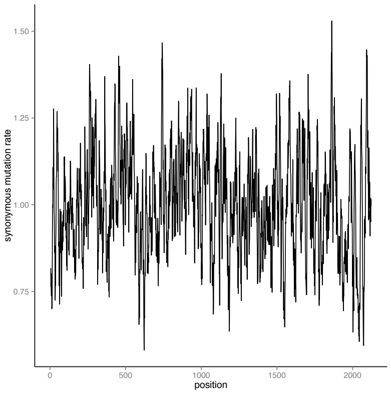

w=10

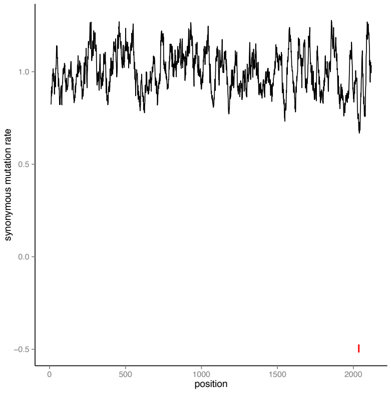

w=20

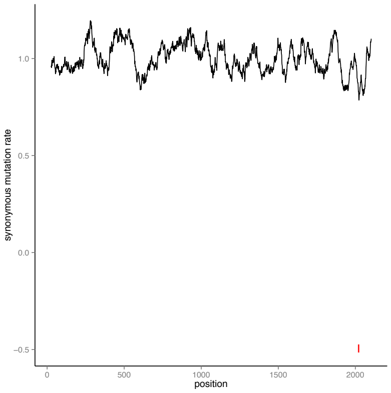

w=50

RFV glycoprotein

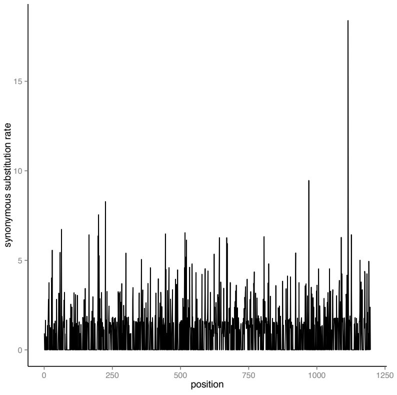

w=1

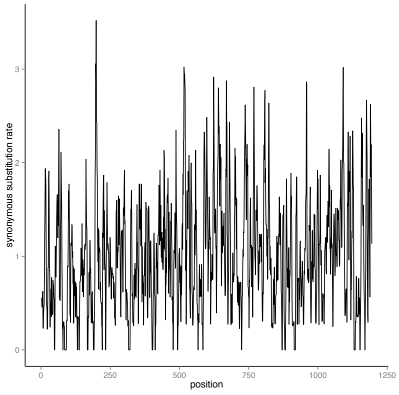

w=5

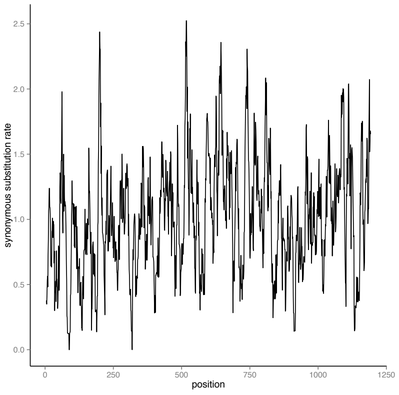

w=10

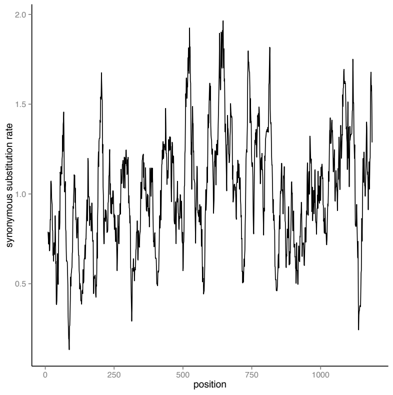

w=20

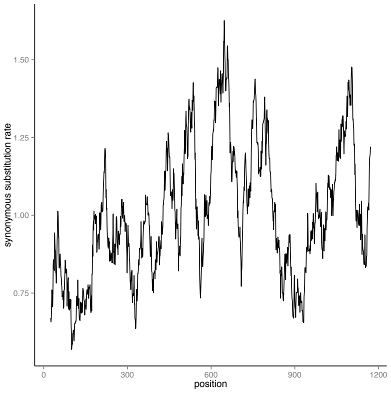

w=50

RFV nucleocapsid

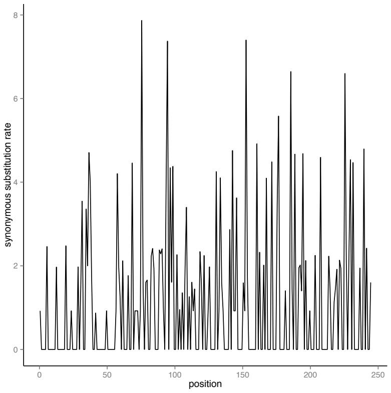

w=1

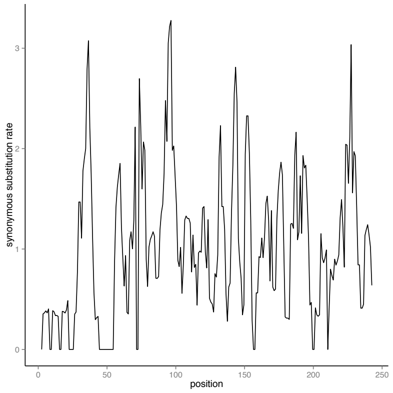

w=5

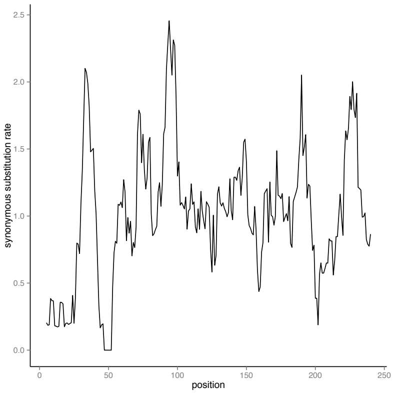

w=10

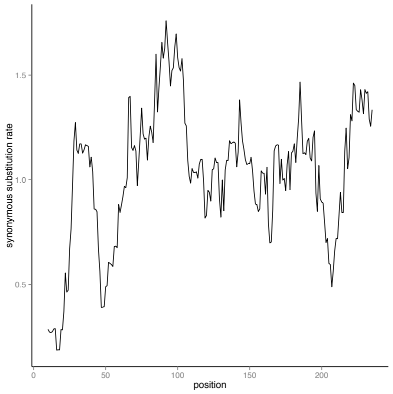

w=20

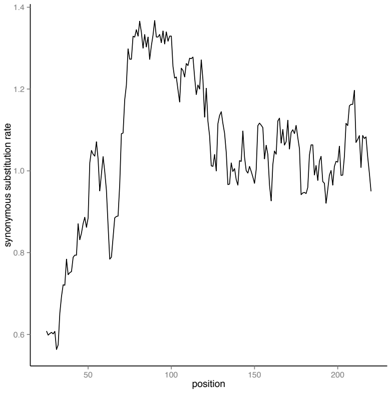

w=50

RFV polymerase

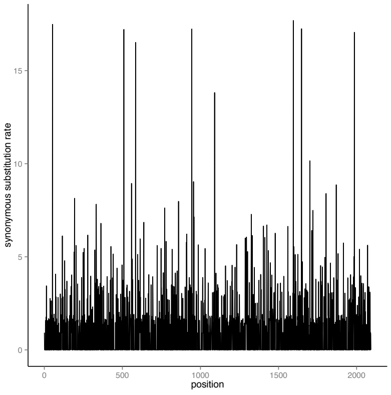

w=1

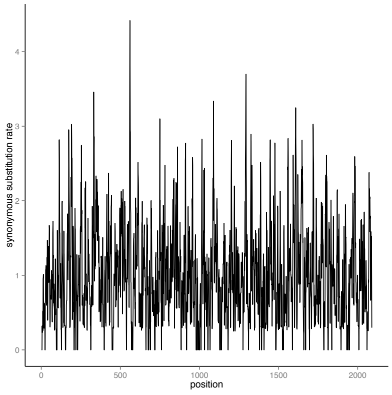

w=5

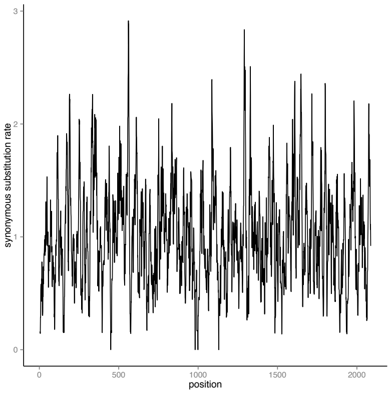

w=10

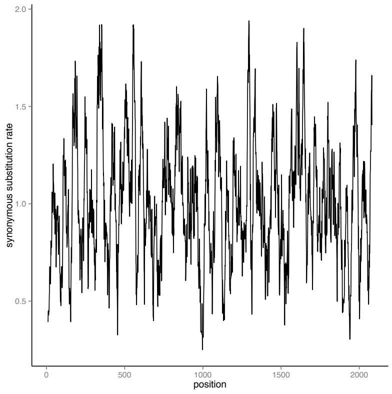

w=20

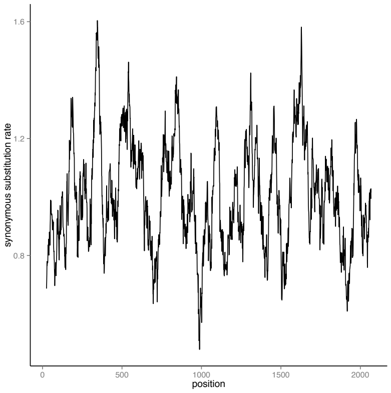

w=50

Rotavirus VP1

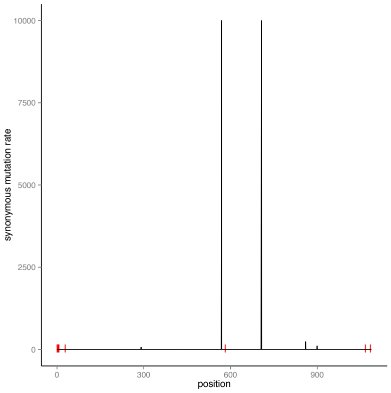

w=1

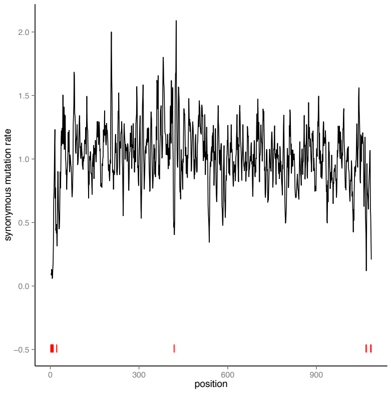

w=5

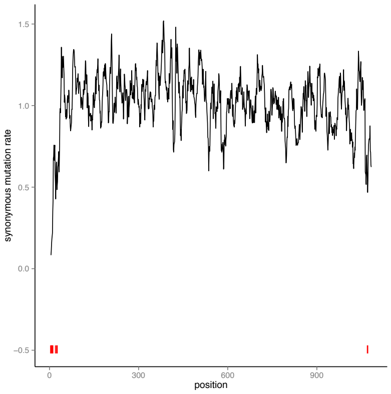

w=10

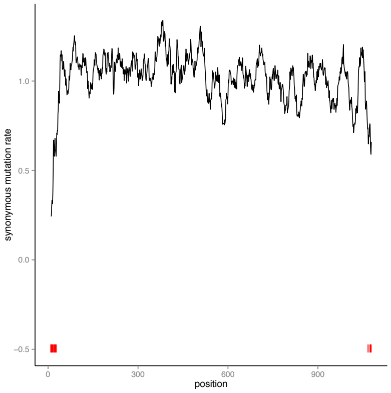

w=20

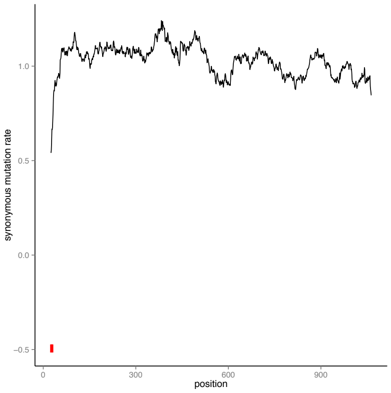

w=50

Rotavirus VP2

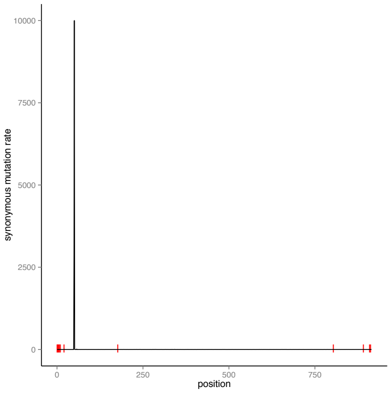

w=1

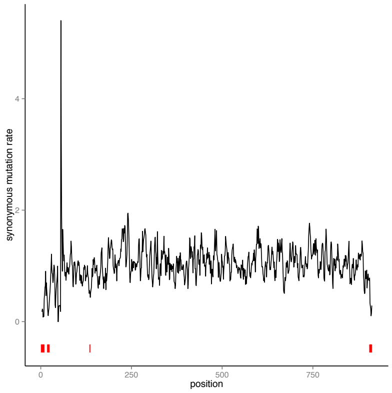

w=5

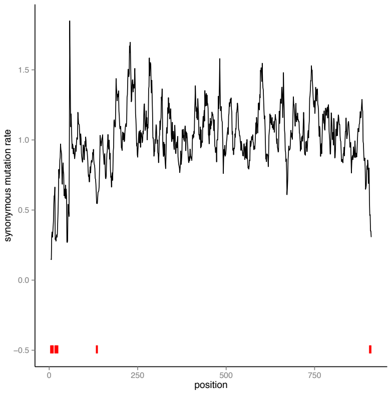

w=10

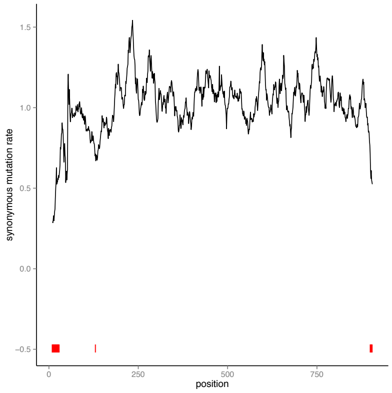

w=20

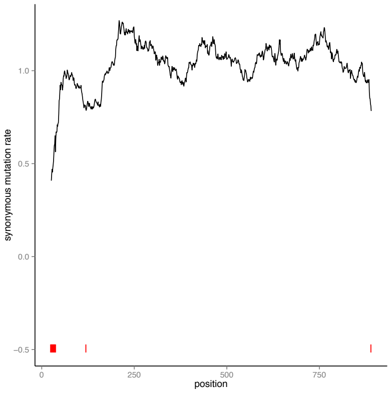

w=50

Rotavirus VP3

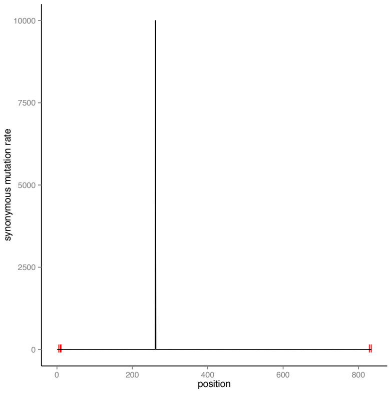

w=1

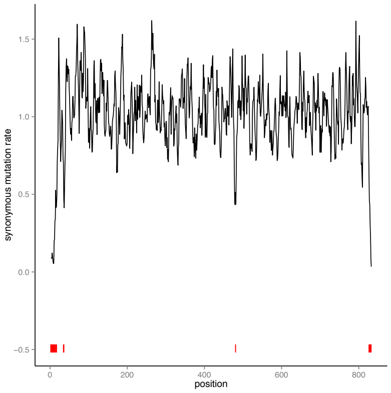

w=5

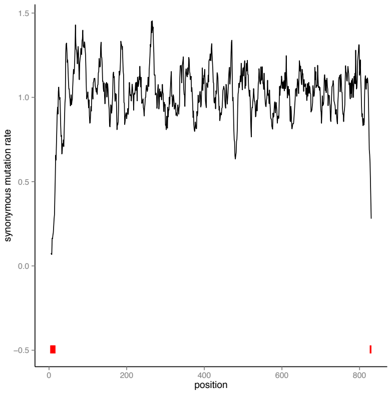

w=10

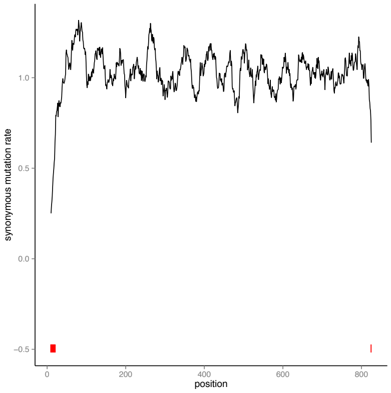

w=20

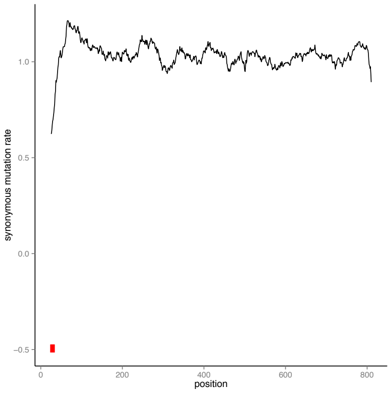

w=50

Rotavirus VP4

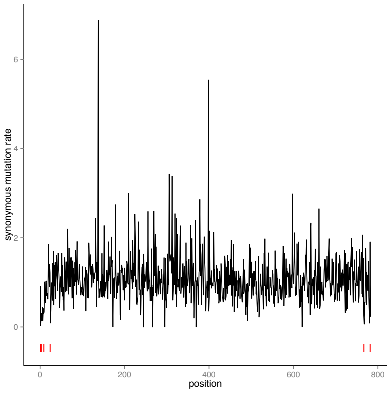

w=1

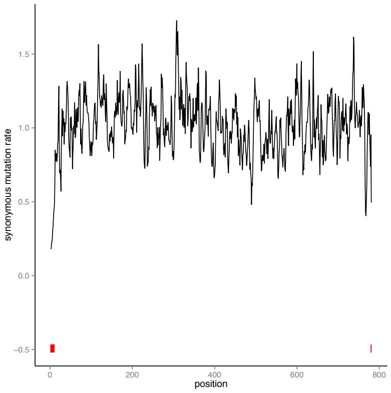

w=5

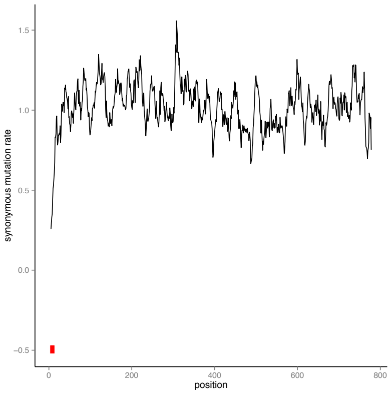

w=10

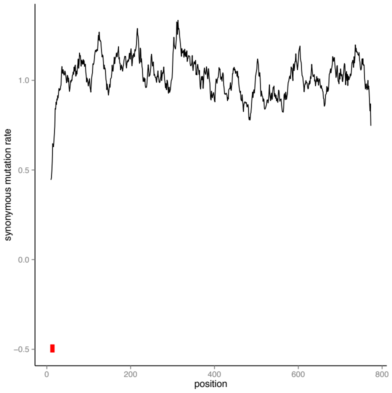

w=20

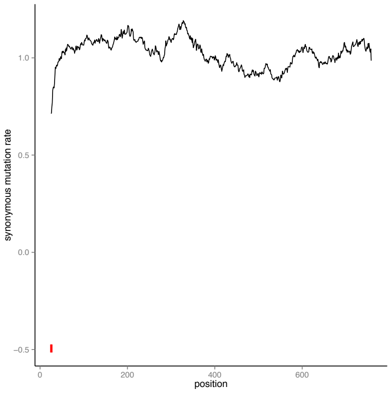

w=50

Rotavirus VP6

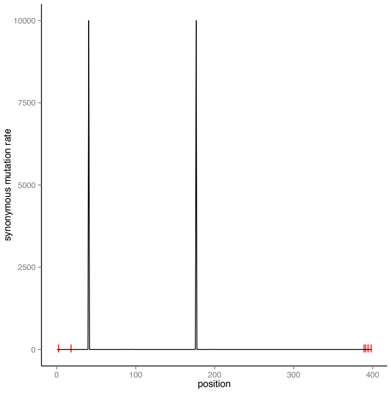

w=1

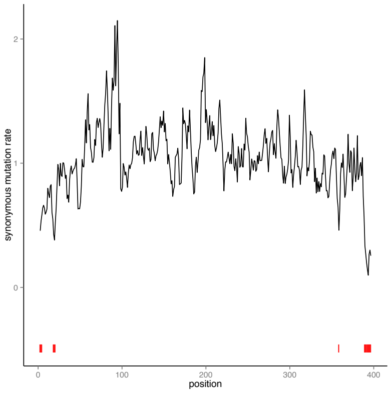

w=5

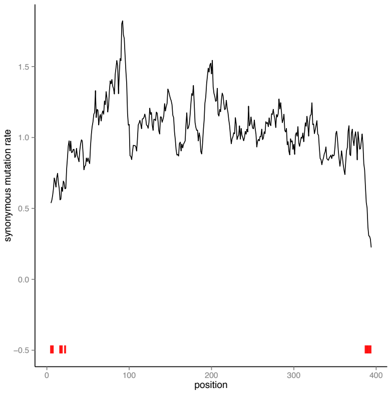

w=10

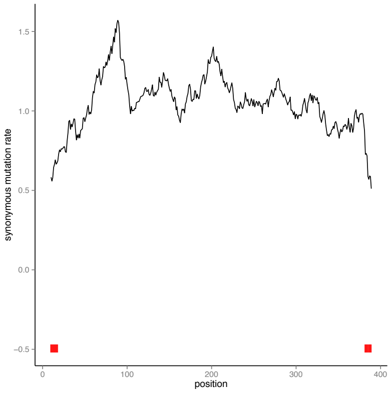

w=20

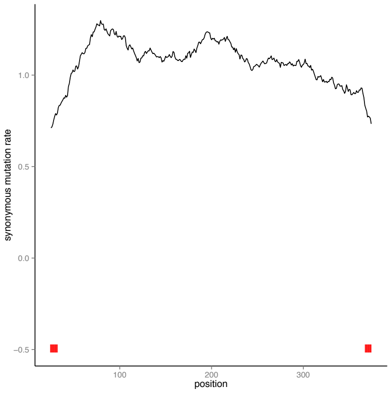

w=50

Rotavirus VP7

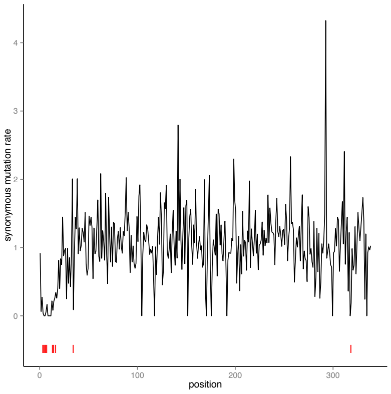

w=1

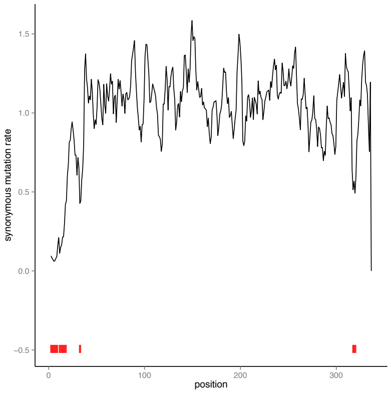

w=5

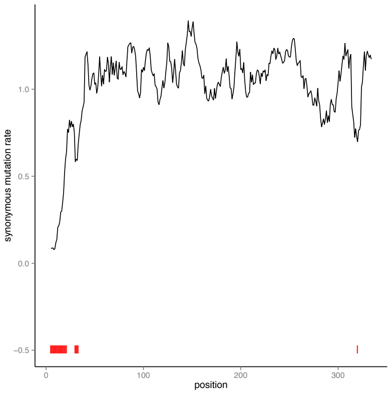

w=10

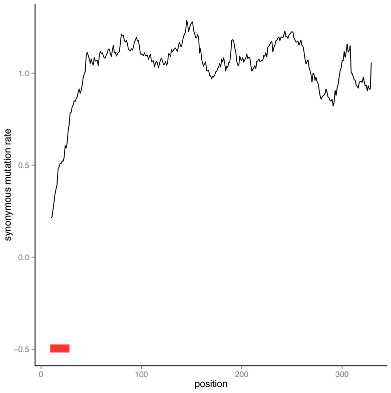

w=20

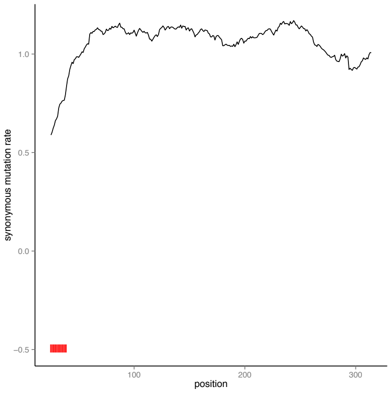

w=50

Rotavirus NSP1

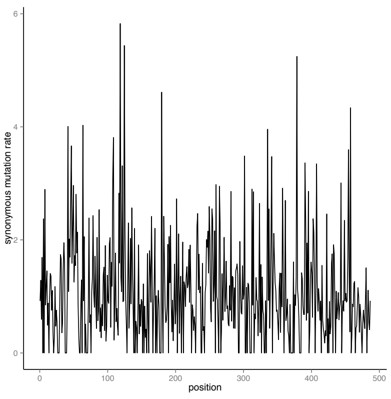

w=1

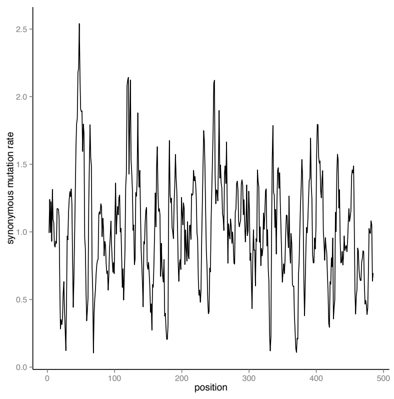

w=5

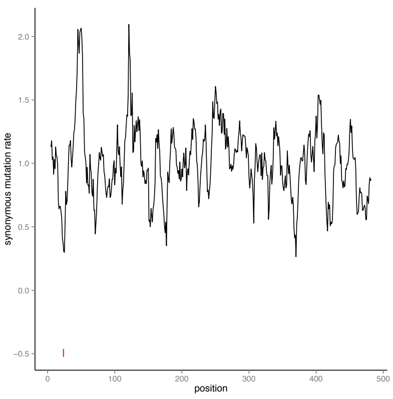

w=10

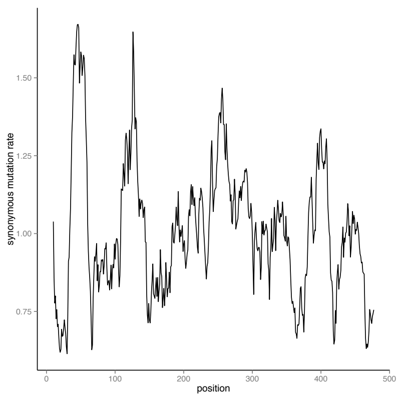

w=20

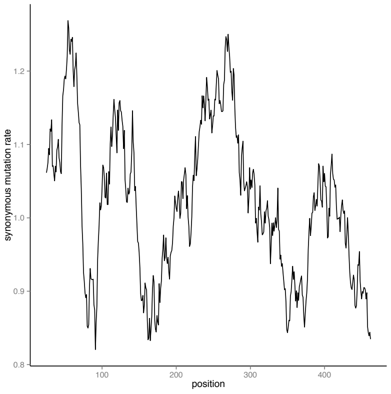

w=50

Rotavirus NSP2

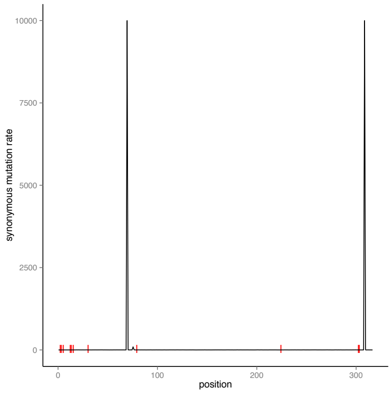

w=1

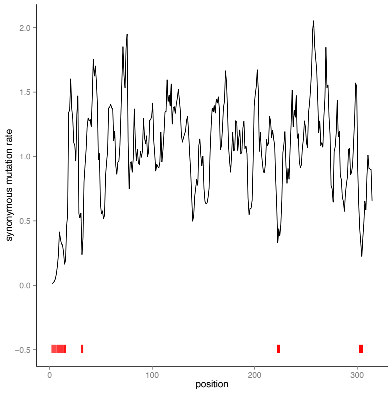

w=5

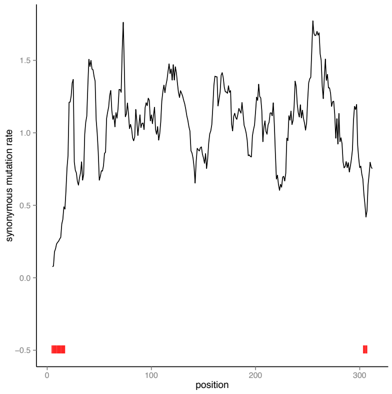

w=10

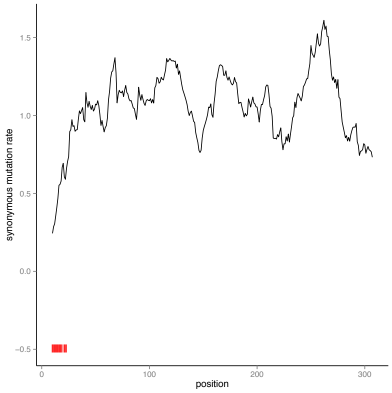

w=20

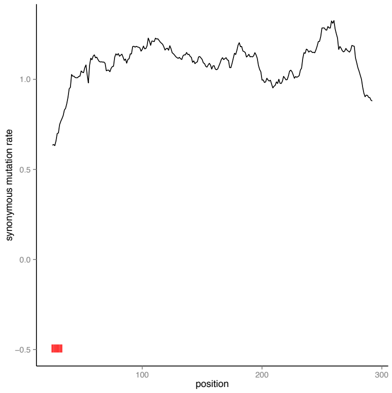

w=50

Rotavirus NSP4

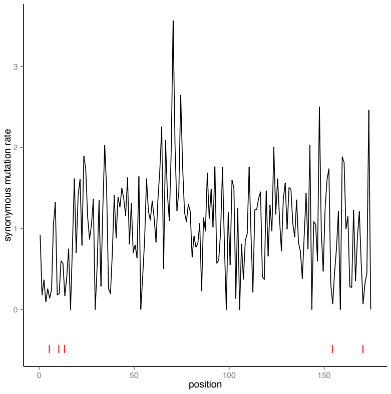

w=1

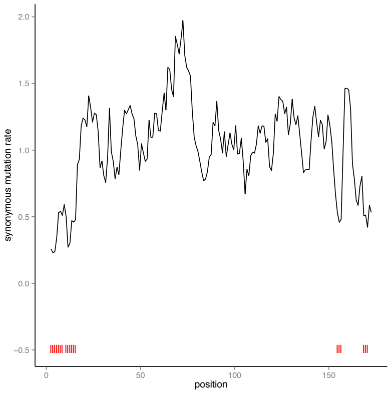

w=5

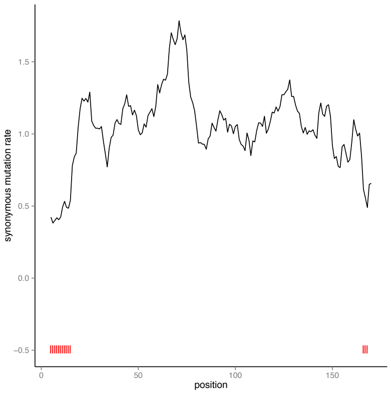

w=10

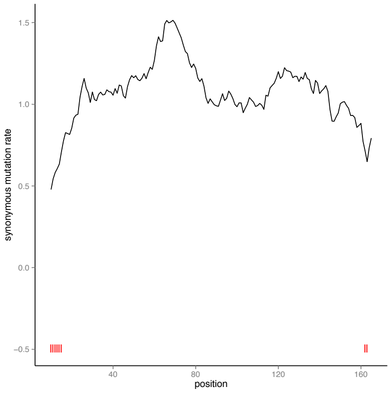

w=20

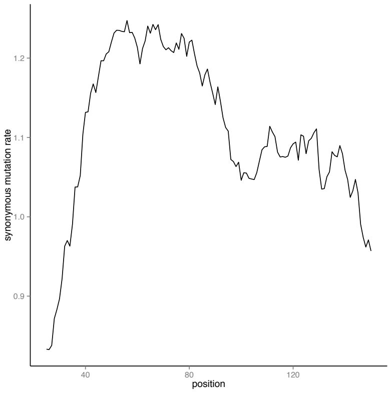

w=50

Rotavirus NSP5

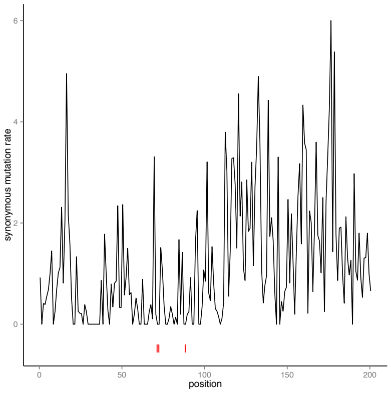

w=1

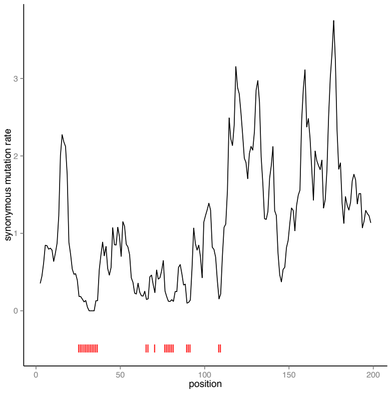

w=5

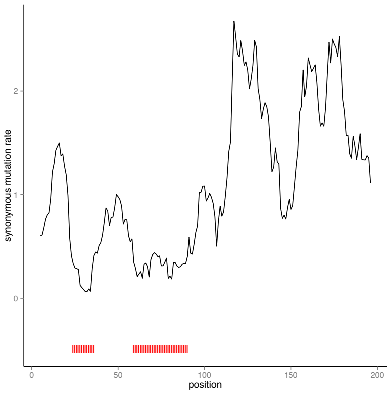

w=10

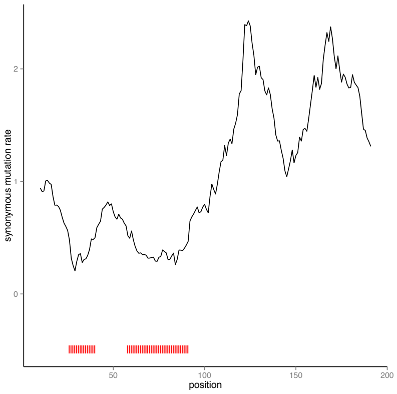

w=20

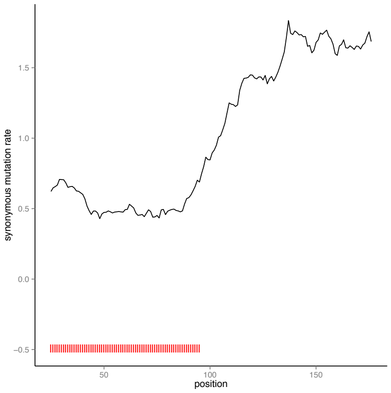

w=50

TBEV polyprotein

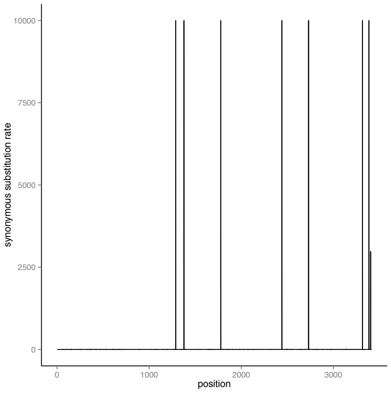

w=1

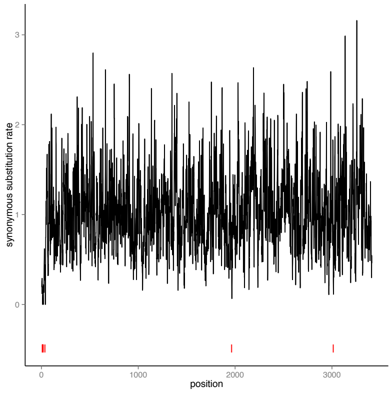

w=5

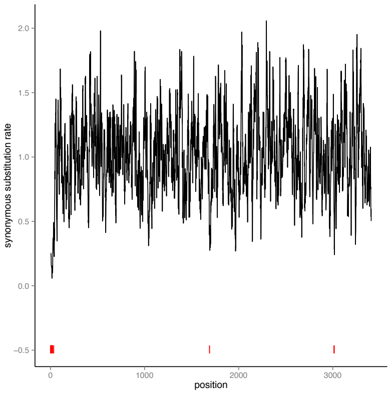

w=10

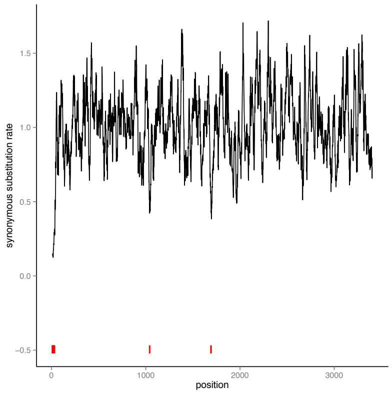

w=20

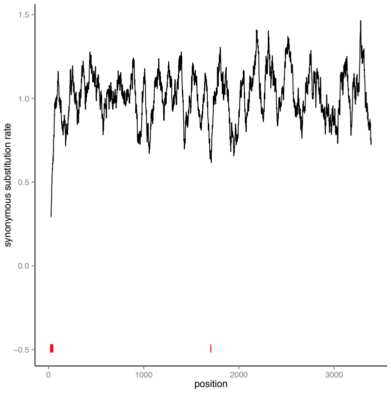

w=50

TuMV polyprotein

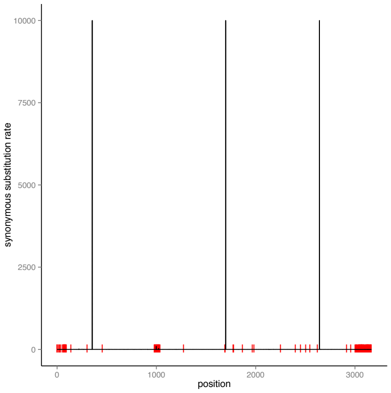

w=1

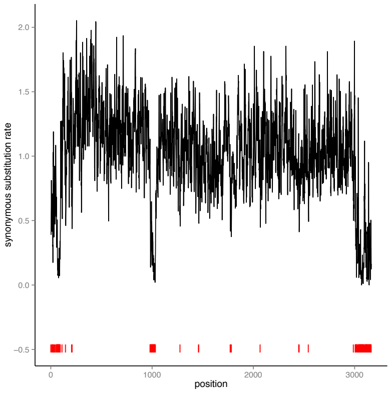

w=5

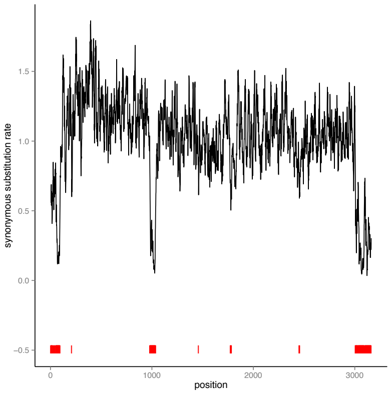

w=10

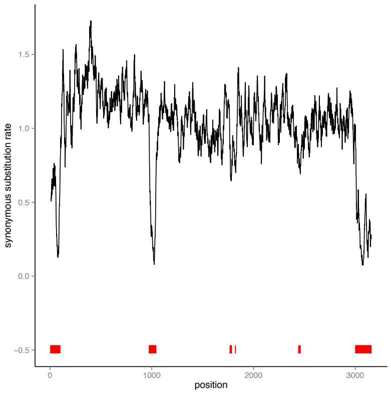

w=20

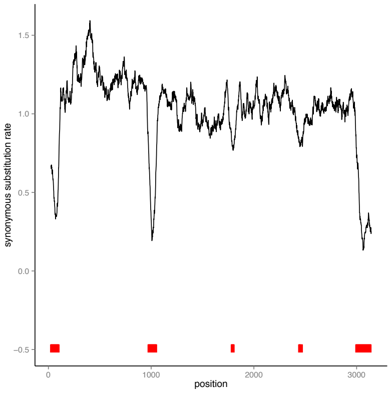

w=50

VEEV ns

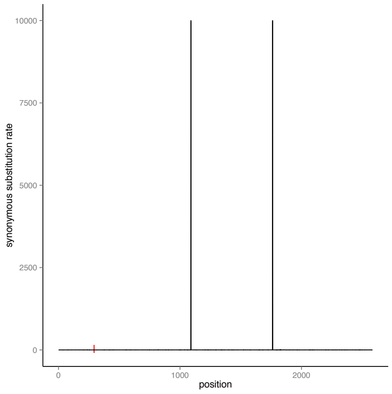

w=1

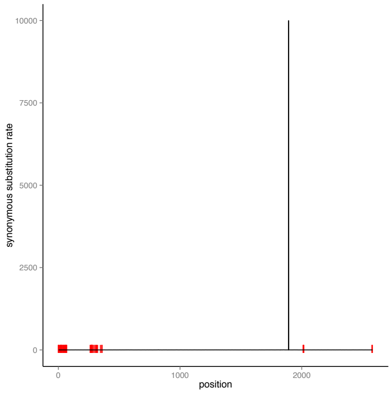

w=5

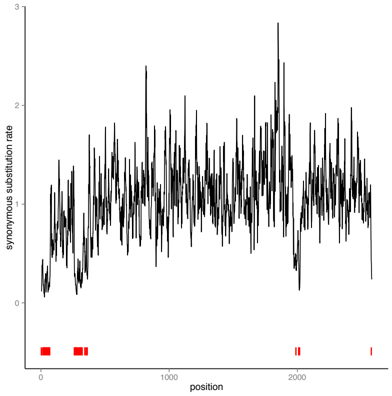

w=10

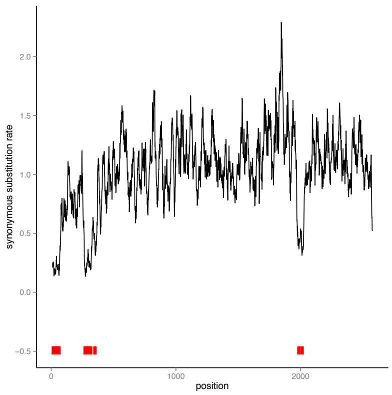

w=20

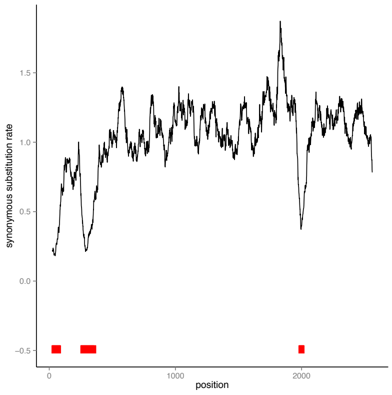

w=50

West Nile Virus

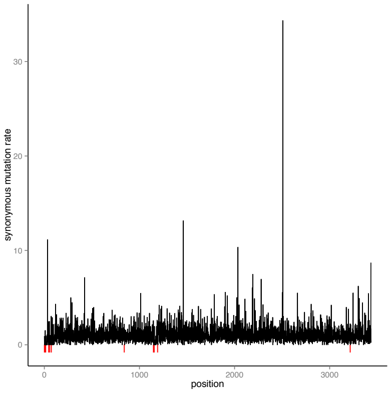

w=1

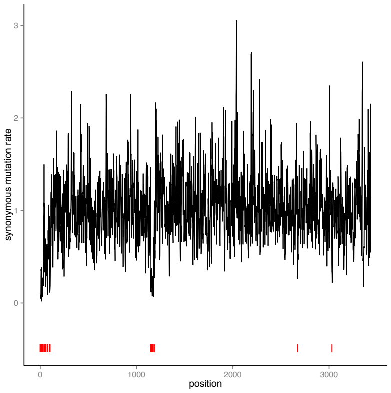

w=5

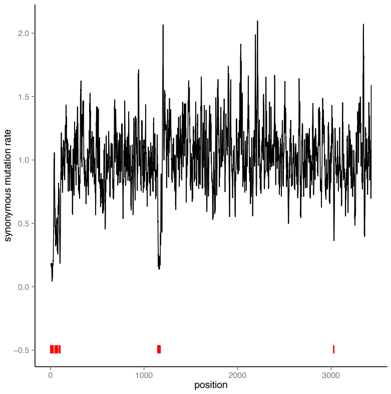

w=10

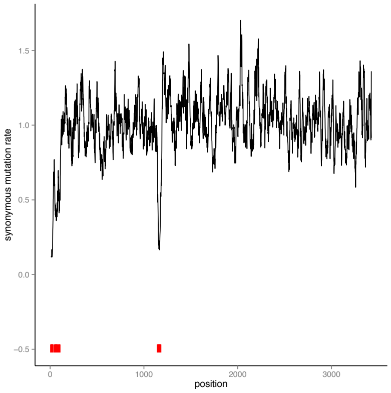

w=20

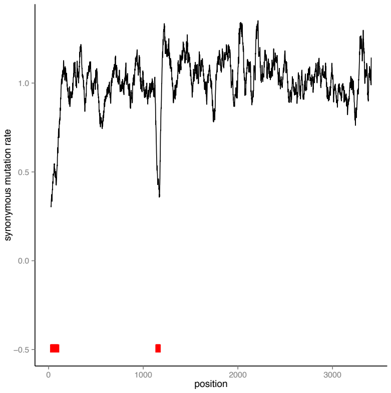

w=50
