# Supplementary material for: FRESCo: finding regions of excess synonymous constraint in diverse viruses
Source: Genome Biol. 2015 Feb 17;16(1):38. doi: 10.1186/s13059-015-0603-7 (PMC4376164; doi:10.1186/s13059-015-0603-7)
Supplement: Additional file 8: — Expanded description of FRESCo codon model. [file 13059_2015_603_MOESM8_ESM.pdf]

Supplementary File

Description of codon model

The codon model utilized was adapted from Lin et al. 2011. The main difference is that where Lin et al. used an empirical background model learned from codon substitution frequencies in a mammalian alignment, we use a parameterized background model with parameters learned from the viral alignment.

FRESCo takes as input a codon alignment and a phylogenetic tree. We first fit a HKY model of nucleotide evolution to the codon alignment, learning the transition/transversion ratio kappa. We also estimate substitution frequencies for every base pair in each codon position empirically based on the input alignment, as well as maximum-likelihood branch lengths for the input tree. We then fit an MG94 (Muse and Gaut, 1994) codon model to the full alignment, learning a nonsynonymous/synonymous substitution ratio R. The entry q_{ij} in the rate matrix Q, specifying a change at from codon i to codon j, is given by:

$$q_{ij} = \begin{cases} \kappa\pi_x^p & \text{(synonymous change,transition)} \\ \pi_x^p & \text{(synonymous change,transversion)} \\ R\kappa\pi_x^p & \text{(nonsynonymous change,transition)} \\ R\pi_x^p & \text{(nonsynonymous change,transversion)} \\ 0 & \text{(more than 1 change between codon i and codon j)} \end{cases}$$

, where R is the nonsynonymous/synonymous substitution ratio, κ is the transition/transversion ratio, and π_x^p is the (empirically determined) frequency for the target base pair x at the given codon position p. Equilibrium codon frequencies are determined using an F3X4 estimator.

We then run a sliding window across the alignment. For each sliding window, we fit null and alternate models. The null model has a window-specific nonsynonymous substitution rate, and the synonymous substitution rate is constrained to the genome-wide average:

$$q_0^{wnd} = \begin{cases} q_{ij} & \text{(synonymous change)} \\ \beta q_{ij} & \text{(nonsynonymous change)} \end{cases}$$

The alternate model has both window-specific synonymous and nonsynonymous substitution rates:

$$q_A^{wnd} = \begin{cases} \alpha q_{ij} & \text{(synonymous change)} \\ \beta q_{ij} & \text{(nonsynonymous change)} \end{cases}$$

To identify synonymous constraint elements, we then compare the two models using a likelihood ratio test to find the windows where the alternate model fits the data significantly better than the null model.
